# Coronary microvascular function and atherosclerotic plaque burden in ischaemia and no obstructive coronary arteries: a secondary analysis of the CorMicA trial

**DOI:** 10.1136/heartjnl-2024-324677

**Published:** 2024-11-27

**Authors:** Daniel TY Ang, Jaclyn Carberry, Thomas J Ford, Anna Kamdar, Robert Sykes, Novalia P Sidik, David Carrick, Peter J McCartney, Damien Collison, Keith Robertson, Aadil Shaukat, J Paul Rocchiccioli, R McGeoch, Stuart Watkins, Stuart Hood, Margaret McEntegart, Mitchell Lindsay, Hany Eteiba, Keith G Oldroyd, Richard Good, Alex McConnachie, Colin Berry

**Affiliations:** 1 Cardiology, Golden Jubilee National Hospital West of Scotland Regional Heart and Lung Centre, Glasgow, UK; 2 School of Cardiovascular and Metabolic Health, University of Glasgow College of Medical Veterinary and Life Sciences, Glasgow, UK; 3 University Hospital Hairmyres, East Kilbride, South Lanarkshire, UK; 4 Department of Cardiology, Central Coast Local Health District, Gosford, New South Wales, Australia; 5 Columbia University, New York, New York, USA; 6 Robertson Centre for Biostatistics, University of Glasgow, Glasgow, UK

**Keywords:** Atherosclerosis, Coronary Angiography, Microvascular Angina, Cardiovascular Diseases, Angina Pectoris

## Abstract

**Background:**

The relationship between atherosclerosis and endotypes of myocardial ischaemia with no obstructive coronary artery disease (INOCA) is unclear. We investigated potential associations between cumulative atherosclerotic plaque burden quantified using the Gensini score, novel invasive indices of coronary microvascular function (microvascular resistance reserve (MRR); resistive reserve ratio (RRR)) and related INOCA endotypes.

**Methods:**

Coronary angiography and invasive coronary function tests were simultaneously acquired in the CorMicA cohort. A comprehensive physiological assessment was performed using both a thermodilution-based diagnostic guidewire and intracoronary acetylcholine provocation testing. Angiograms were examined for luminal stenosis in each segment of the SYNTAX coronary model. Cumulative plaque burden was quantified using the Gensini score, which incorporated both the number of diseased coronary segments and stenosis severity. Results were compared with indices of microvascular function and INOCA endotypes. Angiographic analyses were performed blind to coronary physiology findings.

**Results:**

In 151 participants (median age 61 years; 73.5% female) without flow-limiting coronary artery disease, medical history included 41.7% smoking, 63.6% hypertension and 19.2% diabetes mellitus. The left anterior descending artery underwent diagnostic guidewire testing in 85.4%, and 55.0% of participants had abnormal coronary flow reserve (CFR) and/or Index of Microcirculatory Resistance (IMR). The median Gensini score was 6.0 (IQR 2.5–11.0). CFR (p=0.012), MRR (p=0.026) and RRR (p=0.026), but not IMR (p=0.445), were univariably associated with raised Gensini scores. These significant effects persisted in multivariable models controlling for potential confounders. Considering INOCA endotypes, Gensini scores differed among participants with microvascular angina (MVA) (7.0 (2.5–11.0)), vasospastic angina (VSA) (4.5 (2.0–10.0)), mixed MVA/VSA (9.0 (5.0–11.5)) and non-cardiac symptoms (3.5 (1.5–8.0)); Kruskal-Wallis p=0.030.

**Conclusions:**

Reduced CFR, MRR and RRR, and MVA were associated with increased coronary atherosclerotic plaque burden, as evidenced by higher Gensini scores. These novel findings provide a mechanistic link between INOCA and cardiovascular events, reinforcing the importance of antiatherosclerosis therapy in patients with MVA.

**WHAT IS ALREADY KNOWN ON THIS TOPIC**

- Epicardial coronary atherosclerosis of varying severity is prevalent among patients with ischaemia with no obstructive coronary artery disease (INOCA), although the pathophysiological associations between atherosclerosis and microvascular dysfunction remain uncertain.

**WHAT THIS STUDY ADDS**

- In this cross-sectional study of prospectively recruited individuals, clinical endotypes of INOCA were defined by coronary angiography, fractional flow reserve, thermodilution tests of microvascular function and responses to intracoronary infusion of acetylcholine. This was then correlated with the Gensini score, a quantitative measure of coronary plaque burden.
- Novel measures of coronary microvascular dysfunction, including reduced microvascular resistance reserve and resistive reserve ratio, and microvascular angina, were associated with higher Gensini scores.

**HOW THIS STUDY MIGHT AFFECT RESEARCH, PRACTICE OR POLICY**

- Our study identifies mechanistic associations between coronary atherosclerosis burden and microvascular dysfunction in INOCA, thereby reinforcing the importance of antiatherosclerosis therapy in this population.

## Introduction

 Many patients with suspected or proven myocardial ischaemia are found to have no flow-limiting disease in the epicardial circulation (ischaemia with no obstructive coronary artery disease (INOCA)).^1^ The Coronary Vasomotion Disorders International Study Group (COVADIS) have established criteria for the diagnosis microvascular and vasospastic angina (VSA).^2 3^ These conditions are prognostically important^1 4 5^ and, potentially, a therapeutic target.^6^ Guidelines recommend coronary function testing to aid clinical diagnosis.^1 7^ In the CorMicA trial,^8^ patients underwent invasive coronary function testing, to allow endotype classification according to COVADIS criteria^2 3^: microvascular angina (MVA), VSA, both, or non-cardiac chest pain.

Epicardial coronary atherosclerosis of varying severity is prevalent among patients with INOCA,^9^ although the pathophysiological associations between atherosclerosis and microvascular dysfunction remain uncertain. Echavarria-Pinto *et al*^9^ studied 78 patients with ‘intermediate’ coronary artery disease. Coronary flow reserve (CFR) and Index of Microcirculatory Resistance (IMR) differed according to the distribution of coronary artery disease (focal vs diffuse). However, this cohort was relatively small, 41% of patients had flow-limiting atherosclerosis (fractional flow reserve (FFR) ≤0.80), acetylcholine vasoreactivity testing was not included, and substantive analyses in the subgroup of patients with non-flow-limiting atherosclerosis (FFR >0.80; n=54 arteries) were uncertain. While CFR reflects the combined vasodilator reserve of the epicardial coronary artery and microcirculation, specific associations between atherosclerosis burden and novel microvascular-specific indices are uncertain.

To address these knowledge gaps, we aimed to investigate the relationships between atherosclerosis and microvascular function in a well-defined, prospectively recruited population. The microvascular resistance reserve (MRR)^10 11^ and resistive reserve ratio (RRR)^12 13^ are novel indices. Unlike CFR, they are specific measures of microvascular function and independent of epicardial coronary atherosclerosis. We investigated potential associations between cumulative atherosclerotic plaque burden quantified using the Gensini score, novel invasive indices of coronary microvascular function and related INOCA endotypes. We hypothesised MRR and RRR are inversely correlated with atherosclerosis burden in this population.

## Methods

### Study design

This was a retrospective analysis of the CorMicA trial population. The trial design, 6- and 12-month results have been published.^8 14 15^ The study is an investigator-led, parallel-group, randomised, controlled trial with a blinded outcome assessment. Participants with no obstructive coronary artery disease (<50% luminal diameter stenosis on angiography, or FFR >0.80) underwent an interventional diagnostic procedure at the time of invasive coronary angiography.

### Participants

Participants were recruited from elective, adult referrals to two regional cardiac centres in the west of Scotland (population of 2.5 million). All outpatients aged ≥18 years who were undergoing clinically indicated invasive coronary angiography were eligible. Exclusion criteria were non-coronary indications for angiography (eg, valvular disease) and inability to provide informed consent. Only participants with non-flow limiting coronary artery disease were included in this study.

### Interventional diagnostic procedure and indices of microvascular function

The full details of the IDP protocol are available in online supplemental file 1.^14^ It comprised two components: assessment with a diagnostic guidewire and vasospasm provocation with a pharmacological agent.

First, a combined pressure/temperature diagnostic guidewire (PressureWire X, Abbott Cardiovascular) was advanced to the distal (>6 cm from ostium) coronary artery. The left anterior descending (LAD) was selected as default unless technical or clinical reasons (eg, tortuous anatomy) advocated choosing a different coronary artery. Intracoronary short-acting nitrate (GTN 200 µg) was administered to attenuate any resting spasm. Using the principles of thermodilution, rapid boluses of 3 mL room temperature normal saline were performed to obtain transit times at rest. Intravenous adenosine infusion (140 µg/kg/min) was commenced to induce systemic hyperaemia and thermodilution injections repeated. Haemodynamic responses were recorded using linked software (CoroFlow V.3.01, CoroVentis) and used to calculate CFR (abnormal <2.0) and IMR (abnormal ≥25).

The MRR and RRR were retrospectively calculated for all subjects, using previously obtained physiology data. The MRR is specific for the microvasculature, independent of autoregulation and myocardial mass.^11^ It is calculated by dividing the CFR by FFR and correcting for driving pressures. RRR is a marker of the capacity of the coronary microcirculation to reduce resistance in response to hyperaemia. It is the ratio of the resting to hyperaemic resistance index.^13^

Finally, pharmacological provocation testing for spasm was undertaken with sequential 2 minute intracoronary acetylcholine infusions of incremental concentrations (10^−6^ M, 10^−5^ M, 10^−4^ M), followed by a bolus (100 µg for left or 50 µg for right coronary artery (RCA)).

### Diagnosis of clinical endotypes

Each INOCA endotype is characterised by distinct functional or pathophysiological mechanisms. A reduced CFR (<2.0) reflected impaired vasodilator reserve (functional microvascular dysfunction), whilst an elevated IMR (≥25) reflected microvascular dysfunction due to structural remodelling. The vasoactive response to intracoronary infusion of acetylcholine disclosed epicardial coronary and/or microvascular spasm, or a normal response.

According to COVADIS criteria,^2^ MVA is diagnosed with an abnormal response during either the diagnostic guidewire test (CFR and/or IMR) and/or pharmacological provocation (slow flow phenomenon, ischaemic ECG changes and chest pain). VSA is supported by epicardial coronary spasm (>90% diameter reduction) with chest pain and ECG changes.^3^ Mixed MVA and VSA is diagnosed if features from both conditions are present. Non-cardiac symptoms are diagnosed if the diagnostic guidewire and pharmacological tests are normal.

### Angiographic analysis

The spatial resolution of invasive coronary angiography is approximately 0.3 mm. Clinician visual assessment of the angiogram is the standard approach for quantifying coronary atherosclerotic plaque. While less sensitive than cross-sectional imaging, detection of luminal stenosis is nonetheless specific for atherosclerotic plaque.^16 17^

The coronary angiograms were analysed (Carestream Vue PACS, V.12.2.2.1025) by two clinicians trained in angiographic interpretation (DTYA and JC). Stenosis severity was determined by visual assessment, from at least two angiographic views at least 30 degrees projection apart, and scored based on the highest luminal stenosis identified in that segment. The assessment was made for all coronary segments sized >2.0 mm diameter, with each segment defined according to the SYNTAX coronary anatomy map.^18^

Interobserver disagreements were resolved through a joint review of images and/or adjudication by a third clinician (CB). All angiographic assessments and subsequent Gensini scoring were performed while blind to the results of the coronary physiology findings (ie, CFR, IMR, MRR and RRR).

### Assessing atherosclerotic plaque distribution and burden: the Gensini score

First, the atherosclerotic plaque distribution was defined according to the aggregated number of SYNTAX coronary segments with luminal stenosis ≥1% evident on the angiogram. Then, the Gensini score was calculated as follows. The Gensini score was first described in 1983^19^ and later summarised by Rampidis *et al*,^20^ as a cumulative quantification of both plaque distribution and severity. The Gensini score considers the full spectrum of atherosclerotic stenosis, with increasing points allocated based on stenosis severity (score 0 for no stenosis, 1 for 1–25% stenosis, 2 for 26–50%, 4 for 51–75%, 8 for 76–90%, 16 for 91–99%, 32 for chronic total occlusion).

As all participants in this study had no obstructive coronary artery disease, the maximum unweighted score anticipated per coronary segment was 4 (moderate luminal stenosis with FFR >0.80). Next, a weighting factor was applied to the initial score according to the coronary segment involved (eg, left main stem x5, proximal circumflex x3.5 if left dominant circulation or x2.5 if right dominant, x0.5 for posterior left ventricular branch).^20^ Finally, the Gensini score was the sum of the weighted stenosis scores for all coronary segments. These scores were investigated for associations with indices of microvascular function (CFR, IMR, MRR, RRR) and compared between the diagnosed clinical endotypes.

### Statistical methods

Continuous variables are presented as mean+/-SD or median (IQR), according to their normal or non-normal distribution. Categorical variables are presented as numbers and percentages. Distributions were assessed using normal Q-Q plots. Continuous variables were compared with the *t*-test, Mann-Whitney *U*-test, or Kruskal-Wallis H-test, as appropriate. Categorical variables were compared with Fisher’s exact test. Correlation between indices of microvascular function was assessed using Pearson’s or Spearman’s coefficient, according to their normal or non-normal distribution. Univariable and multivariable linear regression were conducted to identify predictors of the Gensini score. Covariates were chosen on clinical grounds and included in all models regardless of statistical significance. Modelling assumptions were checked by visual inspection of residuals, using Q-Q plots. Models were checked for multicollinearity by examination of variance inflation factors (acceptable if VIF <4). A p value of <0.05 was considered significant. Statistical analyses were performed using SPSS software V.29.0.1.0 (IBM, Armonk, New York, USA).

### Patient and public involvement

It was not possible to involve patients or the public in the design, conduct, reporting, or dissemination plans of our research.

## Results

Between 25 November 2016 and 12 November 2017, 151 participants with symptoms but no obstructive coronary artery disease underwent the IDP according to the CorMicA protocol.

### Baseline demographics

Clinical characteristics are summarised in table 1. The median age was 61 years (IQR 53–68), and 73.5% (n=111) were female. The calculated ASSIGN score for the 10-year likelihood of a cardiovascular event was similar between coronary endotype groups.

**Table 1:** Clinical characteristics and medication prescription at the time of coronary function testing, grouped according to final clinical diagnosis

Characteristics	MVA (n=78)	VSA (n=25)	Mixed MVA and VSA (n=31)	Non-cardiac symptoms (n=17)	P value
Age (years)	62 (IQR 53–68)	54 (IQR 51–65)	63 (IQR 58–70)	58 (IQR 52–65)	0.085
Sex (female)	60/78 (76.9%)	15/25 (60.0%)	22/31 (71.0%)	14/17 (82.4%)	0.316
Smoking history	29/78 (37.2%)	14/25 (56.0%)	18/31 (58.1%)	2/17 (11.8%)	0.009
Hypertension	53/78 (67.9%)	11/25 (44.0%)	21/31 (67.7%)	11/17 (64.7%)	0.180
Diabetes mellitus	11/78 (14.1%)	7/25 (28.0%)	5/31 (16.1%)	6/17 (35.3%)	0.132
Atrial fibrillation	5/78 (6.4%)	1/25 (4.0%)	3/31 (9.7%)	0/17 (0.0%)	0.739
Previous MI	8/78 (10.3%)	5/25 (20.0%)	10/31 (32.3%)	1/17 (5.9%)	0.026
Previous PCI	7/78 (9.0%)	5/25 (20.0%)	7/31 (22.6%)	0/17 (0.0%)	0.052
Previous CVA	9/78 (11.8%)	5/25 (20.0%)	4/31 (12.9%)	2/17 (11.8%)	0.728
Peripheral arterial disease	3/78 (3.8%)	3/25 (12.0%)	2/31 (6.5%)	0/17 (0.0%)	0.301
Cholesterol (mmol/L)	3.28 (2.78–4.10)	3.12 (2.73–3.78)	3.34 (2.82–4.35)	3.81 (2.94–4.27)	0.317
Triglycerides (mmol/L)	0.79 (0.60–1.19)	0.82 (0.49–1.29)	0.88 (0.64–1.56)	1.07 (0.63–1.30)	0.587
HDL (mmol/L)	1.11 (0.94–1.41)	1.03 (0.81–1.40)	1.13 (0.95–1.41)	1.10 (0.95–1.39)	0.634
ASSIGN cardiovascular risk score	17 (10–33)	12 (9–39)	24 (17–28)	16 (12–46)	0.285
Statin prescription	67/78 (85.9%)	20/25 (80.0%)	27/31 (87.1%)	12/17 (70.6%)	0.395
Aspirin prescription	67/78 (85.9%)	22/25 (88.0%)	29/31 (93.5%)	13/17 (76.5%)	0.429
P2Y12 inhibitor prescription	9/78 (11.5%)	3/25 (12.0%)	6/31 (19.4%)	3/17 (17.6%)	0.660
Anticoagulant prescription	3/78 (3.8%)	1/25 (4.0%)	2/31 (6.5%)	1/17 (5.9%)	0.832
Beta-blocker prescription	48/78 (61.5%)	15/25 (60.0%)	27/31 (87.1%)	11/17 (64.7%)	0.047
RAAS blocker prescription	34/78 (43.6%)	10/25 (40.0%)	14/31 (45.2%)	10/17 (58.8%)	0.664

Mann-Whitney U test or Fisher’s Exact test was applied according to variable types. All values are reported as median (IQR) or *n* (%).

CCB, calcium-channel blocker; CVA, cerebrovascular accident; MI, myocardial infarction; MVA, microvascular angina; PCI, percutaneous coronary intervention; RAASrenin-angiotensin–aldosterone systemVSA, vasospastic angina

### Global atherosclerotic plaque burden

The median number of SYNTAX coronary segments with ≥1% stenosis was 4 (IQR 2–7). The median number of proximal coronary segments (defined as: left main stem, proximal LAD, proximal circumflex or proximal RCA) with ≥1% luminal stenosis was 1 (IQR 1–3).

In this population without flow-limiting atherosclerosis, the median Gensini score was 6.0 (IQR 2.5–11.0). When divided according to coronary artery (excluding the left main stem), the median Gensini scores were 3.5 (IQR 1.5–5.0) in the LAD, 2.0 (IQR 0.0–3.5) in the left circumflex and 1.0 (IQR 0.0–2.0) in the RCA.

### Atherosclerotic plaque burden according to indices of microvascular function

The LAD underwent diagnostic guidewire testing in 85.4% (n=129), the circumflex in 5.3% (n=8) and RCA in 9.3% (n=14).

#### Indices of microvascular function

The invasive physiology results are summarised in table 2. In the absence of flow-limiting epicardial coronary disease, the CFR and IMR reflect vasoactive function, flow reserve and resistance of the microvascular bed. The CFR, IMR, MRR and RRR (all p<0.001), but not FFR (p=0.207), differed according to INOCA endotypes, reflecting the diagnostic criteria used. However, the CFR, MRR and RRR were higher and IMR lower in patients with isolated VSA, which was not significantly different from patients with non-cardiac symptoms (all p>0.22).

**Table 2:** Invasive physiology results from coronary function testing, grouped according to final clinical diagnosis

Physiology measurement	All participants (n=151)	Microvascular angina (MVA) (n=78)	Vasospastic angina (VSA) (n=25)	Mixed MVA and VSA (n=31)	Non-cardiac symptoms (n=17)	Kruskal-Wallis p value
FFR	0.88 (0.83–0.92)	0.89 (0.84–0.93)	0.85 (0.82–0.89)	0.86 (0.82–0.92)	0.88 (0.84–0.92)	0.207
MeanTmn Rest	0.78 (0.50–1.07)	0.82 (0.49–1.10)	0.67 (0.51–1.07)	0.78 (0.55–1.29)	0.75 (0.34–0.93)	0.852
MeanTmn Hyp	0.27 (0.20–0.39)	0.28 (0.21–0.42)	0.22 (0.17–0.27)	0.37 (0.29–0.50)	0.22 (0.15–0.29)	**<0.001**
CFR	2.6 (1.9–3.6)	2.3 (1.8–3.5)	3.2 (2.7–4.0)	2.0 (1.6–2.9)	3.2 (2.3–4.6)	**<0.001**
IMR	19 (14–29)	23 (15–32)	16 (13–20)	28 (18–37)	16 (11–20)	**<0.001**
MRR	3.3 (2.4–4.4)	3.1 (2.1–4.2)	4.0 (3.2–5.0)	2.7 (2.2–3.5)	4.3 (3.0–5.6)	**<0.001**
RRR	3.1 (2.3–4.1)	2.8 (2.1–4.0)	3.7 (3.2–5.0)	2.6 (2.1–3.3)	3.8 (2.8–4.7)	**0.002**

All values are reported as median (IQR).

CFR, coronary flow reserve; FFR, fractional flow reserve; IMR, Index of Microcirculatory Resistance; MeanTmn Hyp, mean transit time at hyperaemia; MeanTmn Restmean transit time at restMRR, microvascular resistance reserve; RRR, resistive reserve ratio

There were strong, positive monotonic correlations between CFR and MRR (r*_s_*=0.945, p<0.001), CFR and RRR (r*_s_*=0.940, p<0.001) and MRR and RRR (r*_s_*=0.985, p<0.001). Conversely, the relationships between CFR and IMR (p=0.364), MRR and IMR (p=0.134), and RRR and IMR (p=0.274) were not significant.

#### Plaque distribution and Gensini score

Abnormal CFR <2.0 (n=45; p=0.006), MRR ≤3.0 (n=70; p=0.028) and RRR <3.5 (n=85; p=0.044), but not IMR ≥25 (n=52; p=0.477) were associated with more diffuse atherosclerotic plaque distribution, defined by the aggregated number of SYNTAX coronary segments with luminal stenosis ≥1%.

Similarly, the univariable analysis revealed that abnormal CFR <2.0 (p=0.002), MRR ≤3.0 (p=0.025) and RRR <3.5 (p=0.043), but not IMR ≥25 (p=0.445) were associated with greater cumulative plaque burden, as quantified by the Gensini score.

#### Predictors of raised Gensini score

The factors associated with a raised Gensini score are summarised in table 3. Age, smoking history, hypertension, previous myocardial infarction and measured FFR (all p<0.04) were significant determinants of the Gensini score on univariable regression analysis. Abnormal CFR (p=0.012), MRR (p=0.026) and RRR (p=0.026), but not IMR, were each independently associated with higher Gensini scores. These effects persisted in multivariable models controlling for the potential confounders above (table 3). There was no evidence of multicollinearity in any analyses (all VIFs <1.2) and residual distributions were approximately normal.

**Table 3:** Covariables of atherosclerotic burden (Gensini score) as identified during linear regression analysis

Characteristic	Univariable		Multivariable					
	Spearman’s coefficient	P value	Correlation coefficient	P value	Correlation coefficient	P value	Correlation coefficient	P value
Age	0.290	**<0.001**	0.297	**<0.001**	0.308	**<0.001**	0.308	**<0.001**
Female Sex	−0.127	0.121	−0.152	**0.042**	−0.150	**0.048**	−0.148	**0.049**
Smoking history	0.166	**0.041**	0.012	0.871	0.016	0.838	0.013	0.868
Hypertension	0.191	**0.019**	0.071	0.344	0.070	0.358	0.073	0.338
Diabetes mellitus	0.115	0.161	0.063	0.402	0.075	0.325	0.073	0.336
Previous MI	0.252	**0.002**	0.214	**0.005**	0.213	**0.006**	0.213	**0.006**
FFR	−0.208	**0.011**	−0.240	**0.002**	−0.257	**<0.001**	−0.256	**<0.001**
CFR	−0.251	**0.002**	−0.191	**0.012**				
IMR	0.063	0.445						
MRR	−0.183	**0.025**					−0.169	**0.026**
RRR	−0.167	**0.043**			−0.170	**0.026**		

CFR, coronary flow reserve; FFR, fractional flow reserve; IMR, Index of Microcirculatory Resistance; MI, myocardial infarction; MRR, microvascular resistance reserve; RRR, resistive reserve ratio

### Atherosclerotic plaque burden according to INOCA endotype

In total, 55.0% (n=83) of individuals had abnormal CFR and/or IMR. When considering acetylcholine provocation results, 72.2% (n=109/151) met the criteria for MVA and 37.1% (n=56/151) had features of epicardial VSA. The final diagnosis was MVA in 51.7% (n=78), VSA in 16.6% (n=25), mixed microvascular and VSA in 20.5% (n=31) and reclassification to non-cardiac symptoms in 11.3% (n=17).

Participants with coronary microvascular dysfunction as defined by an abnormal CFR <2.0 and/or IMR ≥25 (n=83) had a higher number of diseased coronary segments versus those with normal CFR and IMR (median 5 (IQR 2–7) vs 4^1^,^6^ ; p=0.039), table 4. Accordingly, the cumulative atherosclerotic plaque burden as assessed by the Gensini score was also higher in participants with versus without microvascular dysfunction by the same criteria (median 7.5 (IQR 3.5–11.5) vs 5.3 (2.5–10.0); p=0.028).

**Table 4:** Atherosclerotic distribution and plaque burden when stratified according to final clinical diagnosis and diagnostic guidewire result

Characteristic	MVA (n=78)	VSA (n=25)	Mixed MVA and VSA(n=31)	Non-cardiac symptoms(n=17)	P value
Number of diseased coronary segments	4 (2–7)	4 (1–6)	6 (3–8)	3 (1–5)	**0.032**
Number of diseased proximal segments	1 (1–3)	1 (1–2)	2 (1–2)	1 (0–2)	**0.020**
	**Abnormal CFR/IMR (n=83**)		**Normal CFR/IMR (n=68**)		
Number of diseased coronary segments	5 (2–7)		4 (1–6)		**0.039**
Number of diseased proximal segments	2 (1–3)		1 (0–2)		**0.022**
Gensini score	7.5 (3.5–11.5)		5.5 (2.5–10.0)		**0.028**

CFR <2.0 or IMR ≥25 is considered abnormal. INOCA endotypes were defined according to the COVADIS criteria. The proximal coronary segments are the left main stem (LMS), proximal left anterior descending (LAD), proximal left circumflex (LCx) or proximal right coronary artery (RCA). All values are reported as median (IQR).

CFR, coronary flow reserve; COVADISCoronary Vasomotion Disorders International Study GroupIMRIndex of Microcirculatory ResistanceINOCAischaemia with no obstructive coronary artery diseaseMVA, microvascular angina; VSA, vasospastic angina

There was a significant difference in the total number of diseased segments when comparing participants with different INOCA endotypes: MVA (median four segments (IQR 2–7)), VSA (4 (1–6)), mixed microvascular and VSA (6 (3–8)) and non-cardiac symptoms (3 (1–5)); p=0.032 (table 4).

**Figure 1:**
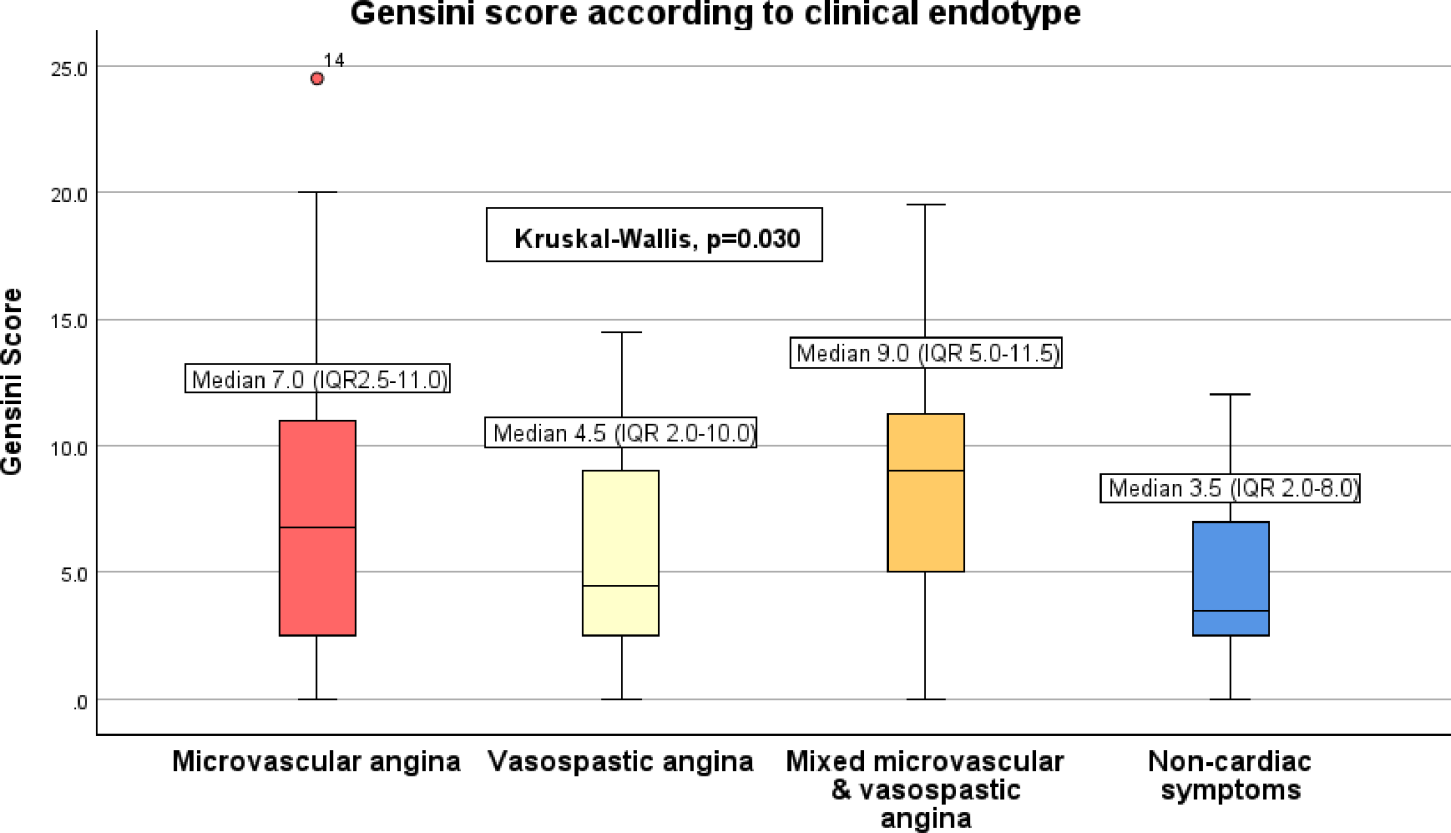
Cumulative atherosclerotic burden as defined by the Gensini score, stratified according to clinical endotype. Diagnoses were made based on combined invasive diagnostic guidewire and pharmacological provocation testing. Impaired coronary flow reserve (CFR <2.0) or elevated index of microcirculatory resistance (IMR ≥25) are indicative of coronary microvascular dysfunction. Coronary microvascular dysfunction is associated with a higher Gensini score. CFR, coronary flow reserve; IMR, Index of Microcirculatory Resistance.

Cumulative plaque burden as reflected by the Gensini score also differed by INOCA endotype: MVA (median Gensini 7.0 (2.5–11.0)), VSA (4.5 (2.0–10.0)), mixed microvascular and VSA (9.0 (5.0–11.5)) and non-cardiac symptoms (3.5 (2.0–8.0)); Kruskal-Wallis p=0.030 (figure 1).

### Discussion

We evaluated coronary atherosclerotic plaque burden and microvascular function simultaneously in a prospectively recruited cohort of participants with suspected INOCA. We identified novel associations between indices of microvascular function (CFR, MRR and RRR) and the global burden of coronary atherosclerosis, as quantified by the Gensini score. Our findings address a knowledge gap in INOCA; individuals with coronary microvascular dysfunction, particularly those with dual pathologies (mixed MVA and VSA) have substantially greater plaque burden compared with those with non-cardiac symptoms.

Our study has several novel findings. First, we provide a systematic global plaque assessment of all coronary segments in the SYNTAX classification using the Gensini score, a validated measure of coronary atherosclerosis burden. Second, we explored the associations between plaque burden and novel markers of microvascular function (MRR, RRR). Third, our cohort participants underwent comprehensive assessment with both bolus thermodilution-based indices, as well as intracoronary acetylcholine provocation testing. This allowed for stratification of participants by specific mechanisms of vasomotor dysfunction and differentiates our study from prior research.^9 21^

AlBadri *et al*^21^ previously reported increased coronary plaque burden assessed using single coronary artery intravascular ultrasound in patients with microvascular dysfunction, as identified using a Doppler diagnostic guidewire. While our study agrees with their finding of increased plaque burden in patients with versus without coronary microvascular dysfunction, they found an association with the Doppler-derived hyperaemic resistance ratio (HMR), which is conceptually similar, but distinct from IMR. The differences in our results may be explained by the differing methodology for plaque assessment (global analysis from angiography vs single-vessel intravascular imaging) and measured indices (IMR vs HMR). Other published works demonstrated strong links between reduced CFR and MRR with adverse cardiovascular outcomes.^1 4 5 22^ However, the underlying mechanisms remain incompletely understood.^23^

We describe correlations between CFR, MRR, RRR and the Gensini score. The higher Gensini scores in participants with MVA are driven by: a greater number of diseased coronary segments, reflecting more diffuse disease; more severe luminal stenosis, indicating higher plaque burden per segment; and more frequent involvement of proximal coronary arteries. Our findings provide a mechanistic link between these indices and the higher rates of acute coronary syndromes seen with INOCA, via increased coronary plaque burden.^23^

In the absence of flow-limiting epicardial coronary artery disease, an abnormal CFR reflects abnormalities in the coronary microcirculation,^24^ supporting a diagnosis of MVA. Our findings provide objective, quantitative evidence of an association between coronary microvascular dysfunction and diffuse atherosclerosis in a population with INOCA.

Importantly, reduced CFR, MRR and RRR remained independently associated with higher Gensini scores after controlling for potential confounders. Our multivariable modelling controlled for FFR, the reference physiological index for assessment of epicardial disease. Furthermore, while CFR is influenced by epicardial stenosis, indices specific to microvascular function like MRR and RRR were also independently associated with a raised Gensini score.

Strong correlations were observed between CFR, MRR and RRR. However, IMR was not associated with cumulative plaque burden. Since IMR may be a surrogate measure for microvascular structural remodelling in chronic coronary syndromes, the lack of association between IMR and atherosclerosis may reflect distinct vascular pathophysiology.

#### Clinical implications

Our findings have implications for patient care. Treatments that stabilise plaques and potentiate regression, importantly statins, may reduce cardiovascular risk in INOCA, particularly in patients with impaired CFR and/or MRR. MVA associated with cardiovascular risk factors that mediate epicardial atherosclerosis.^25 26^ Similarly, VSA is linked to smoking history.^27^ These associations support the hypothesis that microvascular dysfunction and epicardial coronary artery disease have a shared pathophysiological basis. Ultimately, our findings reinforce the importance of antiatherosclerosis therapies in INOCA. The potential prognostic benefits of intensive control of cardiovascular risk factors are currently being assessed in the WARRIOR trial.^28^

### Study limitations

Atherosclerotic changes may only be evident on invasive coronary angiography after plaque burden within the vessel wall exceeds >40% by cross-sectional area.^29^ This in turn may reduce the sensitivity of the Gensini score. Invasive angiography remains the reference standard for assessing coronary luminal stenoses. The angiographic views (>30 degrees projection apart, ideally orthogonal) reflect standard clinical practice, still allowing for assessment of eccentric plaque.

Our study did not employ intravascular imaging. While standard angiographic analysis is less sensitive for detecting atherosclerotic plaque, it remains highly specific^30^ and has identified novel associations. Future studies could incorporate multi-vessel assessment with intracoronary imaging (intravascular ultrasound or optical coherence tomography). CT coronary angiography could be an alternative, but its spatial resolution limits the analysis of distal coronary branches. Furthermore, CT does not provide a measure of vasomotor function. In our study, coronary plaque burden and microvascular function assessments were acquired simultaneously.

The participants in the CorMicA study were selected, undergoing clinically indicated invasive investigation.^14^ The true incidence of microvascular dysfunction and plaque burden in the general population remain ill defined. Our study had a cross-sectional design and future cohort studies should clarify longer-term prognostic implications of atherosclerosis in INOCA.

## Conclusions

We provided novel insights into relations between global atherosclerotic plaque burden defined in all SYNTAX coronary segments using the Gensini score, and INOCA endotypes as stratified according to comprehensive invasive coronary function testing.

Abnormal CFR, MRR and RRR, and MVA were associated with increased atherosclerotic plaque burden, evidenced by higher Gensini scores. These novel findings provide a mechanistic link between INOCA and cardiovascular events, reinforcing the importance of anti-atherosclerosis therapy in patients with MVA.

## supplementary material

10.1136/heartjnl-2024-324677 online supplemental file 1

## Data Availability

Data are available upon reasonable request.

## References

[1] Kunadian V, Chieffo A, Camici PG (2020). An EAPCI Expert Consensus Document on Ischaemia with Non-Obstructive Coronary Arteries in Collaboration with European Society of Cardiology Working Group on Coronary Pathophysiology & Microcirculation Endorsed by Coronary Vasomotor Disorders International Study Group. Eur Heart J.

[2] Ong P, Camici PG, Beltrame JF (2018). International standardization of diagnostic criteria for microvascular angina. Int J Cardiol.

[3] Beltrame JF, Crea F, Kaski JC (2017). International standardization of diagnostic criteria for vasospastic angina. Eur Heart J.

[4] Boerhout CKM, Lee JM, de Waard GA (2023). Microvascular resistance reserve: diagnostic and prognostic performance in the ILIAS registry. Eur Heart J.

[5] Lee SH, Choi KH, Hong D (2024). Prognostic Implications of Microvascular Resistance Reserve in Symptomatic Patients With Intermediate Coronary Stenosis. JACC: Cardiovascular Interventions.

[6] Pepine CJ, Anderson RD, Sharaf BL (2010). Coronary microvascular reactivity to adenosine predicts adverse outcome in women evaluated for suspected ischemia results from the National Heart, Lung and Blood Institute WISE (Women’s Ischemia Syndrome Evaluation) study. J Am Coll Cardiol.

[7] Vrints C, Andreotti F, Koskinas KC (2024). 2024 ESC Guidelines for the management of chronic coronary syndromes. Eur Heart J.

[8] Ford TJ, Corcoran D, Oldroyd KG (2018). Rationale and design of the British Heart Foundation (BHF) Coronary Microvascular Angina (CorMicA) stratified medicine clinical trial. Am Heart J.

[9] Echavarria-Pinto M, Escaned J, Macías E (2013). Disturbed coronary hemodynamics in vessels with intermediate stenoses evaluated with fractional flow reserve: a combined analysis of epicardial and microcirculatory involvement in ischemic heart disease. Circulation.

[10] de Vos A, Jansen TPJ, van ’t Veer M (2023). Microvascular Resistance Reserve to Assess Microvascular Dysfunction in ANOCA Patients. JACC Cardiovasc Interv.

[11] De Bruyne B, Pijls NHJ, Gallinoro E (2021). Microvascular Resistance Reserve for Assessment of Coronary Microvascular Function: JACC Technology Corner. J Am Coll Cardiol.

[12] Scarsini R, De Maria GL, Borlotti A (2019). Incremental Value of Coronary Microcirculation Resistive Reserve Ratio in Predicting the Extent of Myocardial Infarction in Patients with STEMI. Insights from the Oxford Acute Myocardial Infarction (OxAMI) Study. Cardiovasc Revasc Med.

[13] Layland J, Carrick D, McEntegart M (2013). Vasodilatory capacity of the coronary microcirculation is preserved in selected patients with non-ST-segment-elevation myocardial infarction. Circ Cardiovasc Interv.

[14] Ford TJ, Stanley B, Good R (2018). Stratified Medical Therapy Using Invasive Coronary Function Testing in Angina: The CorMicA Trial. J Am Coll Cardiol.

[15] Ford TJ, Stanley B, Sidik N (2020). 1-Year Outcomes of Angina Management Guided by Invasive Coronary Function Testing (CorMicA). JACC Cardiovasc Interv.

[16] Berry C, L’Allier PL, Grégoire J (2007). Comparison of intravascular ultrasound and quantitative coronary angiography for the assessment of coronary artery disease progression. Circulation.

[17] Kerolus MG, Joshi KC, Johnson AK (2020). Co-registration of Intravascular Ultrasound With Angiographic Imaging for Carotid Artery Disease. World Neurosurg.

[18] Sianos G, Morel M-A, Kappetein AP (2005). The SYNTAX Score: an angiographic tool grading the complexity of coronary artery disease. EuroIntervention.

[19] Gensini GG (1983). A more meaningful scoring system for determining the severity of coronary heart disease. Am J Cardiol.

[20] Rampidis GP, Benetos G, Benz DC (2019). A guide for Gensini Score calculation. Atherosclerosis.

[21] AlBadri A, Eshtehardi P, Hung OY (2019). Coronary Microvascular Dysfunction Is Associated With Significant Plaque Burden and Diffuse Epicardial Atherosclerotic Disease. JACC Cardiovasc Interv.

[22] Lee JM, Jung J-H, Hwang D (2016). Coronary Flow Reserve and Microcirculatory Resistance in Patients With Intermediate Coronary Stenosis. J Am Coll Cardiol.

[23] Mortensen MB, Dzaye O, Steffensen FH (2020). Impact of Plaque Burden Versus Stenosis on Ischemic Events in Patients With Coronary Atherosclerosis. J Am Coll Cardiol.

[24] Rahman H, Corcoran D, Aetesam-Ur-Rahman M (2019). Diagnosis of patients with angina and non-obstructive coronary disease in the catheter laboratory. Heart.

[25] Hansen B, Holtzman JN, Juszczynski C (2023). Ischemia with No Obstructive Arteries (INOCA): A Review of the Prevalence, Diagnosis and Management. Curr Probl Cardiol.

[26] Eroglu S, Sade LE, Bozbas H (2009). Association of serum adiponectin levels and coronary flow reserve in women with normal coronary angiography. Eur J Cardiovasc Prev Rehabil.

[27] Tran MV, Marceau E, Lee P-Y (2021). The Smoking Paradox: A Twist in the Tale of Vasospastic Angina. *J Vasc Med Surg*.

[28] Handberg EM, Merz CNB, Cooper-Dehoff RM (2021). Rationale and design of the Women’s Ischemia Trial to Reduce Events in Nonobstructive CAD (WARRIOR) trial. Am Heart J.

[29] Ford TJ, Berry C, De Bruyne B (2017). Physiological Predictors of Acute Coronary Syndromes: Emerging Insights From the Plaque to the Vulnerable Patient. JACC Cardiovasc Interv.

[30] Topol EJ, Nissen SE (1995). Our preoccupation with coronary luminology. The dissociation between clinical and angiographic findings in ischemic heart disease. Circulation.

