# Supplementary material for: Coronary microvascular function and atherosclerotic plaque burden in ischaemia and no obstructive coronary arteries: a secondary analysis of the CorMicA trial
Source: Heart. 2024 Nov 27;111(3):e324677. doi: 10.1136/heartjnl-2024-324677 (PMC11874308; doi:10.1136/heartjnl-2024-324677)
Supplement: online supplemental file 1 [file heartjnl-111-3-s001.pdf]

COROnary MICrovascular Angina (CorMicA): a randomised, controlled, pilot trial

Running title:	CorMicA
Protocol Version:	1.7
Date:	23/11/2017
REC Reference Number:	16/WS/0192
IRAS project ID:	202330
Sponsor's Protocol Number:	16/CARD/25

Sponsor: Golden Jubilee National Hospital

Funder: British Heart Foundation, NHS Cardiology Endowments.

This study will be performed according to the Research Governance Framework for Health and Community Care (Second edition, 2006) and WORLD MEDICAL ASSOCIATION DECLARATION OF HELSINKI Ethical Principles for Medical Research Involving Human Subjects 1964 (as amended).

CONTACTS

Chief Investigator

Professor Colin Berry

Honorary Consultant Physician and Cardiologist

Institute of Cardiovascular and Medical Sciences

BHF Glasgow Cardiovascular Research Centre

126 University Place

University of Glasgow

Glasgow, G12 8TA

Tel: 0141 330 1671

Fax: 0141 330 7335

E-mail: colin.berry@glasgow.ac.uk

Funder

British Heart Foundation

BHF Clinical Research Training Fellowship, FS/14/15-30661

British Heart Foundation Centre of Research Excellence Award

PROTOCOL APPROVAL

COROnary MICrovascular Angina (CorMicA): a randomised, controlled proof-of-concept pilot trial

Chief Investigator

Professor Colin Berry BSc, PhD, FRCP, FACC, FESC

Honorary Consultant Physician and Cardiologist

Institute of Cardiovascular and Medical Sciences

BHF Glasgow Cardiovascular Research Centre

126 University Place

University of Glasgow

Glasgow, G12 8TA

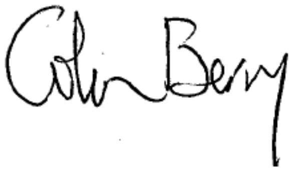

Signature:

Date: 23.11.2017

Table of contents

CONTACTS 2

TABLE OF CONTENTS 4

STUDY SYNOPSIS 11

FIGURE 1 21

SCHEDULE OF ASSESSMENTS 23

1. INTRODUCTION 36

2. PILOT STUDY OBJECTIVES 52

3. STUDY DESIGN 60

4. MONITORING OF ADVERSE EVENTS 96

5. STATISTICS AND DATA ANALYSIS PLAN 98

6. STUDY CLOSURE / DEFINITION OF END OF STUDY 104

7. DATA HANDLING 104

8. STUDY MANAGEMENT 105

9. STUDY MONITORING AND AUDITING 106

10. PROTOCOL AMENDMENTS 106

11. ETHICAL CONSIDERATIONS 106

12. INSURANCE AND INDEMNITY ... 107

13. FUNDING, PEER REVIEW AND PUBLIC INVOLVEMENT ... 108

15. DISSEMINATION OF FINDINGS ... 109

16. REFERENCES ... 110

APPENDIX 1. CORMICA SCREENING LOG ... 131

APPENDIX 2. CARDIOLOGIST QUESTIONNAIRE FOR USE DURING THE INDEX ADMISSION 133

APPENDIX 3. CORONARY FUNCTION TESTING WITH ACETYLCHOLINE (ACH) ... 137

APPENDIX 4. DEFINITIONS OF CORONARY FUNCTION TEST OUTCOMES. ... 140

APPENDIX 5. DISCHARGE GUIDANCE FRAMEWORK FOR GPS ACCORDING TO DIAGNOSIS. ... 143

<INSERT GJNH LETTERHEAD> ... 143

DIAGNOSIS – MICROVASCULAR ANGINA. ... 143

GUIDELINE RECOMMENDED THERAPY ... 143

PHARMACOLOGICAL MANAGEMENT ... 143

NON PHARMACOLOGICAL LIFESTYLE & RISK FACTOR CONTROL. ... 143

APPENDIX 5. DISCHARGE GUIDANCE FRAMEWORK FOR GPS ACCORDING TO DIAGNOSIS. ... 145

<INSERT GJNH LETTERHEAD> ... 145

GUIDELINE RECOMMENDED THERAPY ... 145

DIAGNOSIS – VASOSPASTIC ANGINA 145

PHARMACOLOGICAL MANAGEMENT 145

NON PHARMACOLOGICAL LIFESTYLE & RISK FACTOR CONTROL.................................... 145

APPENDIX 6. ANXIETY & DEPRESSION (PATIENT HEALTH QUESTIONNAIRE 4) - PHQ4 147

APPENDIX 7. ILLNESS PERCEPTION (BRIEF ILLNESS PERCEPTION QUESTIONNAIRE) – B-IPQ 148

APPENDIX 8. TREATMENT SATISFACTION (TREATMENT SATISFACTION QUESTIONNAIRE) – TSQM 9.. 149

APPENDIX 9. COVER LETTER FOR FOLLOW UP QUESTIONNAIRE – CORMICA STUDY........ 153

<INSERT GJNH LETTERHEAD>.. 153

INFORMATION ON URINE COLLECTION 154

APPENDIX 12. COVER LETTER FOR 12 MONTH QUESTIONNAIRE – CORMICA STUDY 159

Golden Jubilee National Hospital

NHS National Waiting Times Centre

Cover Letter for Follow up Questionnaire – CorMicA study

"A study of whether tests & treatment of coronary function improve well-being of patients with angina."

"**CORonary MICrovascular Angina (CorMicA): a randomised, controlled, pilot trial.**"

Dear Sir or Madam,

We would be most grateful if you could complete the attached questionnaire. Please bring this questionnaire with you to the 12-month follow up appointment at the Golden Jubilee National Hospital. This questionnaire is a vital part of our research and we take this opportunity to thank you once again for your assistance in completing the questions. If you are unable to attend please kindly return the completed questionnaire via the address below. Our research project is funded by the British Heart Foundation.

Additionally, please list the medications that you take on a daily basis in the space below, or alternatively, provide a copy of your current prescription.

Please write **today's date - ………………………………..**

Medicine	Dose	Medicine	Dose

We are most grateful for your participation in this study. If you have any questions or concerns relating to the study please do get in touch with me on the details below.

Kind regards,

Dr Tom Ford (Clinical research fellow for Prof Colin Berry)

CorMicA Returns, Department of Cardiology, Golden Jubilee National Hospital, Tel: 0141 951 5180.

CorMicA Cover letter 12 month follow up V1.0, 23/11/17 Randomisation ID ……………………

APPENDIX 13. COVER LETTER FOR REGISTRY CORMICA FOLLOW-UP............................. 159

ABBREVIATIONS

ACh	Acetylcholine
AE	Adverse event
BHF	British Heart Foundation
BMI	Body Mass Index
CAD	Coronary artery disease
CMR	Cardiac magnetic resonance imaging
CFR	Coronary flow reserve
CMA	Coronary microvascular angina
CMD	Coronary microvascular disease
CRF	Case report form
ECG	Electrocardiogram
eGFR	Estimated Glomerular Filtration Rate
ECE	Endothelin converting enzyme
ET-1	Endothelin-1
ET-RA	Endothelin receptor antagonist
FFR	Fractional flow reserve
GJNH	Golden Jubilee National Hospital
GP	General Practitioner
GTN	Glyceryl trinitrate
HCRU	Health Care Resource Use
HLA	Horizontal long axis
IMR	Index of microvascular resistance
IC	Intracoronary
ICH GCP	International Conference on Harmonization of Good Clinical Practice
LV	Left ventricle

LVH	Left ventricular hypertrophy
LVEF	Left ventricular ejection fraction
LVEDVI	Left ventricular end-diastolic volume index
LVESVI	Left ventricular end-systolic volume index
MHRA	Medicines and Healthcare products Regulatory Agency
MI	Myocardial infarction
MRC	Medical Research Council
MRI	Magnetic resonance imaging
MVO	Microvascular obstruction
MPRI	Myocardial perfusion reserve index
NEP	Neutral endopeptidase
NHS	National Health Service
NICE	National Institute for Health and Care Excellence
NT-proBNP	N-terminal pro-brain natriuretic peptide (NT-proBNP)
PAH	Pulmonary arterial hypertension
PCI	Percutaneous coronary intervention
REC	Research Ethics Committee
RRR	Resistive reserve ratio
SAE	Serious adverse event
SA	Short Axis
SOP	Standard Operating Procedure
SSS	Summed stress score
SUSAR	Suspected Unexpected Serious Adverse Reaction
TIA	Transient ischaemic attack
TMG	Trial Management Group

TIMI	Thrombolysis In Myocardial Infarction
RSC	Registry steering committee
UK	United Kingdom
ULN	Upper limit of normal
VLA	Vertical long axis
WP	Work package

STUDY SYNOPSIS

Title of Study:	CORonary MICrovascular Angina (CorMicA): a randomised, controlled proof-of-concept pilot trial of routine adjunctive functional testing during invasive diagnostic coronary angiography vs. standard care with invasive coronary angiography only.
Duration of Study:	Active phase 3 years; long term 20 years.
Primary Objective:	To assess whether a diagnostic strategy involving invasive tests of coronary function is routinely feasible, safe, impactful on diagnosis and management, and provides preliminary information on clinical outcomes.
Secondary Objective:	To assess feasibility including:<br>- Recruitment rates,<br>- Loss to follow up,<br>- Completion of the diagnostic protocol,<br>- Integrity of blinding in the catheter laboratory,<br>- Cross-over between groups,<br>- Adherence with medication during follow-up,<br>To assess safety of the tests of coronary function,

	To assess impact of disclosure of the coronary function test results on the diagnosis and certainty of the diagnosis (diagnostic utility),<br><br>To assess impact of disclosure of the coronary function test results on clinical management (including treatment and investigations)<br><br>To assess the relationships between cardiovascular risk factors, reflected by validated risk scores (e.g. ASSIGN, JBS3), and parameters of coronary function.<br><br>Health status: Rose angina, Seattle angina scores (4 components), EQ-5D health status, Illness perception (Brief IPQ), anxiety/depression (PHQ4), treatment satisfaction (TSQM)<br>Functional status and activity levels: Duke activity status index (DASI) and International Physical Activity Questionnaire (IPAQ-SF) short-form.<br><br>N-terminal prohormone brain natriuretic peptide (NT-proBNP), a surrogate outcomes of prognosis,<br><br>Changes in lifestyle associated with coronary function testing (smoking, diet, exercise, weight).<br><br>Small blood vessel density in the tongue.<br><br>Sub-study (optional): Myocardial perfusion by

	quantitative stress CMR.<br><br>Sub-study (optional): Small blood vessel function in vitro<br><br>NHS healthcare resource utilization, including primary and secondary care costs for tests, procedures and out-patient visits, and medicines.<br><br>To undertake an exploratory health economic analysis
Primary Endpoint:	The within-subject change at 6 months from baseline for the domains of the Seattle Angina Questionnaire.
Rationale:	The health burden related to known or suspected coronary heart disease remains persistently high. Each year, there are more than 20,000 new cases of angina, and ~240,000 invasive coronary angiograms performed. However, obstructive coronary artery disease (CAD) is only detected in ~40-50% of these procedures. Despite the apparently reassuring findings, patients with a 'negative' angiogram have an increased long term risk of cardiovascular events. Although the factors associated with a low-yield from invasive angiography are multifactorial, coronary microvascular angina (CMA) may occur in ~1/3 of patients with a negative' angiogram.<br><br>There is a critical missing link between the use of relevant diagnostic tests (invasive tests of coronary artery function and coronary microvascular disease; non-

	invasive stress perfusion MRI), drugs with known efficacy (e.g. verapamil), medical decisions for the use of these tests and treatments, and prognosis. This conundrum is illustrated in the NICE-95 guidelines for Chest Pain 'in people with "typical angina-like chest pain if investigation excludes flow-limiting disease in the epicardial coronary arteries", then 'Syndrome X' should be considered', but no other guidance is provided to clinicians. There is an absence of evidence from randomised trials that a diagnostic strategy which is linked to therapy improves the wellbeing of patients with angina without obstructive CAD (to rule-in or rule-out CMA). This knowledge gap results in these adjunctive tests and related treatments scarcely if ever used in routine NHS care<br><br>This proposal aims firstly to address the gap between diagnostic tests and treatments through a randomized controlled strategy trial, and secondly to include mechanistic studies to better understand the pathophysiology of this condition for the development of novel therapies in well characterised patients with CMA and matched controls.
Methodology:	CorMicA is a prospective, double blind, 1:1 randomized, controlled, parallel group, internal pilot clinical trial. The design involves enrolling participants with stable angina who are undergoing invasive coronary angiography

	which is standard of care. The sub-group of participants who have obstructive coronary disease excluded by angiography ± fractional flow reserve (FFR) would be eligible to take part whereas the sub-group with obstructive disease would be excluded. The strategy involves ad hoc adjunctive testing of coronary function (disclosed) vs. standard care with invasive angiography only (coronary function measured but not disclosed). Coronary function tests are not standard of care and are very rarely undertaken in clinical practice (see pre-pilot data). The rationale for our study reflects the lack of any evidence that treatment associated with these tests improves prognosis. Blinding of the cardiologist and patient to the test results can be justified to enable optimal standard care in the control group, and minimise bias. This design aligns with other recent studies by our group, such as the FAMOUS-NSTEMI trial (ClinicalTrials.gov Identifier: NCT01764334).<br><br>The MHRA have classed CorMicA as a non-CTIMP.<br><br>The design represents a multi-centre pilot study in 150 subjects to gather information on feasibility, safety and preliminary evidence of efficacy to optimise the implementation of a future larger trial adopting a similar design. The current study is an internal pilot trial.

	Longer-term follow-up for health outcomes will be undertaken using electronic record linkage omitting the need for participants to undergo further research visits.<br><br>The pilot is described as 'internal' reflecting the NIHR definition whereby the study serves as a rehearsal for a larger definitive trial. The data from the pilot trial will contribute to that trial's final dataset.
Sample Size:	150 randomised subjects.
Screening	All patients undergoing elective invasive coronary angiography with symptoms of angina or angina equivalent will be screened on the ward before the procedure
Registration:	Administered via the Robertson Centre for Biostatistics
Main Inclusion Criteria:	Age ≥18 years<br><br>A clinically-indicated plan for invasive coronary angiography<br><br>Symptoms of angina or angina-equivalent (according to the Rose- and Seattle Angina questionnaires).
Main Exclusion Criteria:	A non-coronary indication for invasive angiography e.g. valve disease,

	During the angiogram: obstructive disease evident in a main coronary artery (diameter >2.5 mm), i.e. a coronary stenosis>50% or a fractional flow reserve (FFR) ≤0.80<br><br>Lack of informed consent.<br><br>The optional stress MRI sub-study has the following exclusion criterion:<br><br>Contra-indication to contrast-enhanced CMR e.g. severe renal dysfunction (GFR < 30 ml/min), a non-CMR compatible pacemaker or defibrillator, or asthma with routine inhaler use.<br><br>The optional vascular sub-study has the following exclusion criterion:<br><br>Oral anti-coagulation<br><br>The exclusion criteria for the sub-studies do not apply to the main study

Study assessments:	Coronary artery disease as revealed by invasive coronary angiography.<br><br>Coronary microvascular function assessed invasively with a diagnostic coronary guidewire using pressure and thermodilution for coronary flow reserve (CFR), index of microvascular resistance (IMR), and the resistive reserve ratio (RRR).<br><br>Coronary endothelial function assessed invasively using intra-coronary infusion of acetylcholine (ACh, a muscarinic receptor agonist (M2)) administered at incremental concentrations (10^{-6}M, 10^{-5}M, 10^{-4}M; 2 min each) via the guiding or infusion catheter.<br><br>Provocative spasm challenge with high rate infusion 100mcg of 10^{-4}M ACh (5.5ml given over 20 seconds).<br><br>Coronary endothelial-independent function following an IC bolus of 300 µg (3 ml) of GTN<br><br>The laboratory analyses will include endothelial cytokines (ET-1, ICAM, VCAM, p-selectin, IL-6), hsCRP, metabolic status (plasma glucose, $HbA_{1C,}$ lipids), metabolomics, hs-troponin, hsCRP, NT-proBNP, plasma serotonin. The buffy coat will be stored for DNA. Blood samples will be saved for future analyses of interest. NT-proBNP will represent a surrogate outcome for prognosis. Residual

	blood will be saved for future analyses of interest.<br><br>Sub-lingual dark field videomicroscopy (Microvision) for small vessel density and distribution will be assessed on the ward<br><br>Post coronary angiography participants will be invited to undergo the following 2 assessments:<br><br>- Small arteriolar vasoactive function assessed in isolated resistance arteries obtained by gluteal skin biopsy.<br><br>- Cardiovascular magnetic resonance imaging<br><br>Health status questionnaire (Rose Angina, Seattle angina, EQ5D-5L) at baseline and during follow-up.<br><br>Illness perception (Brief IPQ), anxiety/depression (PHQ4) , treatment satisfaction (TSQM) at baseline and during follow up<br><br>Changes in lifestyle associated with coronary function testing (smoking, diet, exercise, weight).<br><br>Functional status and activity levels: Duke activity status index questionnaire (DASI) and International Physical Activity Questionnaire (IPAQ-SF) short-form.<br><br>Pharmaceutical therapy at baseline, 6 weeks, 6 months, 12, 18 and 24 months.

	Urine testing at baseline, 6, 12, 18 and 24 months for metabolites of vasoactive drugs to assess mechanisms and compliance of 'non-responders'<br><br>NHS resource utilisation<br><br>Health outcomes from review of electronic records from 6 months including death, cardiovascular hospitalisation and coronary revascularisation
Duration of Follow-up:	Minimum of 6 months with pre-specified timeline events up to 24 months, including a visit in person at 1 year. Contact by telephone or post at other times.
Statistical Analysis	The Robertson Centre for Biostatistics will manage and analyse trial data. All statistical analyses will be conducted according to the Statistical Analysis Plan, which will be authored by the Trial Statistician. Long-term follow-up analysis is intended if additional funding is available.

Figure 1.

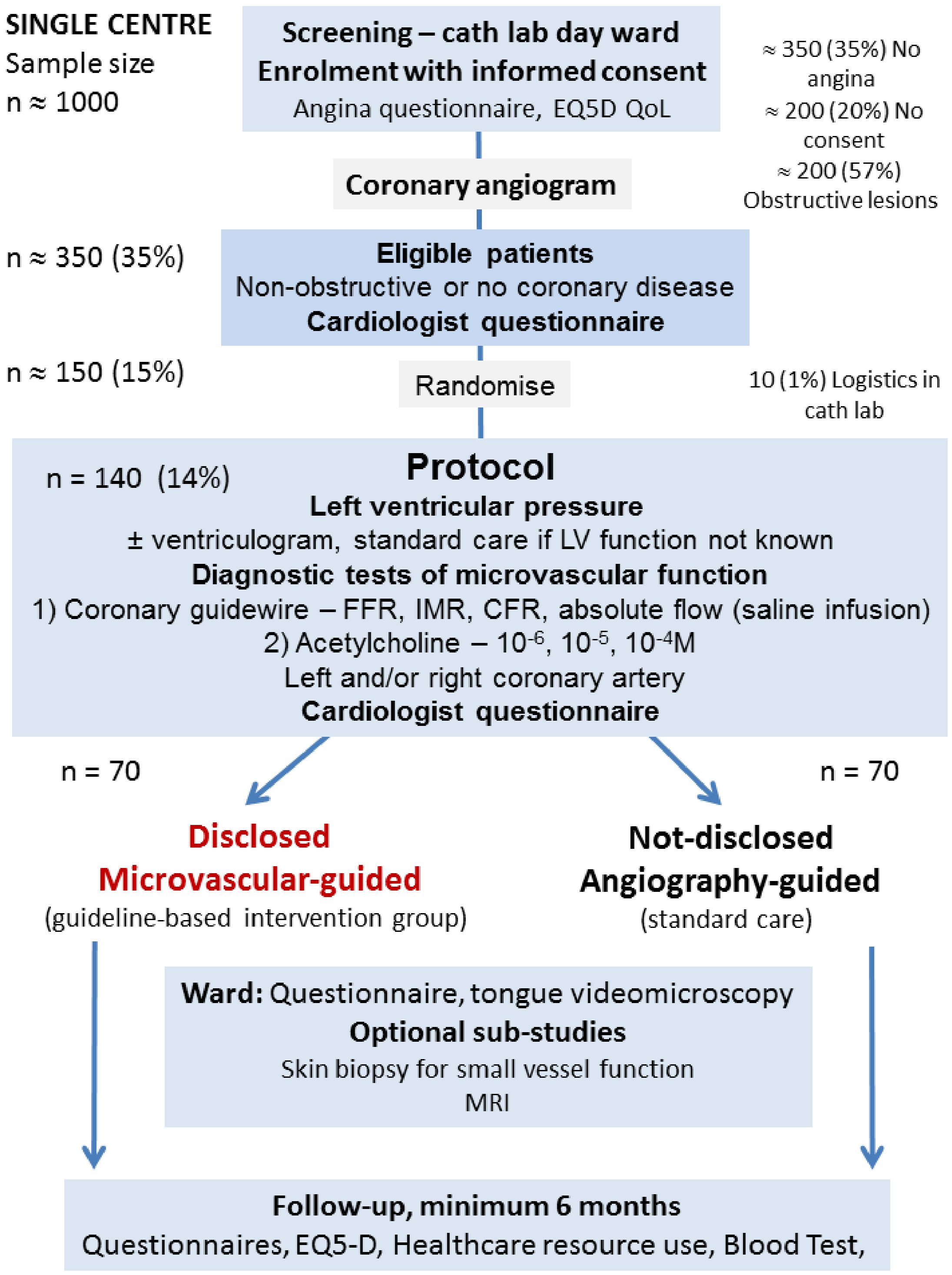

Footnote: We anticipate that at least 1000 elective admissions for invasive angiography may need to be screened in order that 150 will be randomised over 12 months. Our projected number of participants is as follows: of 1000 screened

patients, 350 may be deemed to have non-anginal symptoms based on validated questionnaires before the angiogram procedure. Of the remaining 650 patients, 200 may not give consent despite being potentially eligible and 100 may be eligible for the main study, but not for MRI. Of approximately 350 patients who have given informed consent to participate, 180 will have non-obstructive coronary disease, or no angiographic evidence of disease. Of these, 30 may not be randomised for logistical reasons or consultant/patient preference. Finally, 150 subjects will be randomised.

SCHEDULE OF ASSESSMENTS

Study timeline	Baseline	Day 0	Day 1 – 6 weeks	6 months	12, 24 months (or close out visit if <24 months)	> 2 years
Assessment	Cardiology ward	Catheter lab.	MRI	Outpatient	Outpatient	E-linkage
		Research	Research	Research	Research	Research
Screening: Inclusion / Exclusion Criteria	√ (NHS Support)					
Health status questionnaires inc lifestyle	√ (Research)			√ (R)	√ (R)	
Duke activity status index (DASI) and International	√ (Research)				√ (R)	

Study timeline	Baseline	Day 0	Day 1 – 6 weeks	6 months	12, 24 months (or close out visit if <24 months)	> 2 years
Physical Activity Questionnaire (IPAQ-SF) short-form.						
Written informed consent[1]	√ (NHS Support)					
Vital sign observation (heart rate, rhythm, blood pressure)	√ (Standard care)					
Electrocardiogram		√ (Standard care)				
Physical Examination	√ (Standard care)					

Study timeline	Baseline	Day 0	Day 1 – 6 weeks	6 months	12, 24 months (or close out visit if <24 months)	> 2 years
Weight and Height	√ (Standard care)					
Standard care bloods (complete blood count, blood chemistry i.e. U&E, glucose)	√ (Standard care)					
Past medical history (see HCRU below)	√ (Standard care)					
Drug therapy	√ (Standard care)		√ (R)	√ (R)	√ (R)	
Urine testing (vasoactive drugs)		√ (R)		√ (R)	√ (R)	√ (R)
Coronary angiogram		√ (standard care)				
Randomisation		√ (R)				

Study timeline	Baseline	Day 0	Day 1 – 6 weeks	6 months	12, 24 months (or close out visit if <24 months)	> 2 years
Coronary function test[2]<br>Resting pressure indices (Pa, Pd)<br>FFR<br>Mean rest transit time<br>Mean hyperaemic transit time<br>CFR<br>IMR<br>Absolute coronary flow		√ (NHS Support - Treatment cost)				

Study timeline	Baseline	Day 0	Day 1 – 6 weeks	6 months	12, 24 months (or close out visit if <24 months)	> 2 years
Absolute coronary resistance<br>Acetylcholine infusion (10-6M, 10-5M, 10-4M)<br>Final Ach provocation (100mcg)						

Study timeline	Baseline	Day 0	Day 1 – 6 weeks	6 months	12, 24 months (or close out visit if <24 months)	> 2 years
**MRI sub-study parameters (optional)**<br><br>LV function (including ejection fraction and strain)<br>LV volumes<br>LA function<br>LA volumes<br>Aortic distensibility<br>T1 (global, segmental)			√ (R)			

Study timeline	Baseline	Day 0	Day 1 – 6 weeks	6 months	12, 24 months (or close out visit if <24 months)	> 2 years
Extracellular volume (ECV)<br>Rest perfusion (global, segmental)<br>Myocardial perfusion reserve index<br>Summed stress score<br>Myocardial blood flow						

Study timeline	Baseline	Day 0	Day 1 – 6 weeks	6 months	12, 24 months (or close out visit if <24 months)	> 2 years
**Vascular sub-study (optional)**<br><br>Skin biopsy			√ (R)			
**Blood sample**<br><br>endothelial cytokines (ET-1, ICAM, VCAM, p-selectin, IL-6)		(R)	√			

Study timeline	Baseline	Day 0	Day 1 – 6 weeks	6 months	12, 24 months (or close out visit if <24 months)	> 2 years
Glycaemic status (plasma glucose, HbA_{1C}, lipids), metabolomics, hs-troponin, hsCRP NT-proBNP. The buffy coat will be stored for DNA. Residual					√ (R)	

Study timeline	Baseline	Day 0	Day 1 – 6 weeks	6 months	12, 24 months (or close out visit if <24 months)	> 2 years
blood will be saved for future analyses of interest.						
Follow-up contact (by telephone initially or by letter or clinic review if required).				√ (R)	√ (R)	√
Adverse events evaluation and reporting				√ (R)	√ (R)	√
Health Care Resource Use (HCRU, electronic linkage to inform the health economic analysis)	√			√	√	√
Long term follow up with electronic record linkage						√

1. Informed consent must be obtained on the first day of admission prior to the standard care angiogram. No further scheduled study assessments can be performed until full informed consent is obtained.

2. Coronary function tests should be measured in all subjects with the results disclosed (‘Disclosed group’) or not (‘Usual care group’) according to the randomly assigned treatment group.

3. The time point for the final assessment is defined as when the last participant has completed 12 months follow-up.

4. Clinical activity is identified as ‘Standard Care’ or ‘NHS Support’, ‘NHS Treatment’ or ‘Research’.

5. Health Care Resource Use (HCRU) and adverse events will be assessed by e-linkage with NHS electronic health records in primary and secondary care, including prescriptions. HCRCU data will be assessed as part of the medical history for up to 12 months before participation in the trial and then during long-term follow-up.

6. CMR parameters

- LV end-diastolic volume index (LVEDVI)
- LV end-systolic volume index (LVESVI)

- LV ejection fraction (LVEF)
- Longitudinal strain (global)
- Circumferential strain (global)
- Left atrial volumes and function
- Aortic distensibility = (aortic area max. – aortic area min) / Aortic area min x pulse pressure
- Native T1 (global, regional)
- Post-contrast ECV (global, regional)
- Rest perfusion (global, segmental)
- Myocardial perfusion reserve index
- Summed stress score (perfusion)
- Myocardial blood flow (global, regional)

- MVO on first pass (% of LV) gadolinium enhancement MRI
- MVO extent (present / absent) on late (10-15 min post-contrast administration) gadolinium enhancement MRI
- Infarct size (% LV mass)
- Aortic distensibility

1. INTRODUCTION

1.1 Background

In recent years, mortality from ischaemic heart disease (IHD) in the United Kingdom (UK) has fallen in men but not in younger women [1-3]. Each year, there are more than 20,000 new cases of angina [3], and ~240,000 invasive coronary angiograms performed[4]. However, obstructive coronary artery disease (CAD) is only detected in ~40-50% of these procedures[4-8]. Despite the apparently reassuring findings, patients with a 'negative' angiogram have an increased long term risk of cardiovascular events[8]. Although the factors associated with a low-yield from invasive angiography are multifactorial[5-8], abnormal coronary function may be a contributing factor (e.g. coronary microvascular angina (CMA)[9]. The prevalence of this condition is uncertain but it may occur in approximately one third of patients with a 'negative' angiogram [10-12].

Pathophysiology: So what is coronary microvascular angina? Angina pectoris, derived from the Latin verb 'angere' (to strangle), is chest pain of cardiac origin. Angina involves a relative deficiency of myocardial oxygen supply (i.e. ischaemia) and typically occurs after activity or stress. Angina may occur in patients with obstructive CAD, but, paradoxically, excluding systemic problems, e.g. anaemia, angina may occur in patients with non-obstructive CAD (<50% stenosis) or even in patients with a normal angiogram. These patients may have Type 1 CMA [5-12]. CMA is a chronic condition, although patients may present with symptoms acutely, and recurrently.

CMA is associated with female sex [13], vascular risk factors[14-23], and spontaneous or inducible coronary vasoconstriction[24-27]. CMA has an adverse prognosis[10-13], and is identified in practice guidelines as a problem of unmet need[27,28].

Endothelin-1 (ET-1) is implicated in the pathophysiology of CMA (Appendix 2, Schematic pathobiology diagram). ET-1 is a 21-amino acid peptide that is released mainly by endothelial cells, but other cells such as vascular smooth muscle (VSMCs), are also sources[29-32]. ET-1 is a highly potent vasoconstrictor via its VSMC receptors (ET_A, ET_B), but it also has pleiotropic effects. ET-1 is mitogenic, pro-oxidant, pro-inflammatory, and inotropic and ET-1 also regulates renal fluid and electrolyte homeostasis[30-32]. ET-1 augments vascular tone constitutively (i.e. flow-mediated) and under stress[29], and local ET-1 activity reflects bioavailable vasoconstrictor and vasodilator chemicals[33], especially those derived from the endothelium[34]. ET-1 is implicated in the pathogenesis[35-37] and progression of CAD[37].

ET_A receptors mediate vasoconstriction[30,31,38]. ET_B receptors are located on endothelial- and VSMCs, and ET_B has nitric-oxide (NO)-dependent vasodilator effects in healthy blood vessels[52, 53], or vasoconstrictor effects if NO is deficient[30;39,40]. In pulmonary resistance arteries the constrictor response to ET-1 is biphasic and varies with ET-1 concentration[41]. Selective pharmacological antagonism of ET_A and ET_A/ET_B has confirmed that ET-1/ ET_A/ET_B regulate resting and stimulated forearm blood flow in patients with CAD[42]. ET-1 enhances coronary vascular tone in vivo via ET_A-activation[43-45], it contributes to coronary endothelial dysfunction[44] and its tonic effect on myocardial perfusion, as revealed by PET, is related to the presence & extent of risk factors for atherosclerosis[46].

ET-1 dysregulation and CMA: Kaski et al[47] observed that in patients with angina and normal coronary arteriograms, circulating ET-1 concentrations were increased and associated with a shorter time to onset of angina during exercise. In 35 hypertensive patients without CAD, Fuji et al[48] observed that the circulating concentration of ET-1 was a multivariable associate of coronary flow reserve (CFR).

Based on an analysis of 1034 consecutive subjects who underwent rest/dipyridamole PET with ^{82}Rubidium for the assessment of IHD, Johnson et al[49] identified an abnormal pattern of diffuse heterogeneous myocardial perfusion that was associated with coronary microvascular disease (CMD). In a large animal study, they reproduced this abnormal perfusion pattern using intra-coronary (IC) infusions of ET-1 infusion[50] implying ET-1-contributes to enhanced coronary tone in patients with CMA[49,50].

Clinical pharmacology of ET-1 receptor antagonists in CMA ET-1 & forearm blood flow In a study of patients with Syndrome X (a historical name for CMA), Newby et al[52] observed similar blood flow responses to basal and stimulated NO-mediated vasodilatation and to intrabrachial artery infusion of BQ123 (an ET_A receptor antagonist (ERA)), as compared to controls. By contrast, the forearm constrictor response to ET-1 was ~15% less in CMA patients vs. controls. They observed an inverse correlation between ET-1-induced vasoconstriction and plasma ET-1 levels, and concluded that since Syndrome X patients may have chronically increased plasma ET-1 levels[47], ET_A receptors may be down-regulated.

ET-1 & coronary blood flow: McCarthy et al[43] and Halcox et al[44,45] noted that intra-coronary BQ123 caused coronary vasodilatation in patients with angina and CMA, whereas BQ788, an ET_B-RA, caused coronary vasoconstriction, implicating ET_B in mediating coronary vasodilatation under resting conditions [45]. In a randomised, placebo-controlled trial of the oral ET_A-RA (atrasentan, 10 mg PO daily) administered for 6 months in 47 patients with CMD, Reriani et al[53] observed that chronic ET_A-RA therapy improved coronary microvascular endothelial function as revealed by IC-ACh. However, they could not deduce whether this ET_A-mediated effect was due to enhanced vasoconstriction, increased breakdown of NO due to oxidant stress, or both. Building on their studies of perfusion patterns with ^{82}Rb-PET, Johnson et al[54] observed that in patients with heterogeneous reductions in resting myocardial perfusion (consistent with CMA), treatment with the ET_A-RA, darusentan,

improved myocardial perfusion and increased the homogeneity of the perfusion pattern. They concluded that in patients with CMA, ET-1 caused regional reductions in myocardial perfusion and these abnormalities could be improved by ET_A receptor blockade with darusentan. In a study of CFR in patients with diabetes involving intracoronary administration of 500 mg of BQ123 or placebo, CFR was higher in the patients treated with the ET_A-RA (n =10; 3.1±0.7) compared with placebo (n=5, 2.3±1.2; $p<0.05$), or non-diabetic controls (4.9±2.3)[55].

The case for ET receptor antagonist therapy in CMA Taken together, these studies[42-55] implicate dysregulation of the endothelin-1 (ET-1) system in CMA, and ERA therapy has been associated with preliminary evidence of benefit. On the other hand, when ERAs have been assessed in clinical trials in hypertension (HTN) and heart failure (HF), side-effects e.g. fluid retention, were problematic and ERAs are not recommended[56,57]. On the other hand, in pulmonary arterial hypertension (PAH), ERA therapy, such as ambrisentan, bosentan and macitentan, has a Class 1 recommendation[58], and other novel approaches including ET_B-receptor agonists, and inhibitors of the endogenous synthesis of ET-1 (i.e. neutral endopeptidase (NEP)/endothelin converting enzyme (ECE) inhibitors are in development[59]. In PAH, the benefits of ERA therapy outweigh the side-effects, unlike in HTN and HF. Since the pathophysiology of ET-1 dysregulation in CMA is incompletely understood, and novel alternative pharmacological approaches are emerging, our strategy in this proposal aims firstly to address the gap between diagnostic tests and treatments (clinical study) and secondly to focus on mechanistic studies to better understand ET-1 dysregulation in well characterised patients with CMA and matched controls. Taken together our research is intended to inform and optimise future therapeutic strategies for CMA.

Our scientific hypotheses are that ET-1 dysregulation enhances coronary and peripheral microcirculation tone in patients with CMA through one or more of the following potential mechanisms:

Endothelial dysfunction, associated with increased oxidant production and reduced bioavailable NO, unmasks a susceptibility to ET-1 vasoconstriction, under resting and/or reactive conditions; Enhanced ET_A-activation leads to a preponderance of ET-1-mediated vasoconstriction. Reduced ET_B-function leads to a preponderance of ET-1-mediated vasoconstriction. To test these hypotheses, we will investigate the mechanisms of ET-1 dysregulation using ERAs in controlled isometric tension studies in peripheral arterioles obtained from patients with or without CMA.

3.2 Statement of the problem: There is a critical missing link between the use of relevant diagnostic tests (invasive tests of coronary reactivity; non-invasive perfusion MRI), drugs with known efficacy (e.g. verapamil), and prognosis. This conundrum is apparent in the NICE-95 guidelines for Chest Pain 'in people with "typical angina-like chest pain if investigation excludes flow-limiting disease in the epicardial coronary arteries", then 'Syndrome X' should be considered' [28], but no other guidance is provided.

CFR reflects the vasodilator capacity of the epicardial artery and its microcirculation (normal >2.0)[60-68]. The index of microvascular resistance (IMR) is a measure of microvascular resistance (normal <25)[69-78], and the resistive reserve ratio (RRR) reflects microvascular vasodilator capacity (normal >2.0)[75]. Pressure sensing coronary guidewires are routinely used in the NHS for measurement of fractional flow reserve (FFR)[4,79-82]. Recently, a new and simpler approach has emerged for measurement of absolute coronary flow and resistance. The method involves intra-coronary infusion of saline using a custom-designed catheter (Rayflow, Hexacath, Fr) and the standard diagnostic guidewire and thermodilution. In the current study absolute coronary flow and resistance will also be measured.

Intra-coronary (IC-ACh) provides additional information on the vasodilator capacity of the coronary circulation[8-27], but ACh is not licensed for parenteral use and is rarely used for diagnostic clinical testing in the NHS, mainly because of an incomplete evidence base and the need for pre-preparation by pharmacy. We have recently completed a clinical trial of remote ischaemic conditioning in patients undergoing elective coronary angiography (RIC-COR: Clinicaltrials.gov NCT02666235[84]). The ACh administration was successfully completed without complication in 60 patients.

Lee et al[67] undertook a prospective single-centre study of the diagnostic utility of CFR, IMR and IC-ACh in 139 patients with angina, some evidence of atherosclerosis on intravascular ultrasound but no evidence of obstructive CAD on angiography. They found that a vasoconstrictor response to IC-ACh in the left anterior descending coronary artery (>20% reduction in diameter, consistent with abnormal endothelial function), or an abnormal IMR (≥25) or CFR (<2.0), occurred in 44%, 21% and 7% of patients, respectively. Surprisingly, myocardial bridging was also a common observation (58%). Using these diagnostic tests, 77% of patients had at least one potential explanation for angina and, importantly, IC-ACh had higher sensitivity for abnormal coronary reactivity than either IMR or CFR. Female sex was a multivariable associate of a lower CFR[68]. Overall, this clinical study is important because it indicates that functional testing may have diagnostic utility for identifying patients with microvascular angina, as compared with invasive angiography only.

The study also raises important questions and gaps in evidence that can only be addressed in a randomized, controlled trial:

1) Are the findings in this single centre study transferrable to routine practice?

2) What is the prevalence of CMD in all-comers with angina but without obstructive CAD;

3) Is adjunctive functional testing routinely feasible, and if so do the results of these tests change the treatment plan that is initially based on angiography alone?

4) Finally, if the tests results are linked to therapy, what is the impact on health status?

We have experience with guidewire-based coronary reactivity testing in single-[72,73] and multi-[74,77,78] centre studies, including the BHF-funded CE-MARC2 trial[83]. Although we[84] and others[10-12,24,25,67,68,85] have used IC-ACh for research purposes, since IC-ACh is off-label it rarely used during standard NHS procedures [86]. Our recent experience with ACh administration in 60 participants in the RIC-COR study was favourable[84]. We have addressed the gap in access to ACh during routine care through work with our Trials Pharmacy in order to provide cardiologists with a pre-prepared pack for IC-ACh testing that would be stored in the catheter laboratory and so immediately available for ad hoc use in daily practice. If the results of our study are favourable, the information would support a change in license, and/or the availability of ACh packs for IC use in the NHS.

Non-invasively, stress perfusion CMR has potential to disclose microvascular disease. However, evidence is lacking to support the use of stress CMR for this purpose. On the one hand, the standard NHS tests for CAD i.e. coronary CT, stress echocardiography and exercise testing, lack sensitivity for CMD. On the other hand, stress testing with PET[13,14,49-51,54], CMR[27,87-94] and Doppler-derived CFR using echocardiography[95] have potential diagnostic and clinical value for CMD. However, not all of the data with advanced imaging are supportive[96], and the costs and logistics of these tests preclude their routine use, especially in the absence of evidence from randomised trials. Consequently, current non-invasive diagnostic approaches may rule out obstructive CAD but fail to detect patients with microvascular angina, leading to an alternative (incorrect) diagnosis, and potentially false reassurance with inappropriate cessation of therapy.

Fundamentally, there is an absence of evidence from randomised trials that a diagnostic strategy which is linked to therapy improves the wellbeing of patients with angina without obstructive CAD (to rule-in or rule-out CMA). This knowledge gap results in adjunctive tests and related treatments rarely being used in the NHS[4] (Golden Jubilee pilot). Put in other words, since there is no evidence that starting a treatment based on functional testing improves wellbeing, clinicians will not use the tests in the first place. More research is needed to confirm or refute that a routine diagnostic strategy with functional tests appropriately rules in or out microvascular disease, and improves prognosis and resource utilisation in patients with angina and a 'negative' angiogram. To date, there has never been a strategy trial of adjunctive tests linked with therapy in this population. Other current studies, such as the iPOWER[95] natural history study, lack of random allocation of a diagnostic strategy, and will not address this gap.

The focus of our research is the clinical conundrum of microvascular angina and the key gaps in knowledge on the vascular biology and diagnostic management of this condition. The prevalence of microvascular angina is estimated to be ~30 (22 – 44)% in patients with chest symptoms who did not have obstructive CAD[8,10-12,97], and this condition is prognostically important[8,13,98-100]. We will undertake a proof-of-concept clinical study of a routine diagnostic strategy during NHS practice and in parallel we will investigate new questions in on endothelial dysfunction and ET-1 dysregulation. The clinical study will serve as a vehicle to classify and enrol ~240 patients in Glasgow into the nested vascular biology study. The design involves an internal pilot study (n=150; Appendix 3) to gather information on feasibility and to optimise implementation. After the completion of the study, we will use the new knowledge to optimize the design of a larger 4-centre trial in approximately 480 subjects.

Clinical relevance of CFR and IMR abnormalities

A reduced CFR (<2.0) is associated with an adverse prognosis, including in patients without obstructive CAD. Similarly, an increased IMR (>25) is also prognostically important. A reduced CFR reflects a depressed vasodilator capacity of the coronary microcirculation whereas an increased IMR reflects increased microvascular resistance.

Myocardial perfusion revealed by cardiac magnetic resonance imaging

Non-invasively, stress perfusion CMR has potential to disclose microvascular disease [27,87-94], however, because of limited evidence on diagnostic and clinical utility, costs and logistics, CMR is not routinely used for this purpose. On the other hand, current non-invasive diagnostic approaches that are clinically indicated to detect obstructive CAD lack sensitivity and specificity for coronary microvascular disease, and so may fail to detect patients with microvascular angina. A negative test result may lead to an alternative (incorrect) diagnosis, and potentially false reassurance with inappropriate cessation of therapy. Therefore, more research is warranted to better understand the diagnostic accuracy of stress CMR for coronary function measured invasively, and whether or not stress CMR has clinical and prognostic value.

Overall, there is an absence of evidence from longitudinal studies trials on the inter-relationships and comparative prognostic significance of parameters of coronary function and myocardial perfusion (as revealed by stress CMR) in relation to symptoms, health status and health outcomes.

1.2 Standard of care

Invasive coronary angiography is the standard of care for assessing patients with known or suspected CAD. Guidewire-based adjunctive testing may be combined with coronary

angiography in order to assess the functional significance of a coronary stenosis by measurement of the myocardial FFR. Guidewire-based coronary reactivity testing is the standard approach to measurement of coronary microvascular function, although such tests are uncommonly used, mainly because of a lack of clinical evidence. IC-ACh is also a standard of care for the assessment of microvascular angina in some centres outwith the UK, however IC-ACh provocation testing is seldom performed because aseptic preparation is time consuming and the medication is unstable in solution expiring within 30 hours.

1.4 Study rationale - hypothesis

Rationale: Our study focuses on patients with known or suspected angina undergoing coronary angiography. Patients in whom the angiogram has ruled out obstructive coronary disease and in whom medical management is intended will be eligible to be randomised. The rationale of the study is to assess whether a routine strategy of adjunctive testing of coronary function will lead to changes in the diagnosis and management of the study participants, and whether or not a treatment plan informed by the results of coronary function might be associated with differences in health and well being, compared to standard care without knowledge of coronary function. A second rationale is to undertake research into the pathophysiology of angina and coronary function by undertaking blood tests, stress perfusion cardiac MRI, and assessments of blood vessel function non-invasively and in the laboratory using small blood vessels isolated from skin biopsies.

Hypotheses (Hø)

Hø1: Abnormal coronary artery reactivity is common in patients with angina without obstructive CAD.

Hø2: Compared with the medical decisions based on the angiogram alone, disclosure of functional test results commonly changes the diagnosis (to rule in- or rule out CMA) and onward clinical management (tests and treatment) by clinicians.

Hø3: A strategy of adjunctive testing of coronary function is associated with improvements in health status (quality of life, symptoms, functionality as assessed by questionnaires) and is associated with data to support potential cost-effectiveness. The basis for this hypothesis is that the additional information clarifies the diagnosis, and leads to cessation of inappropriate or unnecessary tests and treatment, and initiation of more appropriate management. The decision for changing the diagnosis (and therapy) relates to specific diagnostic thresholds, and linked therapies informed by practice guidelines, such as those from the European Society of Cardiology. Clarification of the diagnosis per se (rule-in / rule-out) may be associated with improved patient satisfaction and health status. The results from this pilot study are intended to provide preliminary evidence of efficacy with an overall aim of informing the design and scale of a larger definitive clinical trial.

Hø4: Stress CMR findings have a high correspondence with coronary reactivity responses, supporting the validity of stress CMR for the non-invasive detection of coronary microvascular disease.

Hø5: Novel parameters of myocardial perfusion e.g. transmural perfusion gradients, have a higher correspondence with CFR, IMR, and ACh-responses than visual interpretation of the CMR scan.

Hø6: In CMA, abnormal coronary function is closely associated with vascular density in peripheral tissue e.g. tongue.

Hø7: Compared to resistance arteries from controls without CMA, resistance arteries from patients with CMA have (1) impaired endothelium-dependent vasorelaxation, (2) enhanced

constrictor-responses to ET-1, (3) enhanced response to acute ET_A inhibition implying increased receptor function and/or density, and (4) reduced vasodilator response to ET_B inhibition, indicating that ET_B mediated-vasodilatation is deficient in patients with CMA and contributes to increased vascular tone.

Hø8: Coronary function parameters will be associated with circulating molecules that are implicated in the pathophysiology of abnormal coronary vascular function including 1) endothelial cytokines (ET-1, ICAM, VCAM, p-selectin, IL-6), 2) endothelial microparticles, 3) glycaemic status (plasma glucose, $HbA_{1C,}$ lipids), 4) metabolomics, 5) hs-troponin, 6) NT-proBNP.

Hø9: Compared to the usual care group, NT-proBNP concentration measured during follow-up will be less in the patients randomized to the disclosed group.

Hø10: Compared to the usual care group, NHS resource utilization will be less in the disclosed group. On the one hand, the use of adjunctive tests of coronary function will expectedly increase the cost of standard care. On the other hand, resource use may reduce in the following ways. If abnormal coronary function is ruled out, then medications can be appropriately stopped. Clarification of the diagnosis may prevent unnecessary downstream tests and treatments. If abnormal coronary function is confirmed, appropriate therapies can be introduced that theoretically should be associated with improvements in well-being, and additional tests and attendances in primary and secondary care may not be necessary. Finally, clarification of the diagnosis linked with appropriate therapy may have prognostic benefits. The current pilot trial is designed but not powered to assess for adverse events such as myocardial infarction. The availability of information on health status e.g. EQ5D, combined with information on healthcare resource use, will be used in an exploratory health economic analysis that is intended to inform the rational and design of a larger more definitive trial.

Rationale for health status as an outcome: the study population will have undergone clinically-indicated coronary angiography, which is usually performed based on a history of symptoms. Our patient-orientated research focuses on health status, as reflected by patient responses in validated questionnaires on angina, functionality and well-being. The metrics that will be used are the Seattle angina and EQ-5D questionnaires, and the assessments will be performed at baseline, 6, 12 and 24 months later (or the end of the study whichever is sooner).

Rationale for using cardiac MRI: CMR is an established method for the assessment of heart size and function. In addition, multi-parametric CMR provides information on myocardial tissue characteristics and perfusion. All of this information can be obtained in a single scan and so is spatially and temporally registered. In addition, the CMR information can be correlated with the findings from invasive coronary angiography and coronary reactivity testing. CMR has greater measurement accuracy than echocardiography, and since CMR does not involve ionising radiation it can be safely repeated (unlike nuclear imaging). We will implement an optional CMR as a sub-study for those patients who are eligible for CMR and for whom scanning is feasible. The standard CMR results may provide clinically relevant information and will be disclosed in order to inform patient care. The impact of the CMR results on the patient's diagnosis, i.e. diagnostic utility, will be prospectively assessed using a questionnaire.

Myocardial perfusion may be imaged by CMR during myocardial 'first pass' of gadolinium contrast and a regional failure of contrast to 'wash-in' reflects microvascular obstruction (MVO). MVO can be assessed subsequently early (1 minute) or late (10-15 minutes) after gadolinium administration. As an extracellular contrast agent, gadolinium diffuses from capillaries into the interstitial space. Because of this diffusion, the extent of the wash-in deficit revealed by first pass CMR diminishes over time as revealed by early and late

gadolinium enhancement Assessment of myocardial perfusion during stress compared to rest provides information on inducible abnormalities of myocardial perfusion that may be attributable to obstructive CAD or microvascular disease.

Rationale to mask the *stress* CMR results: The clinical decisions using functional data are based on usual care measurements of coronary function at the time of invasive angiography. The comparative significance of myocardial perfusion (concordant/discordant) versus coronary function is uncertain. Since patients will be enrolled based on standard care angiography with tests of coronary function, and the stress CMR protocol is for research purposes, the stress perfusion results will not be disclosed. By doing so, we can undertake a natural history study of the clinical significance of the CMR findings and compare against the standard care tests of stenosis severity and coronary function. The results of the rest of the CMR scan (mass, function, incidental findings) will be reviewed and clinical reports will be provided.

Adenosine stress perfusion CMR: In practical terms, a protocol involving stress CMR may last up to one hour which is generally well tolerated. In terms of feasibility, although there are some contra-indications to CMR (e.g. permanent pacemaker, severe claustrophobia), stress CMR is widely performed for clinical and research purposes across the UK.

Rationale for blood sample: A blood sample will be obtained at baseline and again at 12 months. The samples will be centrifuged and stored in the laboratories of the Golden Jubilee National Hospital and the BHF Glasgow Cardiovascular Research Centre. These tests are not routine care.

The specific rationale for biomarker tests to provide insights into coronary microvascular pathophysiology and to provide information on a surrogate outcome, NT-proBNP:

1) Endothelial cytokines (ET-1, ICAM, VCAM, p-selectin; IL-6)

2) Endothelial microparticles.

3) Glycaemic status (plasma glucose, $HbA_{1C,}$ lipids),

4) metabolomics,

5) hs-troponin, hsCRP

6) NT-proBNP. NT-proBNP: a prognostically validated biomarker of cardiac function and survival.

Separate to these analyses, any sample excess would be stored for future scientific analyses (based on future research ethics committee approval).

Rationale for angiographic parameters of coronary disease and vascular function

We will measure prognostically validated angiographic indices of coronary artery blood flow (TIMI coronary flow grade[137]) and microvascular function (TIMI perfusion grade[139,140], TIMI frame count [139,140]). These parameters will provide information on coronary vascular function. A TIMI frame count is < 27 frames is taken as normal.

Information from other usual care tests

Clinical data from the medical history (including left ventricular function and LVEDP) and medication will be collected from the medical records.

Rationale for an internal pilot

The pilot is described as 'internal' reflecting the NIHR definition whereby the study serves as a rehearsal for a larger definitive trial. The data from the pilot trial will contribute to that trial's final dataset.

1.5 Relevance to the NHS

Our study is intended to be applicable to clinical practice in the NHS. Focusing on patients with known or suspected angina with a treatment plan for medical management, our study will provide new information on the pathophysiology of coronary function and myocardial perfusion, the comparative clinical significance of the results of coronary reactivity tests, including relationships with patient-orientated outcomes, including 'hard' endpoints, such as mortality. Our study has the potential to contribute significantly to the scientific understanding and broader knowledge of the mechanisms and treatment of coronary microvascular disease which are currently poorly understood. The study fits well with the clinical guidelines and gaps in clinical evidence [21].

2. PILOT STUDY OBJECTIVES

CORonary MICrovascular Angina (CorMicA) PILOT: a randomised, controlled proof-of-concept strategy trial comparing routine invasive testing of coronary function during diagnostic coronary angiography vs. standard care to inform diagnosis and management of patients with stable angina.

2.1 Primary objective

To assess whether a diagnostic strategy involving invasive tests of coronary function is routinely feasible, safe, impactful on diagnosis and management.

2.1.1 Primary outcome

Within-subject change at 6 months from baseline for chest discomfort as revealed by the Seattle angina questionnaire

2.2 Secondary outcomes

2.2.1 Secondary objectives

1. To assess feasibility of the adjunctive diagnostic strategy including recruitment rates, compliance with the protocol, integrity of blinding in the catheter laboratory, cross-over between groups during or after the procedure, adherence with medication during follow-up, compliance with follow up.
2. To assess safety of the tests of coronary function
3. To assess the prevalence and type of microvascular dysfunction
4. To assess impact of disclosure of the coronary function test results on the diagnosis and certainty of the diagnosis (diagnostic utility),

5. To assess impact of disclosure of the coronary function test results on clinical management (including treatment and investigations),
6. To assess the relationships between cardiovascular risk factors, reflected by validated risk scores (e.g. ASSIGN, JBS3), and parameters of coronary function, in medically managed patients.
7. Health status: Rose angina, Seattle angina scores (5 components), EQ5D health status, Illness perception (Brief IPQ), anxiety/depression (PHQ4) , treatment satisfaction (TSQM) at baseline and during follow up. Functional status (DASI) and physical activity levels (IPAQ-SF).
8. To assess the early impact of the randomised strategy as revealed by the within-subject change on treatment satisfaction (SAQ) and illness perception (Brief IPQ) post-procedure vs. baseline.
9. To assess associations between circulating molecules that are implicated in the pathophysiology of coronary disease between the randomised groups
10. NT-proBNP, a surrogate outcomes of prognosis
11. To assess whether patients with CMA have evidence of abnormal microvessel density (tongue video-microscopy)
12. MRI sub-study: Myocardial perfusion by quantitative stress CMR.
13. MRI sub-study: To determine the diagnostic accuracy of stress perfusion CMR at 1.5 Tesla (as assessed visually) for CMD in invasively managed patients.
14. Vascular function sub-study: To assess whether patients with CMA have abnormal vascular function (in vitro studies of vascular function), if so what mechanisms may be involved and whether pharmacological agents e.g. ET receptor antagonists, sacubitril/valsartan, might restore function in vitro.
15. NHS resource utilisation including primary and secondary care costs for tests, procedures and out-patient visits, and medicines.

16. To undertake an exploratory health economic analysis that will inform the rationale and design of a larger definitive trial.
17. To assess compliance with therapy and lifestyle changes (smoking, diet, weight, exercise) associated with diagnostic strategy.

2.3. Assessments for the primary and secondary outcomes

2.3.1 CARDIOLOGIST QUESTIONNAIRE (Appendix 2)f

Catheter laboratory/cardiology ward: coronary function tests (post-randomisation vs. Pre-randomisation based on angiography alone)

Frequency of diagnosis

1. What is the likelihood of coronary heart disease: *no, unlikely, probable, or yes*
2. What is the likelihood of angina due to obstructive coronary heart disease (i.e. >70% stenosis in a main branch or >50% in the left main stem): *no, unlikely, probable, or yes*.
3. What is the likelihood of angina due to a disorder of coronary function (i.e. microvascular angina or vasospastic angina): *no, unlikely, probable, or yes*. (prioritised outcome0
4. What is the likelihood of a non-cardiac cause of chest pain: *no, unlikely, probable, or yes*.

Frequency of diagnosis is defined as yes/probable vs unlikely/no

Certainty of diagnosis

Certainty is defined as yes/no vs unlikely/probable

Treatment

Change in treatment plan (see Appendix 2)

Investigations

Change in plan for tests (see Appendix 2)

Clinical management

Change in management plan (see Appendix 2)

CATHETER LABORATORY FINDINGS

ANGIOGRAM (USUAL CARE)

TIMI angiographic blush grade

SYNTAX score

Cumulative coronary stenosis score

Gensini score

LV end-diastolic pressure

Rate pressure product

2.3.2 CORONARY PHYSIOLOGY (DIAGNOSTIC PARAMETERS MEASURED DURING USUAL CARE OR CLINICAL RESEARCH)

Fractional flow reserve (FFR)

Coronary flow reserve (CFR)

Resistance reserve ratio (RRR)

Index of microvascular resistance (IMR)

Novel parameters:

Absolute coronary blood flow (ml/min)

Absolute coronary resistance (mmHg.min.ml^{-1} or Wood units)

Coronary artery diameter change (% from baseline) in response to acetylcholine & glyceryl trinitrate

Coronary endothelial dysfunction (a decrease in luminal diameter of >20% after intracoronary infusion of acetylcholine).

Coronary artery spasm (focal or diffuse, macrovascular or microvascular)

2.3.3 DRUG THERAPY

Medications used for treatment of cardiovascular disorders, including primary and secondary prevention therapies (including reasons for self-discontinuation)

2.3.4 BIOCHEMISTRY (baseline and 12 months)

Glycaemic Status (plasma glucose, HBA1c, lipids)

hs-Troponin

NT-pro BNP

hs-CRP

ET-1,

ICAM, VCAM, p-selectin, IL-6

Endothelial microparticles

Change in ET-1

Change in ICAM, VCAM, p-selectin, Il-6

Change in endothelial microparticles

Change in NT-pro BNP

These samples will be analysed when funding is secured

2.3.5 PERIPHERAL ENDOTHELIAL FUNCTION ASSESSMENT

Non-invasively using tongue videomicroscopy (baseline only)

2.3.6 PERIPHERAL ARTERIOLAR FUNCTION ASSESSMENT

Optional sub-study at baseline

Gluteal skin biopsy analysis of small arteriolar vasoactive function assessed in isolated resistance arteries – in vitro wire myography studies of vasoactive responses to ACh, sodium nitroprusside, ET-1, novel pharmagological antagonists in vitro (e.g. ET receptor antagonist, Sacubitril/valsartan etc., as per a defined laboratory standard operating procedure (SOP)

2.3.7 HEALTH STATUS (baseline, 6, 12 and 24 months (or end of study))

Seattle angina score

EQ5D-5L assessment

Illness perception (Brief IPQ)

Anxiety/depression (PHQ4)

Treatment satisfaction (TSQM) at baseline and during follow up

Change in Seattle angina score from baseline

Change in EQ5D-5L from baseline

Lifestyle (diet, exercise, smoking, self-reported weight).

Functional status (DASI)

Activity levels (IPAQ-SF)

2.3.8 CMR (sub-study)

LV end-diastolic volume index (LVEDVI)

LV end-systolic volume index (LVESVI)

LV ejection fraction (LVEF)

Longitudinal strain (global)

Circumferential strain (global)

Left atrial volumes

Left atrial function

Native T1 (global, regional)

Post-contrast ECV (global, regional)

Rest perfusion (global, segmental)

Myocardial perfusion reserve index

Summed stress score (perfusion)

Myocardial blood flow (global, regional)

MVO on first pass (% of LV) gadolinium enhancement MRI

MVO extent (present / absent) on late (10-15 min post-contrast administration) gadolinium enhancement MRI

Infarct size (% LV mass)

Aortic distensibility

Diagnostic utility (impact of disclosure of the CMR results on the diagnosis and management)

2.3.9 LONGER-TERM FOLLOW-UP

Health outcomes (death, cardiovascular death, non-fatal myocardial infarction, hospitalisation for heart failure, non-fatal stroke or transient ischaemic attack, resuscitated cardiac arrest, cardiac devices (including implantable defibrillator and resynchronization therapy), and coronary revascularisation). Health outcomes will be assessed at 6, 12 and 24 months (or the end of the study, whichever comes sooner). NB Patient contact is necessary for the blood test and health status and questionnaire check at 12 months. At the other time-points, patient contact will not be necessary if access to electronic medical records is

feasible and the data are clear. When patient contact is unsuccessful the GP may be contacted, and electronic health records will be checked including for the assessment of events, angina scores and medication use in the longer term.

The follow-up will be by telephone, or by letter or clinic review as clinically appropriate. In the longer term, additional follow-up of health outcomes will be assessed by electronic case record linkage using the NHS number (England) and Community Health Index (CHI) number in Scotland.

3. STUDY DESIGN

CorMicA PILOT is a prospective, double blind, randomized-controlled pilot trial comparing two management approaches to the clinical problem of patients with stable angina without obstructive coronary disease on invasive angiography. A precise physiologically guided diagnostic approach will be compared with a usual care approach based on standard coronary angiography alone.

Coronary function tests are not standard of care and are very rarely undertaken in clinical practice (see pilot data). The rationale for our study reflects the lack of any evidence that treatment associated with these tests improves health and well-being. Blinding of the cardiologist and patient to the coronary function test results can be justified to ensure a balanced distribution of patient characteristics between the treatment groups, to facilitate optimal standard care in the control group, to minimise bias, and so ensure that the main difference between the two groups is the diagnostic management strategy. The strategy involves ad hoc adjunctive testing of coronary function (disclosed) vs. standard care with invasive angiography only (coronary function measured but not disclosed).

The MHRA have classed CorMicA as a non-CTIMP (May 2016). This study will be performed according to the Research Governance Framework for Health and Community Care (Second edition, 2006). All investigators and key trial personnel will complete biennial GCP training.

3.1 Study population

This pilot study will aim to enroll consecutive outpatients attending for elective diagnostic coronary angiography for investigation of confirmed angina. We anticipate enrolling 3 - 5 patients per week (over 44 working weeks) allowing a minimum of 150 subjects enrolled

within 2 years by screening approximately 1000 patients undergoing invasive coronary angiography at the Golden Jubilee National Hospital and Hairmyres Hospital sites.

The standard of care is invasive coronary angiography and guidewire based measurement of myocardial FFR, which is an index of the functional significance of a coronary stenosis.

The design involves enrolling participants with stable angina who are undergoing invasive coronary angiography which is the standard of care. Informed consent would be obtained before undergoing coronary angiography. During the angiogram, the sub-group of participants who have obstructive coronary disease excluded by angiography ± fractional flow reserve (FFR) would be eligible to take part whereas the sub-group of participants with obstructive disease confirmed by the angiogram would be excluded. Patients who are not randomised, e.g. due to the presence of obstructive disease, logistical reasons, and who have consented to the study would enter into a follow-up registry in which the follow-up assessments would be the same (including the possibility of record linkage in the longer term).

Patients will be eligible to be enrolled after the exclusion of functionally significant epicardial coronary disease on invasive angiography. The standard of care does not include the performance of adjunctive diagnostic tests of microvascular function – CFR, IMR and use of intracoronary acetylcholine (assessing coronary vasodilator capacity, microvascular resistance, and coronary endothelial function respectively). These tests are not standard care. These physiological tests will be measured in all of the study patients however the results will be blinded and only revealed in a randomized fashion to half of the treating physicians and their patients. Non-disclosure can be justified in order to permit usual care in the control group and minimise bias based on the premise that these tests are rarely undertaken in clinical practice (see pilot data) with little evidence that treatment associated with these tests improves prognosis.

3.2 Setting

Clinical activity will take place at both the Golden Jubilee National Hospital and Hairmyres Hospital. Enrolment and catheterisation laboratory work will take place in the Cardiology Department of the respective sites. The optional sub-study for CMR sub-study will take place in the Radiology Department and the optional vascular sub-study involving a skin biopsy will take place in the Beardmore Clinical Research Facility. Screening, informed consent, and the study procedures will be undertaken on an elective outpatient basis. The invitation to attend hospital will include a CorMicA Patient Information Sheet. On admission, patients will be screened by a member of the research team e.g. a research nurse, or research fellow, on the ward before undergoing elective angiography.

3.3 Inclusion criteria

The decision to enroll patients will be made prior to the invasive coronary angiogram. Patients undergoing clinically-indicated invasive coronary angiography will be identified according to those with angina or angina-equivalent (according to the Rose- and Seattle Angina questionnaires).

CLINICAL

Age ≥18 years

A clinically-indicated plan for invasive coronary angiography

Symptoms of angina or angina-equivalent (according to the Rose- and Seattle Angina questionnaires).

Specific considerations:

There is no upper age limit for inclusion.

3.4 Exclusion criteria

The clinical criteria that would exclude the patient from the study will be evaluated by medical, research and nursing staff prior to the elective angiogram according to the availability of clinical records.

CLINICAL

A non-coronary indication for invasive angiography e.g. valve disease, heart failure

During the angiogram: obstructive disease evident in a main coronary artery (diameter >2.5 mm), i.e. a coronary stenosis>50% or a fractional flow reserve (FFR) ≤0.80

Lack of or inability to provide informed consent

The optional stress CMR sub-study has the following exclusion criterion:

Contra-indication to contrast-enhanced CMR e.g. severe renal dysfunction (GFR < 30 ml/min), a non-CMR compatible pacemaker or defibrillator, or asthma with routine inhaler use.

The optional vascular sub-study has the following exclusion criterion:

Oral anti-coagulation.

3.5 Identification of participants and consent

Consecutive patients undergoing invasive coronary angiography will be screened and considered eligible according to the presence of inclusion criteria and absence of exclusion criteria. Screening will be performed by a member of the research team, e.g. cardiologist, clinical research fellow, research nurse, during and out-with office hours as appropriate. A screening log check-list with the inclusion and exclusion criteria (Appendix 1) can be created from electronic catheter laboratory records based on use of a diagnostic guidewire. The decision to enroll a patient will be documented in the clinical records.

Screening log

The screening log will record consecutive patients but without any identifiable data (Appendix 1). The log will be based on electronic records recorded in the eCRF.

3.5.1 Summary of consent procedure

A standard approach to witnessed written informed consent will be undertaken.

3.5.1.1 Informed consent in the Cardiology Department

Only patients who are sufficiently well to understand the information about the study (including the potential for benefit and known risks), as described by the attending clinical research nurse and cardiologist, would be eligible to participate. Patients who undergo invasive coronary angiography for known or suspected stable angina will be eligible and invited to give informed consent by a member of the clinical research team. Written information about the study will be given to the all outpatients scheduled to undergo invasive coronary angiography for investigation of stable angina. The discussion of the study will take place in the day ward of the cardiac catheterization laboratory prior to the

procedure. This will cover all of the points listed in the Patient Information Sheet (PIS). Clinical staff will be available to discuss the study with the patient and his/her family and friends. The patient would then be invited to provide consent in writing on the full consent form. If the patient agrees to take part in the study then the PIS form will be signed by the researcher. The decision to include a patient in the study will be documented in the medical notes and/or eCRF. No further scheduled study assessments can be performed until full informed consent is obtained. The decision to include a patient in the study during the coronary angiogram procedure is the responsibility of the attending cardiologist.

Withdrawal criteria: A participant may withdraw from the study at any time. There are no specific withdrawal criteria, although clinicians can withdraw patients as appropriate and record the reasons. These patients would also be followed-up unless consent is withdrawn.

3.5.2 Recruitment plan

Project timetable and milestones:

RECRUITMENT RATE: on average, 3 – 5 patients per week will permit us to recruit at least 150 patients within 1 – 2 years (Figure 1. Flow diagram).

PARTICIPANT DROP-OUT: Our sample size estimate allows for withdrawal from the study, incomplete data, failure of study participants to undergo CMR (e.g. due to claustrophobia in randomised patients), problems with data acquisition and/or drop-out in up to 10% of participants, in line with our recent experience in clinical CMR studies.

3.6 Study schedule

Initial study assessments will be completed on day 1 where possible although assessments may be completed on different days to accommodate scheduling if necessary. Adjunctive

functional tests will take place during the initial angiogram. In the optional CMR sub-study, the CMR scan will be performed subsequently and typically on a planned elective basis. The CMR scan should be performed within 6 weeks of the invasive angiogram.

GJNH Cardiology Ward

Standard care checks

Screening inclusion/exclusion criteria

Health status questionnaires

Vital sign observations (heart rate and rhythm, blood pressure)

ECG

Physical examination

Height and weight

Standard care bloods (Complete blood count, Blood chemistry i.e. U&E)

Medical history, including cardiovascular risk scores (ASSIGN, JBS3)

Drug therapy

GJNH Catheter laboratory/Hairmyres Catheter laboratory: Baseline (Day 0)

Informed consent

Coronary angiogram, guidewire-based functional tests (FFR, CFR, IMR, etc)

Coronary reactivity testing with intra-coronary acetylcholine

The angiogram and invasive testing of coronary functionality will be performed as part of standard care

Blood samples (30 ml) for central laboratory test of circulating molecules: ET-1, ICAM, VCAM, p-selectin, Il-6, endothelial microparticles, glycaemic status (blood glucose, HbA1c, lipids), metabolomics, hs-troponin, hs-CRP and NT-proBNP. The buffy coat will be recovered

for analysis of DNA. Surplus blood will be stored for future analysis. These samples will be batched and analysed when additional funding becomes available.

Peripheral endothelial function assessment non-invasive assessment with the tongue videomicroscopy

The clinical data generated from the invasive procedure will be collected and recorded by research staff into the registry database.

Randomisation

T 1 – up to 6 weeks: study procedures

Cor-CMR sub-study (optional): contrast-enhanced stress perfusion CMR in those participants who have given informed consent to participate in the Cor-CMR sub-study

Drug therapy

Peripheral arteriolar function assessment using gluteal skin biopsy

T 6 months: Follow-up (hospital visit, where feasible)

Follow-up contact (by telephone initially or by letter or clinic review if required) Health status assessment (EQ5D, Seattle angina questionnaire, BIPQ, PHQ4, TSQM)

Drug therapy (including reason for self-cessation)

Lifestyle factors (diet, smoking, weight, exercise)

Urine sample (vasoactive drug metabolites) – concordance with therapy

Adverse events evaluation and reporting (including for health economics)

Up to 2 years (long-term follow-up using electronic records)

Blood samples (30 ml) at 1 year for central laboratory test of ET-1, ICAM, VCAM, p-selectin, Il-6, endothelial microparticles, glycaemic status (blood glucose, HbA1c), metabolomics, hs-troponin, hs-CRP and NT-proBNP.

Follow-up contact (by telephone initially or by letter or clinic review if required) with health status assessment (EQ5D, Seattle angina questionnaire)

Drug therapy

Adverse events evaluation and reporting (including for health economics)

Electronic case record linkage for adverse events (rehospitalisation and/or death) based on the Community Health Index or NHS number; no patient contact. The longer term follow-up duration will be from 3 years + for all participants. It is anticipated that de novo research grants will support this research in the longer term

3.7 Assessments and Procedures

Clinicians will be required to disclose their treatment plan after the coronary angiogram but before physiological testing thus the plan cannot be influenced by knowledge of the randomised strategy for their patient. The results of the diagnostic tests of microvascular function will be blinded in a 1:1 randomised fashion following the initial invasive procedure with functional tests. Blinding will include obscuring the display of the haemodynamic console from the clinical team who will acquire the coronary thermodilution data without seeing the results. These will be recorded and quality assured by the catheterization laboratory physiologist. Complete blinding will be ensured with the use of a second cardiologist (e.g. Dr Tom Ford) to supervise intra-coronary ACh whilst monitoring the

angiogram for visual display of coronary diameter and ECG during which time the attending clinician is blinded.

Given the open-label nature of the enrolment and group allocation (by random assignment), the decisions of the attending cardiologists during usual care are susceptible to overt and unmeasured bias. In order to mitigate for this possibility, an expert panel of up to 9 NHS cardiologists independent of the interventional cardiology service will be established in order to prospectively review a de-identified medical summary of the participants in the trial and registry. The medical summary will include the clinical history, test results (prior to & during angiography) and the angiogram findings. The deidentified angiogram will be made available for review. Each panel member will separately record their initial and final diagnoses and management plans using the study template (Appendix 2). This 'virtual' information will not be used to inform actual patient care. At the end of the study, the panel member decisions will be associated with the results of the coronary artery function tests and the reclassification rates by the panel members (individual & majority consensus) will be compared with the actual decisions by the attending cardiologist. These data will be associated with the other outcomes in the database. The anonymised medical summary and angiogram will have a unique study number with no other identifiers. The material will be made available by secure on-line access provided by the Glasgow Clinical Trials Unit or by packs prepared by the research team.

The results are intended to classify patients according to distinct endotypes (appendix 4) enabling the attending clinician to personalise the patients treatment according to contemporary guidelines and available clinical evidence. Since abnormalities in coronary function may lead to abnormalities in LV end-diastolic pressure, this will also be measured. In patients in whom a recent (< 3 months) assessment of LV function is lacking then left ventriculography will be performed. The pilot will test all aspects of the protocol, including

enrolment and compliance with the randomisation intervention. Seattle Angina scores will be prospectively assessed. Any learning from the internal pilot will be used to optimise the multicentre trial and all of the data from the pilot and main trial will be used to maximise impact. The overall aim is to fully assess a diagnostic strategy based on invasive physiology to stratify management, as a potential alternative to routine invasive management. The strategy is pragmatic and disruptive with respect to current standards of care, yet eminently transferrable to the NHS.

The data for each subject will be coded, de-identified and analysed in random order. The results of the standard CMR scan will be reported (i.e. LV mass, function, scar revealed by late gadolinium enhancement), but the results of the stress perfusion testing will not be disclosed since the coronary function tests are the standard of care used for decision making. The stress perfusion MRI data will be analysed and archived and associated with other relevant clinical data, including coronary function, health status and outcome in the longer term. The MRI analyses may involve sharing de-identified data with external collaborators, e.g. Drs. Andrew Arai and Li Yueh-Hsu in the Laboratory of Cardiovascular MRI, US National Institutes of Health.

3.7.1 Primary outcome analysis

3.7.1.1

The primary efficacy variable will be chest pain as measured by the Seattle angina score at 6 months, which will be compared between groups using a linear regression model, adjusting for the baseline score.

3.7.1.2

SAEs RELEVANT FOR EFFICACY AND SAFETY ANALYSES

Clinical event committee (CEC)

A CEC will be established to provide independent adjudication of serious adverse events using de-identified clinical information to be provided by the Chief Investigator/Clinical Trials Unit. The committee will be independent and include at least 3 cardiologists who are independent of the study. A CEC charter will be established with standardised definitions for clinical endpoints according to the "Standardised definitions for endpoint events in cardiovascular trials' (FDA, Hicks et al JACC 2015) and the "Third Universal Definition of Myocardial Infarction" (100).

CEC assessments and reporting will take place prospectively as the study progresses.

Definitions

1) Major Adverse Cardiac and Cerebrovascular Events (Cardiovascular MACCE)

Cardiovascular death, non-fatal myocardial infarction, hospitalisation for heart failure, non-fatal stroke or transient ischaemic attack and resuscitated cardiac arrest or implantable cardiac device. These are spontaneous adverse cardiovascular events.

PCI and CABG are adverse events are dependent on medical decisions and are not spontaneous. A second MACCE outcome measure with revascularisation is also pre-defined.

2) Major Adverse Cardiac Events (Cardiac MACE)

- defined as 'cardiac death, non-fatal MI or hospitalisation for heart failure'. The cardiac MACE will be considered for all MIs and also for MACE with spontaneous MI only (i.e. not Type 4 or Type 5 MI).

3) Failed medical management

- defined as cardiac MACE or coronary revascularisation.

4) MI associated with revascularisation procedures (types 4 and 5) (107)

Type 4a: Myocardial infarction related to percutaneous coronary intervention (PCI).

Myocardial infarction associated with PCI is arbitrarily defined by elevation of cardiac troponin values >5 x 99th percentile URL in patients with normal baseline values ≤99th percentile URL) or a rise of cardiac troponin values >20% if the baseline values are elevated and are stable or falling. In addition, either (i) symptoms suggestive of myocardial ischaemia, or (ii) new ischaemic ECG changes or new LBBB, or (iii) angiographic loss of patency of a major coronary artery or a side branch or persistent slow or no-flow or embolisation, or (iv) imaging demonstration of new loss of viable myocardium or new regional wall motion abnormality are required.

Type 4b: Myocardial infarction related to stent thrombosis

Myocardial infarction associated with stent thrombosis is detected by coronary angiography or autopsy in the setting of myocardial ischaemia and with a rise and/or fall of cardiac biomarkers values with at least one value above the 99th percentile URL.

Type 5: Myocardial infarction related to coronary artery bypass graft

Myocardial infarction associated with CABG is arbitrarily defined by elevation of cardiac biomarker values >10 x 99th percentile URL in patients with normal baseline cardiac troponin values ≤ 99th percentile URL. In addition, either (i) new pathological Q waves or new LBBB, or (ii) angiographic documented new graft or new native coronary artery occlusion, or (iii) imaging evidence of new loss of viable myocardium or new regional wall motion abnormality.

Other SAEs for the CEC charter: RECURRENT MI will be defined according to the Universal Definition of MI (107) and will involve ischaemic symptoms (e.g. chest pain) > 20 min with new ECG changes (new Q waves and/or ST segment elevation >0.1mV in 2 leads for > 30 min), a cardiac biomarker elevation (>99th centile upper reference limit). For patients who die, new chest pain or ST elevation would fulfil the criteria for MI. CEREBROVASCULAR EVENTS: TRANSIENT ISCHAEMIC ATTACK (TIA) is any focal neurological deficit with sudden onset that resolves within 24 h. A neurology review should be obtained wherever possible. STROKE is any rapidly developing clinical signs of focal (at times global) disturbance of cerebral function, lasting >24 hours or leading to death with no apparent cause other than a vascular origin (108). HEART FAILURE: Killip's classification will be used for heart failure following acute MI (109); Heart failure after discharge will be defined as a hospital admission with NYHA class III or IV heart failure and intravenous diuretic therapy or an increase in oral diuretics. SAEs obtained through Information and Statistics Division (ISD) of NHS Scotland and the Clinical Practice Research Datalink (CPRD) with the NHS number will be based on ICD-10 codes. Serious adverse events and those of interest (i.e. bleeding) will be collected by the research team (clinical research fellow, research nurse). Details of the event will be added to the eCRF..

Assessment of adverse events

All adverse events must be assessed for seriousness. SAEs must be assessed for causality, expectedness and severity and notified to the Sponsor. This is the responsibility of the CI or designee. SAEs that are potentially relevant to the designated secondary health outcomes will be assessed by the CEC.

3.7.2 Secondary outcomes

Feasibility

Rates of Enrolment, drop-out, completion of the diagnostic protocol,

Integrity of blinding in the catheter laboratory

Rates of cross-over between groups, adherence with medication during follow-up

Safety

Safety of coronary function tests

Diagnostic utility

To assess impact of disclosure of the coronary function test results on the diagnosis and the certainty of the diagnosis (diagnostic utility) (Appendix 2),

The clinician's diagnosis i.e. coronary heart disease, angina due to coronary heart disease, angina due to a disorder of coronary function e.g. microvascular angina, vasospastic angina, will be assessed for certainty (yes/no vs unlikely/probable in the primary analysis) and frequency (yes/probable vs unlikely/no). A recent example of this approach is the SCOT-HEART trial.

Clinical utility

To assess impact of disclosure of the coronary function test results on clinical management (including treatment and investigations) (Appendix 2),

Comparison of health status: Rose angina, Seattle angina scores (4 components), EQ5D health status, Illness perception, treatment satisfaction, clinical anxiety and depression (including comparison with non-randomised registry control group with obstructive CAD).

To assess the relationships between cardiovascular risk factors, reflected by validated risk scores (e.g. ASSIGN, JBS3), and parameters of coronary function, in medically managed patients.

NHS resource utilisation including primary and secondary care costs for tests, procedures and out-patient visits, and medicines

N-terminal prohormone brain natriuretic peptide (NT-proBNP), a surrogate outcome of prognosis

Cardiovascular science

Microvessel density in the tongue

Myocardial perfusion by quantitative stress CMR (optional sub-study).

Small arteriolar vasoactive function (optional sub-study)

Health outcomes

3.7.2.1 MACCE including coronary revascularisation

3.7.2.2 MACE including coronary revascularisation

3.7.2.3 Failed medical management

3.7.3.4 All-cause mortality

3.7.2.5 All-cause death or heart failure hospitalisation

3.7.2.6 NT-proBNP concentration at 12 months

3.7.3.7 Within-subject change in NT-proBNP concentration at 12 months from baseline

3.7.2.8 EQ-5D at 12 months

3.7.3.9 Within-subject change in EQ-5D at 12 months from baseline

3.7.2.10 Seattle angina score at 12 months

3.7.3.11 Within-subject change in Seattle angina score at 12 months from baseline

3.7.3.12 CMR sub-study outcomes

Stress testing with gadolinium contrast-enhanced MRI will be performed at 1.5 Tesla within 6 weeks of the invasive coronary angiogram.

MRI outcome definitions

First pass MVO

The extent of the first pass perfusion deficit at rest (i.e. first pass MVO) will be quantified in a summative manner to estimate the number of segments involved (82,83).

Table – Qualitative report parameters by CMR observer.

**Stress CMR clinician read-out criteria**	**Read-out parameters**
Perfusion abnormality	Yes / No
Characteristics of perfusion abnormality	Segments affected (AHA classification)
	Summed stress score (perfusion)
	Circumferential extent (degrees)
	Transmurality (0 - <25%; 25 - <50%; 50 - <75; 75 - 100%)
	Temporal pattern (qualitative comments: immediate, gradual, sustained)
	Coronary artery attribution (one or more epicardial arteries)

Scan quality	Likert ordinal scale
Diagnostic confidence	Ordinal scale (from low to high confidence)<br><br>Rating: non-diagnostic → diagnostic
Reproducibility*	Intra- and inter-rater reliability<br><br>Intra-class correlation coefficient

*Test-retest reliability for stress perfusion CMR will be explored should ethics committee approval be granted for repeated stress CMR in the same individual.

Quantitative perfusion CMR

Myocardial perfusion reserve index (MPRI; global, subendocardial, subepicardial, segmental);

Summed stress score (perfusion);

Myocardial blood flow (ml/g tissue);

Other novel parameters in exploratory analyses e.g. first-pass perfusion kinetics.

Atrial dimensions

Maximum LA volume (LAVmax) is defined as the volume at end-systole, before the mitral valve opens. Minimum LA volume (LAVmin) is defined as volume at end-diastole, after the mitral value closes. Pre-atrial contraction LA volume (LAVPreA) is defined as volume before the initiation of atrial contraction. LA volume index (LAVI) is indexed by body surface area.

Total LA emptying fraction (LAEF) was calculated as 100×(LAVmax – LAVmin) / LAVmax. Passive LAEF as 100 × (LAVmax – LAVpre-a) / LAVmax, and the active LAEF as 100 ×(LAVpre-a – LAVmin) / LAVpre-a.

Left ventricular strain

Circumferential, radial, longitudinal strains

Aortic stiffness

Aortic distensibility = (aortic area max. – aortic area min) / Aortic area min x pulse pressure

Myocardial T1 relaxation time and extra-cellular volume (ECV)

Myocardial longitudinal relaxation times (T1, ms) are influenced by tissue water content and pathology. A T1 mapping method will be used, e.g. MOLLI, as outlined in the MRI guideline. The time interval for the T1 map acquisitions will be recorded after the gadolinium contrast bolus. Native T1 (ms) and ECV will be calculated from regions of interest using dedicated software e.g. Medis QMass (Leiden, NL). The hematocrit will be used to calculate ECV, or a 'synthetic' ECV will be estimated according to the method described by Treibel et al (JACC CV Imaging 2016).

Infarct size

The presence of myocardial infarction will be established based on abnormalities in cine wall motion, rest first-pass myocardial perfusion, and late gadolinium enhancement imaging. Late gadolinium enhancement should be confirmed on both the short and long axis acquisitions, including with repeated MRI with change in the phase-encode direction in order to assess for and exclude artefacts. The myocardial mass of late gadolinium (grams) will be quantified by a semi-automatic detection method e.g. the full width half maximum technique. We have

adopted this definition in line with current standard practice in many MRI laboratories worldwide (74-77).

Tissue fibrosis with a non-ischaemic pattern will also be quantified.

Extracellular volume

Myocardial extracellular volume in regions of interest will be estimated using T1 mapping before and 15 min after gadolinium contrast administration.

3.7.3 MRI acquisition

The MRI scan will include cine MRI, T1-maps, strain encoded MRI, and contrast enhanced imaging including first pass perfusion during pharmacological stress with adenosine and then at rest. Microvascular obstruction and late gadolinium enhancement imaging will be performed 10 - 15 min after contrast administration. The scan parameters will be specified in a Standard Operating Procedure. Intravenous access will be needed for contrast infusion.

Patient preparation

A CMR checklist should be completed before the scan. See CMR guideline.

MRI protocol

MRI will be performed on 1.5 Tesla scanner with a standard (e.g. 8-element) phased array cardiac surface coil.

The MRI protocol includes:

- free breathing localisers including sagittal, coronal and axial acquisitions,

- T1-weighted MRI scans with a left ventricular short axis (SAX) (base, mid, distal), and one long axis view (vertical long axis, horizontal long axis and/or 3 chamber view),
- strain-encoded acquisitions at the same positions,
- stress perfusion imaging of the first pass of gadolinium contrast,
- cine-MRI for LV mass and function
- rest perfusion imaging,
- aortic stiffness assessment (phase contrast and/or cine-SSFP),
- late (10 - 15 minutes) gadolinium enhancement MRI acquired with a short axis LV stack and at least one long axis view.
- post-contrast T1 mapping.

Images will be acquired using the same slice positions. If a recent full blood count is not available then a blood test will be obtained before the scan in order to measure extracellular volume.

MRI scan imaging parameters

Cardiac mass and function will be assessed using steady-state free precession (SSFP) cine breath-hold sequences (with parallel imaging acceleration). The heart will be imaged in multiple parallel short-axis (SAX) planes 7-mm thick separated by 3-mm gaps, equating to approximately 10 slices and 30 cardiac phases.

T1 imaging will be planned based on equally spaced parallel short axis slices with the 2 outermost slices (mitral annular level and apex) discarded to retain the standard 3 left

ventricular basal, mid and apex slices ('3 of 5' technique), taking care to ensure the left ventricular outflow tract is excluded from the basal slice. Additional imaging may be performed e.g. valve flow study, as clinically appropriate.

Contrast-enhanced MRI

Stress perfusion CMR

An automated pump injector (e.g. Medrad) will be used for I.V. injection of gadolinium (Gadovist, Bayer) administered at an approximate rate of 0.035 mmol/kg at a rate of 5 ml per second followed by a 20 ml flush. A CMR compatible blood pressure monitor should be available.

First-pass perfusion at rest for 'wash-in' MVO quantification will be performed with an appropriate pulse sequence and run simultaneously with the contrast injection.

Late gadolinium enhancement

LATE MVO and scar will be imaged 10-15 minutes after intravenous Gadovist contrast administration using for example motion-corrected T1-weighted phase-sensitive inversion recovery radiofrequency pulse sequence. Alternate heart beat acquisition will be run (unless HR<55). Phase-sensitive inversion recovery MRI techniques reduce variability associated with myocardial nulling which is required for late gadolinium enhancement imaging of infarct vs. unaffected myocardium (74). If a phase-sensitive protocol is not used, a modified Look-Locker inversion time scout will be performed prior to using an inversion recovery turbo gradient echo sequence. Phase swaps will be performed where appropriate to rule out artefact. A single shot technique or navigated late gadolinium enhancement MRI will be used as an option for poor breath holders.

Typical imaging parameters are included in the MRI guideline.

CMR core laboratory image analysis

A clinical report should be issued for each CMR scan in line with local standards of clinical care. Since the stress perfusion CMR is not standard of care and is a research activity these results will not be reported. The CMR analyses will be conducted in line with local standard operating procedures.

CMR Core Laboratory (PIs Berry, Radjenovic): Each CMR scan will be logged and assessed for quality (poor, acceptable, good) and feedback will be provided to the site, as appropriate. The scans will be reported blind to all of the other clinical data, including the catheter laboratory data, treatment, and health status. The scans will be prospectively analysed by cardiologists and radiographers with expertise in CMR. Left ventricular mass and function and infarct characteristics will be analysed by trained staff. All of these data will be reviewed and approved by the CMR cardiologist (e.g. Prof Berry, Dr Corcoran) and quantitative analyses will be assured by Dr Radjenovic. Therefore, all scans will be reviewed by at least 2 trained observers. CMR measurements including LV dimensions, LVEF, T1 maps, strain, myocardial perfusion, scar, aortic distensiblity, will be prospectively analysed and recorded in a database.

MRI analyses will be performed on dedicated workstations with customised software (e.g. QMASS, Medis, Leiden). The 'industry-standard' software enables semi-automated standardised thresholding and border-delineation of regions-of-interest (ROI). User-involvement will be retained to adjust endocardial/epicardial borders (e.g. exclude blood pool) and occasional image artefacts. This software is an essential part of a core laboratory approach. For paired measurements for secondary outcomes, the baseline and follow-up MRI scans will be analysed together (side-by-side). This approach will ensure standardised settings and measurements for the baseline and follow-up scans.

Analysis of first pass contrast CMR: Using the modified ACC/AHA 16-segment nomenclature, segmental first pass will be interpreted as normal or abnormal. Each segmental abnormality will be scored on the basis of the transmural extent of the perfusion defect (0 = no defect, 1 = 1% to 50%, 2 = 51% to 100%). The apical cap (segment 17) will not be assessed because of the short-axis acquisition, so this segment will be treated as missing (i.e. 16 segment model). A perfusion defect will be deemed relevant only if it persists beyond peak myocardial enhancement. In an exploratory analysis, since non-transmural perfusion defects can be discriminated with high spatial resolution MRI, the myocardium will be divided into endocardial and epicardial segments thus resulting in 32 segments in total. The quantitative analyses of myocardial perfusion will be directed by Dr Radjenovic.

Quality assurance

There will be a continuous quality assurance process throughout the trial with feedback to sites on MRI scan quality. An NHS medical physicist from the lead site will assess and support the optimisation of MRI scan acquisition and image quality. An assessment of MRI data quality will be obtained from each site before enrolment of study participants, including of a T1 phantom for calibration, wherever possible.

3.7.4 Coronary angiogram

Importance of coronary vascular function

Coronary blood flow parameters can be measured objectively based on coronary angiography and the TIMI coronary flow grade.

Coronary angiogram acquisition

The coronary angiogram is the standard of care assessment for the presence of coronary artery disease.

Angiographic views: A comprehensive angiogram is anticipated based on contemporary standards, and orthogonal views of coronary lesion(s) should be obtained during standard care.

Coronary angiogram analysis

TIMI coronary flow grade[137]

TIMI Coronary Flow Grade	Description
0	No flow
1	Minimal flow past obstruction
2	Slow (but complete) filling and slow clearance
3	Normal flow and clearance

The TIME flow grade is straightforward to evaluate in the catheter laboratory and is independently predictive of prognosis.

Coronary angiography also provides other information on coronary blood flow and myocardial perfusion. For example, the TIMI blush grade provides an ordinal score for contrast washout at the end of each angiogram. The TIMI blush grade is also predictive of prognosis.

Tissue myocardial perfusion (blush) grade[138,139]

Grade	Description
0	No myocardial blush

Grade	Description
1	Minimal blush and very slow clearing (e.g. present at beginning of next cine)
2	Good blush with slow clearing of myocardial contrast (present at end of cine but gone at beginning of next)
3	Good blush and normal clearing (i.e. gone by end of cine)

TIMI frame count [140]

The TIMI frame count is a simple objective continuous variable index of coronary blood flow, representing the amount of time (in frames) for contrast dye to reach a standardised distal landmark, corrected for vessel length. The corrected TIMI frame count is also predictive of prognosis.

Method: Corrected TIMI frame count (CTFC, normal < 27 frames). The CTFC is the number of cine frames required for contrast to first reach standardized distal coronary landmarks in the culprit artery and is measured with a frame counter on a cine viewer. A frame count of 100, a value that is the 99th percentile of patent vessels, is imputed to an occluded vessel. CTFC is a measure of time, and data will convert when necessary according to film speed (e.g. 30 frames/s). The CTFC will be divided by 30 to calculate the transit time for dye to traverse the length of the artery to the landmark in seconds and multiplied by 1000 to calculate the time in milliseconds. This will be used along with the heart rate to calculate the fraction of a cardiac cycle required for dye to traverse the artery: fraction of cardiac cycle (CTFC/30 seconds)/(60s/heart rate). Calculation of the fraction of a cardiac cycle required for dye to traverse the culprit artery normalizes the CTFC for heart rate.

Angiographic measurements

Angiography Core Lab (PIs Berry & McEntegart): Each coronary angiogram will be de-identified and analysed in the Glasgow Core Laboratory based in the Golden Jubilee National Hospital. The angiogram will be analysed by trained observers in the Angiography Core Laboratory of the Golden Jubilee National Hospital under the direct supervision of Professors Berry and McEntegart who have considerable experience of angiographic core lab analyses[83]. Dedicated angiographic software and image review workstations will be used.

Quality assurance of angiograms: Investigator training will be essential to ensure optimal angiographic data. A standard operating procedure for coronary angiography will be used for imaging analysis.

3.7.4.1 Invasive assessment of coronary physiology

Fractional flow reserve (FFR)

FFR is a measure of the functional severity as assessed by the ratio of distal coronary pressure to aortic pressure measured during coronary hyperemia [1].

Method: The cardiologist should pass the diagnostic wire across the target coronary stenosis. The pressure wire (Certus, St Jude Medical, Uppsala) will be calibrated initially to ensure standardised measurements and when positioned at the distal end of the guide catheter the pressure wire recording will be equalized with the aortic pressure. The wire is then passed into the coronary artery of interest and advanced at least 6 cm distal to the coronary stenosis using standard techniques. Once the marker is appropriately positioned and after an initial 2 minute rest period, an intravenous infusion of adenosine (140 mcg/kg/min–210 mcg/kg/min) via a central vein or large antecubital vein is started to establish coronary hyperemia. Typical changes in blood pressure (i.e. fall in systolic pressure

>10%), heart rate (i.e. rise in heart rate >20%) and symptoms will be recorded prospectively to confirm a hemodynamic response to adenosine during a period of at least 2 minutes. When there is an inadequate response with the standard dose of adenosine (140 mcg/kg/min) then the dose can be increased up to 210 mcg/kg/min in order to best ensure maximal hyperaemia. If intravenous adenosine is not tolerated then intracoronary adenosine could be administered or FFR will be not be recorded and this will be noted in the Case Report Form. An FFR <0.80 is taken as abnormal, and a grey-zone FFR is taken between 0.75 – 0.82.

Coronary flow reserve (CFR)

CFR represents the vasodilator capacity of the coronary vascular bed during hyperaemia and is measured by indicator thermodilution.

Method: A bolus of saline (i.e. 3 mL) at room temperature injected through the guide catheter will mix with antegrade coronary blood flow at body temperature, causing a transient reduction in temperature that is measured by the thermistor, located 3 cm from the distal end of the guidewire. The thermodilution curve is reflected by a transit time. The mean transit time for three saline injections is calculated both at rest and during pharmacological hyperaemia. CFR is calculated as resting mean transit time divided by hyperemic mean transit time – in this study CFR <2.0 is considered indicative of abnormal coronary reactivity.

Index of microvascular resistance (IMR)

Myocardial resistance is mainly determined by the microcirculation. IMR is a coronary guidewire-based measure of coronary microvascular function. It is a measure of distal coronary pressure divided by coronary flow - the apparent IMR is calculated by multiplying the distal coronary pressure by the mean transit time of a 3 ml bolus of saline at room

temperature during coronary hyperaemia induced by intravenous adenosine. Pressure and temperature are measured simultaneously since the pressure-sensor and thermistor are located at the same point on the coronary guidewire (3 cm from the distal end). IMR *>25 will be considered* abnormal indicative of abnormal coronary reactivity.[65-75]

Resistance reserve ratio (RRR)

The resistance reserve ratio (RRR) is calculated as a measure of the vasodilatory capacity of the microcirculation expressed as the ratio of basal resistance / IMR [75]

Absolute coronary blood flow and resistance

Using the same principle of CFR measurement using thermodilution, during a steady infusion of saline (20 – 30 ml/min) administered via a dedicated infusion catheter e.g. Rayflow (Hexacath, France), hyperaemia can be achieved such that FFR, as well as absolute coronary blood flow (ml/min) and resistance (mmHg.min.ml^{-1} or Wood units) can be measured at the same time. Absolute coronary blood flow = infusion flow rate x (proximal infusion temperature (guide catheter) /distal infusion temperature (from the sensor) x 1.08 (heat constant).[76]

3.7.4.2 Adjunctive invasive assessments of coronary endothelial function

Intracoronary acetylcholine (IC-ACh) is a muscarinic receptor agonist that will be administered at incremental concentrations (10^{-6}M, 10^{-5}M, 10^{-4}M; 2 min each) via the diagnostic catheter. The normal response to acetylcholine is vasodilation, however if endothelial dysfunction is present vasoconstriction may occur.[2] ACh will be provided in packs by the Pharmacy Production Unit in such a way that the reconstituted solutions will be easily available for immediate administration by the attending cardiologist in the catheter laboratory. Coronary artery diameter will be assessed using cine angiography before the first infusion and 2 minutes after each infusion unless the threshold is met. A vasoconstrictor

response with reduction in diameter of >20% after intracoronary infusion of acetylcholine will be deemed functionally significant.[3] If all doses of infusions are negative for spasm, a final provocative challenge using 100mcg of ACh will be given (5.5ml of 10^{-4}M over 20 seconds) into the LAD only. Finally, coronary diameter will be assessed following an IC bolus of 300 µg (3 ml) of GTN, a non-endothelium-dependent vasodilator.

Measurement of coronary artery dimensions will be made using quantitative coronary analysis software e.g. QAngio, Medis, Leiden, NL, with the measurements specified at the location of the greatest change in the diameter of the artery. Coronary artery diameter change (% from baseline) will be measured in response to both acetylcholine and glyceryl trinitrate. Coronary artery spasm will be defined as focal or diffuse. The protocol is in line with our recent favourable experience in the RIC-COR study: https://clinicaltrials.gov/ct2/show/NCT02666235

3.7.5 Drug therapy

Medications used for treatment of cardiovascular disorders, including primary and secondary prevention therapies will be recorded at baseline, 1 month, 6 months, 12 months and annually thereafter.

Medication use will be optimized according to contemporary guidelines of the European Society of Cardiology.

3.7.6 Biochemical assessments

Study blood samples (30ml) will be obtained in the catheterisation laboratory at the time of the diagnostic angiogram. Central laboratory testing of glycaemic status (plasma glucose, HBA1c, lipids), hs-Troponin, hsCRP, NT-pro BNP, plasma serotonin will be carried out together with measurement of endothelial cytokines (ET-1, ICAM, VCAM, p-selectin; ELISA).

Following these analyses, the residual blood will be stored for future analyses of novel circulating substances that may be implicated in the pathophysiology of abnormal coronary function. These analyses would be the subject of a separate ethics application. Blood will be collected at baseline, 6 and 12 months to provide analyses of the within-subject change in these parameters (101)). The biochemical analyses will be performed in a central laboratory in the Glasgow. The biochemical measurements are justified since their measurement accuracy and coefficient of variation are lower than MRI therefore the biochemical parameters will provide independent measurements of left ventricular remodeling and function to complement the MRI data.

Local hospital blood sample handling

Biochemistry Core Lab (PI Sattar) Serum and plasma samples will be stored at -80ºC in the Glasgow Biorepository until batch analysis at the end of the study. NT-proBNP (101) will be measured using standard assays. For NT-proBNP, the low control coefficient of variation was 6.7% and the high control coefficient of variation was 4.9%. NT-proBNP was measured in a research laboratory using an electro-chemiluminescence method (e411, Roche) and the manufacturers' calibrators and quality control material. The limit of detection NT-proBNP is 5 pg/ml, respectively. Long-term coefficient of variations of low and high controls are typically <5%, and were all within the manufacturers' range.

The blood test results will be provided to the Robertson Centre for Biostatistics, University of Glasgow.

3.7.6 Peripheral endothelial function assessment

It is plausible that the function of the coronary and peripheral microcirculation might be inter-related. Therefore we also wish to assess the peripheral (systemic) microcirculation to

allow us to make a preliminary correlation with the coronary function measurements. Using bedside sidestream dark field video microscopy (Microscan™, http://www.microvisionmedical.com), it is possible to non-invasively assess microvascular density and obtain a semi-quantitative measure of perfusion in peripheral tissues, such as the tongue. To date, this device has predominantly been used to assess peripheral microcirculatory function in patients with other vascular conditions such as septic shock. This device provides information on vascular density with a read-out segmented according to 'small' (<20 um), 'medium' and 'larger vessel' density and length, and a semi-quantitative metric of perfusion, the proportion of perfused vessels (PPV).

3.7.7 Peripheral arteriolar function assessment

Consecutive CorMicA study participants in Glasgow will be invited to participate in an optional vascular sub-study. Participation would involve having a gluteal skin biopsy in the Beardmore Health Sciences Research Centre in the Golden Jubilee National Hospital. Patients with or without a diagnosis of CMD/CMA will be enrolled, patients in who CMD/CMA has been excluded will serve as controls. Outpatient gluteal skin biopsies will be performed as per a Standard Operating Procedure. Patients will attend in the morning fasting from the night before. Informed consent will be affirmed. The gluteal biopsy will involve a sterile technique and use of local anaesthetic by a trained operator. The tissue will be placed in a sealed container and transferred to the vascular laboratories of the University of Glasgow. The patient will be invited to re-attend approximately 1 week later for a review of the wound and removal of the stiches.

The specific laboratory protocols for studies of vascular biology & pharmacology will be detailed in Standard Operating Procedures.

3.7.8 Health status: symptoms and quality of life

Angina symptoms will be assessed with the Seattle angina questionnaire

Quality of life: will be assessed using the EQ5D-5l QoL questionnaire (103). The study participant will complete the questionnaire with a member of the research team (as needed) at baseline and 6 months. Within-subject change will be calculated. Illness perception (Brief IPQ), anxiety/depression (PHQ4) , treatment satisfaction (TSQM), Functional status (DASI)[4] and activity levels (IPAQ-SF)[5] will be recorded similarly using the questionnaires (Appendix 7-11)

The questionnaire data will be uploaded and entered locally into the eCRF.

3.7.9 Health outcomes

Assessment of adverse events during follow-up

Health outcomes will include death, re-hospitalisation for cardiovascular events including recurrent MI, heart failure, and coronary revascularisation. Data on health outcomes will be collected after (but not including) the index procedure at baseline. The next assessment will take place at 12 months. Event reporting will be performed prospectively during the study and quality assurance will also take place prospectively.

Participants will not be contacted after the 12 month assessment. After 12 months, electronic case records will be assessed by research staff. Electronic record linkage will be performed using the Community Health Index (CHI) number in Scotland and NHS number in England and Wales. The record linkage plan will be implemented by the Information and Statistics Division (ISD), and Clinical Practice Research Datalink (CPRD) linked to the Information Centre in England at the end of the study (3 years +). These are quality assured NHS systems made possible by electronic registration of all deaths and

hospitalisations (and their causes) which have been used widely, including for studies including publications by the trial statistician (105). Participants will be invited to give permission for life-long electronic case record linkage.

NHS resource utilization will also be assessed by identifying outpatient and inpatient visits in primary and secondary care, procedures, and medicine use. These data will be used to support an exploratory health economic analysis in order to inform the rationale and future design of a more definitive clinical trial.

3.8 Other assessments that are not secondary outcome measurements

3.8.1 Haematology and clinical chemistry (usual care)

A complete blood count and U&E test for renal biochemistry will be performed at baseline as part of standard care in the hospital laboratory.

3.8.2 Electrocardiogram (ECG)

ECG acquisition

A 12-lead ECG will be acquired as part of routine care at the time of the invasive angiogram. The ECG will be acquired by trained cardiology staff using standard ECG recorders available in the cardiology department.

ECG analysis

A 12 lead ECG will be assessed according to standard methods. The ECG rate, rhythm, axis and conduction will be recorded.

3.8.3 Anthropometry

Height, weight, body mass index, body surface area will be calculated from the baseline data.

4. MONITORING OF ADVERSE EVENTS

4.1 Definitions of adverse events

Adverse Event (AE) – Any untoward medical occurrence in a subject, including occurrences which are not necessarily caused by or related to that product.

Adverse Reaction (AR) – Any untoward and unintended response in a subject to an investigational medicinal product which is related to any dose administered to that subject.

Serious Adverse Event (SAE) or Serious Adverse Reaction (SAR)

Any adverse event or adverse reaction that:

- results in death

- is life-threatening

- requires hospitalisation or prolongation of existing hospitalisation

- results in persistent or significant disability or incapacity

- consists of a congenital anomaly or birth defect

- is otherwise considered medically significant by the investigator

i.e. Important adverse events/ reactions that are not immediately life-threatening or do not result in death or hospitalisation but may jeopardise the subject or may require intervention to prevent one of the other outcomes listed in the definition above.

4.2 Assessment, recording and reporting of Adverse Events

All SAEs in relation to a study procedure, e.g. the CMR scan, will be notified to the Sponsor.

SAEs RELEVANT FOR EFFICACY AND SAFETY ANALYSES

Clinical event committee (CEC)

A CEC will be established to provide independent adjudication of serious adverse events using de-identified clinical information to be provided by the Chief Investigator/Clinical Trials Unit. The committee will be independent and include at least 3 cardiologists who are independent of the study. A CEC charter will be established with standardised definitions for clinical endpoints according to the "Standardised definitions for endpoint events in cardiovascular trials' (JACC 2015) and the "Third Universal Definition of Myocardial Infarction" (100).

CEC assessments and reporting will take place prospectively as the trial progresses.

4.3 Annual safety reporting

An annual safety report will be submitted to the REC as soon as is practicable and until the End of the Study.

5. STATISTICS AND DATA ANALYSIS PLAN

5.1 Statistical analysis plan

The CorMicA study will have a comprehensive Statistical Analysis Plan, which will govern all statistical aspects of the study, and will be authored by the Trial Statistician before any unblinded data is seen.

5.2 General considerations

Null hypothesis: there is no association between a diagnostic strategy involving invasive tests of coronary function and a change in health status, health outcomes or patient management.

Statistical approach: Statistical Analysis Plan (SAP) based on intention to treat principles in line with CONSORT guidelines. The analysis will focus on estimation of treatment effect differences with 95% CIs and p-values. All pre-specified secondary outcome analyses will be reported in study publications to further inform efficacy, safety, and cost-effectiveness.

Interim analyses: 6 months, 1 year, 2 years

5.3 Primary outcome variable

ALTERNATIVE HYPOTHESIS: There is no difference in within-subject change for chest discomfort as revealed by the Seattle angina questionnaire (at 6 months).

5.4 Secondary efficacy analysis

Continuous outcomes will be analysed where data are approximately normally distributed and where baseline levels are available for adjustment. Where this is not the case, two sample t-tests and corresponding confidence intervals will be used. Where data are clearly not normally distributed (e.g. laboratory variables) standard transformations will be applied to achieve approximate normality prior to analysis.

The purpose of the laboratory analyses is to provide mechanistic information on the pathways that may be causally implicated in abnormalities of coronary artery function and myocardial perfusion.

The purpose of measuring NT-proBNP and health status is to provide information on validated surrogate outcomes.

CLINICAL OUTCOMES will be presented with Kaplan-Meier time-to-event curves & compared where appropriate using log rank tests. The angiographic parameters will be correlated with clinical outcomes (88,89).

5.6 Software and statistical analysis

Clinical data will be made available to the data coordinating centre (the Robertson Centre for Biostatistics) through the web-based eCRF. The RCB is an NIHR-approved Clinical Trials Unit (Registration number 16). The statistical software to be used will be SAS and/or R and/or SPSS.

5.7 Sample size

SAMPLE SIZE: We plan an internal pilot study of 150 patients with 1:1 randomisation and a minimum follow-up duration of 6 months.

Power calculation and alternative hypothesis:

Power calculation: If 6 month outcomes can be obtained from a minimum of 108 patients, this trial will have 80% power to detect a mean between-group difference in within-subject change in Seattle Angina Questionnaire scores of 0.24 standard deviation (SD) units. The sample size accounts for potential drop-outs. This projected effect is consistent with other studies, for example, the observed difference in the change in SAQ score with Ranolazine (9.43±24.13; p=0.027) vs. placebo in patients with a reduced CFR. This is a small difference but relevant within the context that we anticipate not all patients will have their therapy changed as a result of disclosure. Using the coronary function data for the control (non-disclosure) group, we will carry out focused analyses of patients whose therapy would be altered due to disclosure of coronary function results based on the established diagnostic thresholds for these tests. For example, if therapy is altered in 50% of patients, the study will have 80% power to detect a difference of 0.34 SD units for these patients; if therapy is altered in 30% of patients, there will be 80% power to detect a between-group difference of 0.44 SD units for these patients. Allowing for incomplete data or loss to follow-up the sample size is 150. We will assess health outcomes in the longer term (i.e. ≥3 years) using e-record linkage using NHS and government records without contacting the participants.

Secondary 2.1 Re-classification of the initial diagnosis based on angiography alone (Table 5).The options are: 1) Angina due to obstructive CAD; 2) Microvascular angina – endothelial dysfunction; 3) Vasospastic angina – coronary spasm; 4) Vasospastic Angina – microvascular

spasm; 5) Non-anginal chest pain. A secondary analysis will be the between-group comparison of the change in diagnosis using logistic regression, adjusted for baseline factors associated with the likelihood of reclassification of the initial diagnosis.

Table - Sample size calculation: change in diagnosis (% of patients)		Power, %	Group size, n
Disclosed Group	Usual Care Group		
20	(a) 0.1	90	(b) 45
25	5	90	75
(a) (b) Approximately 0 - 1% reclassification; Calculation based on Fisher's Exact Test. Calculations by Dr. A McConnachie			

Power calculations for the assessment of reclassification of diagnoses based upon panel consensus are as follows: the participation of physicians in a study of microvascular angina may bias their assessment of clinical diagnoses. Pilot data suggest that surgeons classify approximately 80% of cases as having angina that is probably or definitely due to a disorder

of coronary function. We will have paired data for each assessment, showing the majority assessment of a panel of experts. We can only speculate as to the percentage of panel assessments that would indicate likely coronary dysfunction, but assuming this is 60% then the study with 150 paired responses will have greater than 99% power to detect the bias. If paired responses can only be obtained for only 120 patients, the power is still 98%.

Pilot Data: We have discussed these estimates with other cardiologist investigators and, based on our understanding of the literature and our experience in clinical practice, we believe that these projected effect sizes would potentially be clinically meaningful and credible.

6. STUDY CLOSURE / DEFINITION OF END OF STUDY

The study will end when one or more of the following situations applies:

The planned sample size has been achieved;

There is insufficient funding to support further recruitment, and no reasonable prospect of additional support being obtained;

New information makes it inappropriate to continue to enrol patients;

Recruitment is so poor that completion of the trial cannot reasonably be anticipated.

7. DATA HANDLING

7.1 Case Report Forms / Electronic Data Record

An electronic case report form (eCRF) will be used to collect study data. The eCRF will be developed by investigators in the University of Glasgow and access to the eCRF will be restricted, with only authorised site-specific personnel able to make entries or amendments to their patients' data. It is the investigator's responsibility to ensure completion and to review and approve all data captured in the eCRF.

All data handling procedures will be detailed in a Study Specific Data Management Plan. Data will be validated at the point of entry into the eCRF and at regular intervals during the study. Data discrepancies will be flagged to the study site and any data changes will be recorded in order to maintain a complete audit trail (reason for change, date change made, who made change).

7.3 Data Retention

To enable evaluations and/or audits from regulatory authorities, the investigator agrees to keep records, including the identity of all participating subjects (sufficient information to link records), all original signed informed consent forms, serious adverse event forms, source documents, and detailed records of treatment disposition in accordance with ICH GCP, local regulations, or as specified in the Clinical Study Agreement, whichever is longer. Data will be retained at the Data Centre for a minimum of 10 years after the end of the study.

8. STUDY MANAGEMENT

8.1 Routine management of the study

The study will be coordinated by the Study Management Group which will include those individuals responsible for the day-to-day management of the study including the Chief Investigator, Co-Investigators, Research Nurse and others as considered appropriate. The role of this group is to facilitate the progress of the study, ensure that the protocol is adhered to and take appropriate action to safeguard participants and the quality of the study itself.

8.2 Clinical endpoints committee

Clinical events identified as potentially relevant to the designated secondary health outcomes will be assessed by a Clinical Event Committee (CEC). The CEC will be independent of both the investigators and the funder/sponsor and will be blinded regarding any information relating to the randomisation group. The composition of the CEC will be determined by agreement with the sponsor.

9. STUDY MONITORING AND AUDITING

Study Monitoring will be conducted by monitors on behalf of NHS Golden Jubilee National Hospital Research Management Office. During monitoring assessments, Informed Consent Forms will be reviewed and source clinical data as appropriate.

10. PROTOCOL AMENDMENTS

Any change in the study protocol will require an amendment. Any proposed protocol amendments will be initiated by the CI following discussion with the Sponsor and any required amendment forms will be submitted to the regulatory authority, ethics committee and sponsor. The Sponsor will determine whether an amendment is non-substantial or substantial. All amended versions of the protocol will be signed by the CI and Sponsor representative. Following a major amendment, favourable opinion/approval must be sought from the original reviewing REC, and Research and Development (R&D) office. The Chief Investigator will be responsible for informing the Trial Management Group of all protocol amendments.

11. ETHICAL CONSIDERATIONS

11.1 Ethical conduct of the study

The study will be carried out in accordance with the World Medical Association Declaration of Helsinki (1964) and its revisions (Tokyo [1975], Venice [1983], Hong Kong [1989], South Africa [1996], Edinburgh [2000], Seoul [2008] and Fortaleza [2013]).

Favourable ethical opinion will be sought from West of Scotland Research Ethics Committee before patients are entered into this clinical trial. Patients will only be allowed to enter the study once they have provided written informed consent (Section 3.5.1).

The Chief Investigator and/or Sponsor will be responsible for updating the Ethics committee of any new information related to the study.

11.2 Informed consent

Written informed consent will be obtained from each trial participant when on the ward. When this is not possible, the Patient Information Sheet and Consent form may be sent to the patient by mail.

The Clinical Research Nurse or clinical investigator will explain the exact nature of the study in writing, provision of patient information sheet, and verbally. Trial participants will be informed that they are free to withdraw their consent from the study or study treatment at any time.

12. INSURANCE AND INDEMNITY

The CorMicA pilot study is sponsored by NHS Golden Jubilee National Hospital. The sponsor will be liable for negligent and non-negligent harm associated with the trial. NHS indemnity is provided under the Clinical Negligence and Other Risks Indemnity Scheme (CNORIS) in Scotland, and the equivalent scheme in England.

The NHS has a duty of care to patients treated, whether or not the patient is taking part in a clinical study, and the NHS remains liable for clinical negligence and other negligent harm to patients under its duty of care.

As this is a clinician-led study there are no arrangements for no-fault compensation.

13. FUNDING, PEER REVIEW AND PUBLIC INVOLVEMENT

13.1 Funding and peer review

The CorMicA pilot is enabled through the British Heart Foundation Centre of Research Excellence award of which Professor Berry is a Co-Investigator. The British Heart Foundation is an Eligible Funder.

Dr Tom Ford has been appointed as a BHF clinical research fellow for the CorMicA pilot study.

NHS Support is anticipated to be made available to the Golden Jubilee National Hospital.

13.2 Patient and public involvement

The design of this study has been informed by discussions with patients and their families in relation to improving what is known about angina in patients who do not have obstructive coronary disease.

14. ANNUAL REPORTS

Annual reports will be submitted to the ethics committee and sponsor with the first submitted one year after the date that all trial related approvals are in place.

15. DISSEMINATION OF FINDINGS

The study will be registered on the clinicaltrials.gov website.

It is anticipated that the results will be published in a peer reviewed journal.

16. REFERENCES

1. Bhatnagar P, Wickramasinghe K, Williams J, Rayner M, Townsend N. The epidemiology of cardiovascular disease in the UK 2014. Heart. 2015 Aug 1;101(15):1182-9. doi: 10.1136/heartjnl-2015-307516. Epub 2015 Jun 3. Review. PubMed PMID: 26041770; PubMed Central PMCID: PMC4515998.
2. Herrett E, Smeeth L, Walker L, Weston C, on behalf of the MINAP Investigators. The Myocardial Ischaemia National Audit Project (MINAP). Heart 2010;96(16):1264–7.
3. Townsend N, Bhatnagar P, Wilkins E, Wickramasinghe K, Rayner M (2015). Cardiovascular disease statistics, 2015. British Heart Foundation: London. https://www.bhf.org.uk/publications/statistics/cvd-stats-2015
4. Ludman P on behalf of the British Cardiovascular Intervention Society (BCIS). BCIS Audit Returns for Adult Interventional Procedures (Jan – Dec 2015). October 2016. http://www.bcis.org.uk/pages/page_box_contents.asp?pageid=840&navcatid=11
5. Patel MR, Peterson ED, Dai D, et al. Low diagnostic yield of elective coronary angiography. N Engl J Med 2010; 362:886-95.
6. Douglas PS, Patel MR, Bailey SR, Dai D, Kaltenbach L, Brindis RG, Messenger J, Peterson ED. Hospital variability in the rate of finding obstructive coronary artery disease at elective, diagnostic coronary angiography. J Am Coll Cardiol 2011;58:801–809.
7. Bradley S, Spertus J, Kennedy K et al. Patient Selection for Diagnostic Coronary Angiography and Hospital-Level Percutaneous Coronary Intervention Appropriateness Insights From the National Cardiovascular Data Registry. JAMA Intern Med. 2014;174(10):1630-1639.
8. Jespersen L, Hvelplund A, Abildstrom SZ, Pedersen F, Galatius S, Madsen JK, Jorgensen E, Kelbaek H, Prescott E. [6-8]. Eur Heart J 2012;33:734–744.
9. Cannon RO III, Epstein SE. “Microvascular angina” as a cause of chest pain with angiographically normal coronary arteries. Am J Cardiol 1988;61:1338e43.

10. Ong P, Athanasiadis A, Borgulya G, Mahrholdt H, Kaski JC, Sechtem U. High prevalence of a pathological response to acetylcholine testing in patients with stable angina pectoris and unobstructed coronary arteries. The ACOVA Study (Abnormal COronary VAsomotion in patients with stable angina and unobstructed coronary arteries). J Am Coll Cardiol 2012;59:655–662.
11. Ong P, Athanasiadis A, Borgulya G, Vokshi I, Bastiaenen R, Kubik S, Hill S, Schäufele T, Mahrholdt H, Kaski JC, Sechtem U. Clinical usefulness, angiographic characteristics, and safety evaluation of intracoronary acetylcholine provocation testing among 921 consecutive white patients with unobstructed coronary arteries. Circulation. 2014 Apr 29;129(17):1723-30.
12. Sara JD, Widmer RJ, Matsuzawa Y, Lennon RJ, Lerman LO, Lerman A. Prevalence of Coronary Microvascular Dysfunction Among Patients With Chest Pain and Nonobstructive Coronary Artery Disease. JACC Cardiovasc Interv. 2015 Sep;8(11):1445-53. doi: 10.1016/j.jcin.2015.06
13. Murthy VL, Naya M, Taqueti VR, Foster CR, Gaber M, Hainer J, Dorbala S, Blankstein R, Rimoldi O, Camici PG, Di Carli MF. Effects of sex on coronary microvascular dysfunction and cardiac outcomes. Circulation. 2014 Jun 17;129(24):2518-27.
14. Camici PG, Crea F. Coronary microvascular dysfunction. N Engl J Med 2007; 356:830–840.
15. Kaul S, Jayaweera AR. Myocardial capillaries and coronary flow reserve. J Am Coll Cardiol. 2008;52(17):1399-401.
16. Lanza GA, Crea F. Primary coronary microvascular dysfunction: clinical presentation, pathophysiology, and management. Circulation 2010;121:2317–2325.
17. Yilmaz A, Sechtem U. Angina pectoris in patients with normal coronary angiograms: current pathophysiological concepts and therapeutic options. Heart. 2012 Jul;98(13):1020-9.
18. Marzilli M, Merz CN, Boden WE, Bonow RO, Capozza PG, Chilian WM, DeMaria AN, Guarini G, Huqi A, Morrone D, Patel MR, Weintraub WS. Obstructive coronary atherosclerosis and ischemic heart disease: an elusive link! J Am Coll Cardiol. 2012 Sep 11;60(11):951-6.

19. Herrmann J, Kaski JC, Lerman A. Coronary microvascular dysfunction in the clinical setting: from mystery to reality. Eur Heart J. 2012 Nov;33(22):2771-83.
20. Camici PG, d'Amati G, Rimoldi O. Coronary microvascular dysfunction: mechanisms and functional assessment. Nat Rev Cardiol. 2015 Jan;12(1):48-62.
21. Sheikh AR, Wei J, Merz CDB, Beltrame JF. The Current State of Invasive Coronary Evaluation and Management of Patients With Angina and Nonobstructive Coronary Arteries. http://www.acc.org/latest-in-cardiology/articles/2016/05/26/08/31/the-current-state-of-invasive-coronary-evaluation-and-management-of-patients-with-angina-and-nonobstructive-coronary-arteries?w_nav=LC .
22. Marinescu MA, Löffler AI, Ouellette M, Smith L, Kramer CM, Bourque JM. Coronary microvascular dysfunction, microvascular angina, and treatment strategies. JACC Cardiovasc Imaging. 2015 Feb;8(2):210-20.
23. Pries AR, Badimon L, Bugiardini R, Camici PG, Dorobantu M, Duncker DJ, Escaned J, Koller A, Piek JJ, de Wit C. Coronary vascular regulation, remodelling, and collateralization: mechanisms and clinical implications on behalf of the working group on coronary pathophysiology and microcirculation. Eur Heart J. 2015 Jun 25. pii: ehv100. [Epub ahead of print] Review. PubMed PMID: 26112888.
24. Zeiher AM, Krause T, Schächinger V, Minners J, Moser E. Impaired endothelium-dependent vasodilation of coronary resistance vessels is associated with exercise-induced myocardial ischemia. Circulation. 1995 May 1;91(9):2345-52. PubMed PMID: 7729020.
25. Mohri M, Koyanagi M, Egashira K, Tagawa H, Ichiki T, Shimokawa H, Takeshita A. Angina pectoris caused by coronary microvascular spasm. Lancet. 1998 Apr 18;351(9110):1165-9.
26. De Bruyne B, HersbACh F, Pijls NH, Bartunek J, Bech JW, Heyndrickx GR, Gould KL, Wijns W. Abnormal epicardial coronary resistance in patients with diffuse atherosclerosis but "Normal" coronary angiography. Circulation. 2001 Nov 13;104(20):2401-6.

27. Task Force Members, Montalescot G, Sechtem U, Achenbach S, et al; ESC Committee for Practice Guidelines, Zamorano JL, Achenbach S, Baumgartner H, et al. 2013 ESC guidelines on the management of stable coronary artery disease: the Task Force on the management of stable coronary artery disease of the European Society of Cardiology. Eur Heart J. 2013 Oct;34(38):2949-3003. doi: 10.1093/eurheartj/eht296. Epub 2013 Aug 30. Erratum in: Eur Heart J. 2014 Sep 1;35(33):2260-1. PubMed PMID: 23996286.http://www.escardio.org/static_file/Escardio/Guidelines/publications/ANGINA2013_Stable_Coronary_Artery_Disease_web_addenda.pdf
28. Cooper A, Calvert N, Skinner J, Sawyer L, Sparrow, K, Timmis A, Turnbull N, Cotterell M, Hill D, Adams P, Ashcroft J, Clark L, Coulden R, Hemingway H, James C, Jarman H, Kendall J, Lewis P, Patel K, Smeeth L, Taylor J. (2010) Chest pain of recent onset: Assessment and diagnosis of recent onset chest pain or discomfort of suspected cardiac origin London: National Clinical Guideline Centre for Acute and Chronic Conditions. NICE-95.
29. Yanagisawa M, Kurihara H, Kimura S, Tomobe Y, Kobayashi M, Mitsui Y, Yazaki Y, Goto K, Masaki T. A novel potent vasoconstrictor peptide produced by vascular endothelial cells. Nature. 1988 Mar 31;332(6163):411-5.
30. Lüscher TF. Endothelin: systemic arterial and pulmonary effects of a new peptide with potent biologic properties. Am Rev Respir Dis. 1992 Nov;146(5 Pt 2):S56-60.
31. Davenport AP, Maguire JJ. Endothelin. Handb Exp Pharmacol. 2006;(176 Pt1):295-329.
32. Davenport AP, Maguire JJ. Pharmacology of renal endothelin receptors. Contrib Nephrol. 2011;172:1-17.
33. de Nucci G, Thomas R, D'Orleans-Juste P, Antunes E, Walder C, Warner TD, Vane JR. Pressor effects of circulating endothelin are limited by its removal in the pulmonary circulation and by the release of prostacyclin and endothelium-derived relaxing factor. Proc Natl Acad Sci U S A. 1988 Dec;85(24):9797-800.

34. Vanhoutte PM. Endothelium and control of vascular function. State of the Art lecture. Hypertension. 1989 Jun;13(6 Pt 2):658-67.
35. Ray SG, McMurray JJ, Morton JJ, Dargie HJ. Circulating endothelin in acute ischaemic syndromes. Br Heart J. 1992 May;67(5):383-6.
36. Zeiher AM, Goebel H, Schächinger V, Ihling C. Tissue endothelin-1 immunoreactivity in the active coronary atherosclerotic plaque. A clue to the mechanism of increased vasoreactivity of the culprit lesion in unstable angina. Circulation. 1995 Feb 15;91(4):941-7.
37. Yoon MH, Reriani M, Mario G, Rihal C, Gulati R, Lennon R, Tilford JM, Lerman LO, Lerman A. Long-term endothelin receptor antagonism attenuates coronary plaque progression in patients with early atherosclerosis. Int J Cardiol. 2013 Sep 30;168(2):1316-21.
38. Davenport AP, Maguire JJ. Is endothelin-induced vasoconstriction mediated only by ETA receptors in humans? Trends Pharmacol Sci. 1994 Jan;15(1):9-11.
39. Haynes WG, Moffat S, Webb DJ. An investigation into the direct and indirect venoconstrictor effects of endothelin-1 and big endothelin-1 in man. Br J Clin Pharmacol. 1995 Oct;40(4):307-11.
40. Verhaar MC, Strachan FE, Newby DE, Cruden NL, Koomans HA, Rabelink TJ, Webb DJ. Endothelin-A receptor antagonist-mediated vasodilatation is attenuated by inhibition of nitric oxide synthesis and by endothelin-B receptor blockade. Circulation. 1998 Mar 3;97(8):752-6.
41. McCulloch KM, Docherty CC, Morecroft I, MacLean MR. EndothelinB receptor-mediated contraction in human pulmonary resistance arteries. Br J Pharmacol. 1996 Nov;119(6):1125-30.
42. Rafnsson A, Shemyakin A, Pernow J. Selective endothelin ETA and dual ET(A)/ET(B) receptor blockade improve endothelium-dependent vasodilatation in patients with type 2 diabetes and coronary artery disease. Life Sci. 2014 Nov 24;118(2):435-9.

43. MacCarthy PA, Pegge NC, Prendergast BD, Shah AM, Groves PH. The physiological role of endogenous endothelin in the regulation of human coronary vasomotor tone. J Am Coll Cardiol. 2001 Jan;37(1):137-43.
44. Halcox JP, Nour KR, Zalos G, Quyyumi AA. Coronary vasodilation and improvement in endothelial dysfunction with endothelin ET(A) receptor blockade. Circ Res. 2001 Nov 23;89(11):969-76. PubMed PMID: 11717152.
45. Halcox JP, Nour KR, Zalos G, Quyyumi AA. Endogenous endothelin in human coronary vascular function: differential contribution of endothelin receptor types A and B. Hypertension. 2007 May;49(5):1134-41. Epub 2007 Mar 12. PubMed PMID: 17353514.
46. Mather KJ, Lteif AA, Veeneman E, Fain R, Giger S, Perry K, Hutchins GD. Role of endogenous ET-1 in the regulation of myocardial blood flow in lean and obese humans. Obesity (Silver Spring). 2010 Jan;18(1):63-70.
47. Kaski JC, Elliott PM, Salomone O, Dickinson K, Gordon D, Hann C, Holt DW. Concentration of circulating plasma endothelin in patients with angina and normal coronary angiograms. Br Heart J. 1995 Dec;4(6):620-4.
48. Fujii H, Takiuchi S, Kamide K, Horio T, Niizuma S, Tanaka N, Hashimoto S, Nakatani S, Fukagawa M, Kawano Y. Clinical implications of assessing coronary flow velocity reserve and plasma endothelin-1 in hypertensive patients. Hypertens Res. 2005 Nov;28(11):911-6.
49. Johnson NP, Gould KL. Clinical evaluation of a new concept: resting myocardial perfusion heterogeneity quantified by markovian analysis of PET identifies coronary microvascular dysfunction and early atherosclerosis in 1,034 subjects. J Nucl Med. 2005 Sep;46(9):1427-37.
50. Loghin C, Sdringola S, Gould KL. Does coronary vasodilation after adenosine override endothelin-1-induced coronary vasoconstriction? Am J Physiol Heart Circ Physiol. 2007 Jan;292(1):H496-502.

51. Johnson NP, Gould KL. Physiology of endothelin in producing myocardial perfusion heterogeneity: a mechanistic study using darusentan and positron emission tomography. J Nucl Cardiol. 2013 Oct;20(5):835-44.
52. Newby DE, Flint LL, Fox KA, Boon NA, Webb DJ. Reduced responsiveness to endothelin-1 in peripheral resistance vessels of patients with syndrome X. J Am Coll Cardiol. 1998 Jun;31(7):1585-90. PubMed PMID: 9626838.
53. Reriani M, Raichlin E, Prasad A, Mathew V, Pumper GM, Nelson RE, Lennon R, Rihal C, Lerman LO, Lerman A. Long-term administration of endothelin receptor antagonist improves coronary endothelial function in patients with early atherosclerosis. Circulation. 2010 Sep 7;122(10):958-66.
54. Johnson NP, Gould KL. Physiology of endothelin in producing myocardial perfusion heterogeneity: a mechanistic study using darusentan and positron emission tomography. J Nucl Cardiol. 2013 Oct;20(5):835-44.
55. Papadogeorgos NO, Bengtsson M, Kalani M. Selective endothelin A-receptor blockade attenuates coronary microvascular dysfunction after coronary stenting in patients with type 2 diabetes. Vasc Health Risk Manag. 2009;5:893-9.
56. Mancia G, Fagard R, Narkiewicz K, et al. 2013 ESH/ESC guidelines for the management of arterial hypertension: the Task Force for the Management of Arterial Hypertension of the European Society of Hypertension (ESH) and of the European Society of Cardiology (ESC). Eur Heart J. 2013 Jul;34(28):2159-219. doi: 10.1093/eurheartj/eht151. Epub 2013 Jun 14. PubMed PMID: 23771844.
57. McMurray JJ, Adamopoulos S, Anker SD, et al; ESC Committee for Practice Guidelines. ESC Guidelines for the diagnosis and treatment of acute and chronic heart failure 2012: The Task Force for the Diagnosis and Treatment of Acute and Chronic Heart Failure 2012 of the European Society of Cardiology. Developed in collaboration with the Heart Failure Association (HFA) of the ESC. Eur Heart J. 2012 Jul;33(14):1787-847. doi:

10.1093/eurheartj/ehs104. Epub 2012 May 19. Erratum in: Eur Heart J. 2013 Jan;34(2):158. PubMed PMID: 22611136.

58. Galiè N, Humbert M, Vachiery JL, et al; Authors/Task Force Members. 2015 ESC/ERS Guidelines for the diagnosis and treatment of pulmonary hypertension: The Joint Task Force for the Diagnosis and Treatment of Pulmonary Hypertension of the European Society of Cardiology (ESC) and the European Respiratory Society (ERS)Endorsed by: Association for European Paediatric and Congenital Cardiology (AEPC), International Society for Heart and Lung Transplantation (ISHLT). Eur Heart J. 2015 Aug 29. pii: ehv317. [Epub ahead of print] PubMed PMID: 26320113.
59. Maguire JJ, Davenport AP. Endothelin@25 - new agonists, antagonists, inhibitors and emerging research frontiers: IUPHAR Review 12. Br J Pharmacol. 2014 Dec;171(24):5555-72.
60. Gould KL. Does coronary flow trump coronary anatomy? JACC Cardiovasc Imaging. 2009 Aug;2(8):1009-23.
61. van de Hoef TP, Siebes M, Spaan JA, Piek JJ. Fundamentals in clinical coronary physiology: why coronary flow is more important than coronary pressure. Eur Heart J. 2015 Jun 1. pii: ehv235. [Epub ahead of print] Review. PubMed PMID: 26033981.
62. Barbato E, Aarnoudse W, Aengevaeren WR, Werner G, Klauss V, Bojara W, Herzfeld I, Oldroyd KG, Pijls NH, De Bruyne B; Week 25 study group. Validation of coronary flow reserve measurements by thermodilution in clinical practice. Eur Heart J. 2004;25:219-23.
63. Escaned J, Flores A, Garcia-Pavia P, et al. Assessment of microcirculatory remodeling with intracoronary flow velocity and pressure measurements: validation with endomyocardial sampling in cardiac allografts. Circulation 2009;120:1561e8.
64. Tsagalou EP, Anastasiou-Nana M, Agapitos E, Gika A, Drakos SG, Terrovitis JV, Ntalianis A, Nanas JN. Depressed coronary flow reserve is associated with decreased myocardial capillary density in patients with heart failure due to idiopathic dilated cardiomyopathy. J Am Coll Cardiol. 2008 Oct 21;52(17):1391-8.

65. Pepine CJ, Anderson RD, Sharaf BL, Reis SE, Smith KM, Handberg EM, Johnson BD, Sopko G, Bairey Merz CN. Coronary microvascular reactivity to adenosine predicts adverse outcome in women evaluated for suspected ischemia results from the National Heart, Lung and Blood Institute WISE (Women's Ischemia Syndrome Evaluation) study. J Am Coll Cardiol. 2010 Jun 22;55(25):2825-32. doi:0.1016/j.jacc.2010.01.054. PubMed PMID: 20579539; PubMed Central PMCID: PMC2898523.
66. Han SH, Bae JH, Holmes DR Jr, Lennon RJ, Eeckhout E, Barsness GW, Rihal CS, Lerman A. Sex differences in atheroma burden and endothelial function in patients with early coronary atherosclerosis. Eur Heart J. 2008 Jun;29(11):1359-69.
67. Lee BK, Lim HS, Fearon WF, Yong AS, Yamada R, Tanaka S, Lee DP, Yeung AC, Tremmel JA. Invasive evaluation of patients with angina in the absence of obstructive coronary artery disease. Circulation. 2015 Mar 24;131(12):1054-60.
68. Kobayashi Y, Fearon WF, Honda Y, Tanaka S, Pargaonkar V, Fitzgerald PJ, Lee DP, Stefanick M, Yeung AC, Tremmel JA. Effect of Sex Differences on Invasive Measures of Coronary Microvascular Dysfunction in Patients With Angina in the Absence of Obstructive Coronary Artery Disease. JACC Cardiovasc Interv. 2015 Sep;8(11):1433-41.
69. Fearon WF, Balsam LB, Farouque HM, Caffarelli AD, Robbins RC, Fitzgerald PJ, Yock PG, Yeung AC. Novel index for invasively assessing the coronary microcirculation. Circulation. 2003 Jul 1;107(25):3129-32
70. Yong AS, Layland J, Fearon WF, Ho M, Shah MG, Daniels D, Whitbourn R, Macisaac A, Kritharides L, Wilson A, Ng MK. Calculation of the index of microcirculatory resistance without coronary wedge pressure measurement in the presence of epicardial stenosis. JACC Cardiovasc Interv. 2013 Jan;6(1):53-8.
71. Melikian N, Vercauteren S, Fearon WF, Cuisset T, MacCarthy PA, Davidavicius G, Aarnoudse W, Bartunek J, Vanderheyden M, Wyffels E, Wijns W, Heyndrickx GR, Pijls NH, de Bruyne B. Quantitative assessment of coronary microvascular function in patients with and without

epicardial atherosclerosis. EuroIntervention. 2010 Apr;5(8):939-45. doi: 10.4244/. PubMed PMID: 20542779.

72. McGeoch R, Watkins S, Berry C, Steedman T, Davie A, Byrne J, Hillis S, Lindsay M, Robb S, Dargie H, Oldroyd KG The index of microcirculatory resistance measured acutely predicts the extent and severity of myocardial infarction in patients with ST-segment elevation myocardial infarction. JACC Cardiovasc Interv. 2010 Jul;3(7):715-22.
73. Payne AR* Berry C* Doolin O, McEntegart M, Petrie MC, Lindsay MM, Hood S, Carrick D, Tzemos N, Weale P, McComb C, Foster J, Ford I, Oldroyd KG. Microvascular Resistance Predicts Myocardial Salvage and Infarct Characteristics in ST-Elevation Myocardial Infarction. J Am Heart Assoc. 2012 Aug;1(4):e002246.
74. Fearon WF, Low AF, Yong AS, McGeoch R, Berry C, Shah MG, Ho MY, Kim HS, Loh JP, Oldroyd KG. Prognostic value of the index of microcirculatory resistance measured after primary percutaneous coronary intervention. Circulation. 2013 ;127(24):2436-41.
75. Layland JJ, Whitbourn RJ, Burns AT, Somaratne J, Leitl G, Macisaac AI, Wilson A. The index of microvascular resistance identifies patients with periprocedural myocardial infarction in elective percutaneous coronary intervention. Heart. 2012 Oct;98(20):1492-7.
76. van 't Veer M, Adjedj J, Wijnbergen I, Tóth GG, Rutten MC, Barbato E, van Nunen LX, Pijls NH, De Bruyne B. Novel monorail infusion catheter for volumetric coronary blood flow measurement in humans: in vitro validation. EuroIntervention. 2016 Aug 20;12(6):701-7. doi: 10.4244/EIJV12I6A114. PubMed PMID: 27542781.
77. Lee JM, Layland J, Jung JH, Lee HJ, Echavarria-Pinto M, Watkins S, Yong AS, Doh JH, Nam CW, Shin ES, Koo BK, Ng MK, Escaned J, Fearon WF, Oldroyd KG. Integrated Physiologic Assessment of Ischemic Heart Disease in Real-World Practice Using Index of Microcirculatory Resistance and Fractional Flow Reserve: Insights From the International Index of Microcirculatory Resistance Registry. Circ Cardiovasc Interv. 2015 Nov;8(11). pii: e002857. doi: 10.1161/CIRCINTERVENTIONS.115.002857..

78. Layland J, Carrick D, McEntegart M, Ahmed N, Payne A, McClure J, Sood A, McGeoch R, MacIsaac A, Whitbourn R, Wilson A, Oldroyd KG, Berry C. The Vasodilatory Capacity of the Coronary Microcirculation is Preserved in Selected Patients with NSTEMI. Circ Cardiovasc Intervention 2013;6(3): 231-6

79. Pijls NH, De Bruyne B, Peels K, et al. Measurement of fractional flow reserve to assess the functional severity of coronary-artery stenoses. N Engl J Med 1996;334:1703-1708.

80. Tonino PA, de Bruyne B, Pijls NH, et al. Fractional flow reserve versus angiography for guiding percutaneous coronary intervention. N Engl J Med 2009;360:213–224

81. De Bruyne B, Pijls NH, Kalesan B, Barbato E, Tonino PA, Piroth Z, Jagic N, Mobius-Winckler S, Rioufol G, Witt N, Kala P, MacCarthy P, Engström T, Oldroyd KG, Mavromatis K, Manoharan G, Verlee P, Frobert O, Curzen N, Johnson JB, Jüni P, Fearon WF; FAME 2 Trial Investigators. Fractional flow reserve-guided PCI versus medical therapy in stable coronary disease. N Engl J Med. 2012; 367:991-1001.

82. van Nunen LX, Zimmermann FM, Tonino PAL, Barbato E, Baumbach A, Engstrøm T, Klauss V, MacCarthy PA, Manoharan G, Oldroyd KG, Ver Lee PN, van't Veer M, Fearon WF, De Bruyne B, Pijls NHJ for the FAME Study Investigators. Fractional flow reserve versus angiography for guidance of PCI in patients with multivessel coronary artery disease (FAME): 5-year follow-up of a randomised controlled trial. Lancet 2015; August 30, http://dx.doi.org/10.1016/S0140-6736(15)00057-4

83. Greenwood JP, Ripley DP, Berry C, McCann GP, Plein S, Bucciarelli-Ducci C, Dall'Armellina E, Prasad A, Bijsterveld P, Foley JR, Mangion K, Sculpher M, Walker S, Everett CC, Cairns DA, Sharples LD, Brown JM; CE-MARC 2 Investigators. Effect of Care Guided by Cardiovascular Magnetic Resonance, Myocardial Perfusion Scintigraphy, or NICE Guidelines on Subsequent Unnecessary Angiography Rates: The CE-MARC 2 Randomized Clinical Trial. JAMA. 2016 Aug 29. doi: 10.1001/jama.2016.12680. [Epub ahead of print] PubMed PMID: 27570866.

84. Berry C et al. A randomised controlled trial of the effect of remote ischaemic conditioning on coronary endothelial function in patients with angina. https://clinicaltrials.gov/ct2/show/NCT02666235

85. Newby DE. Intracoronary infusions and the assessment of coronary blood flow in clinical studies. Heart. 2000 Aug;84(2):118-20.

86. http://www.nhs.uk/medicine-guides/pages/MedicineOverview.aspx?condition=Eye%20surgery%20and%20procedures&medicine=Acetylcholine%20chloride&preparationAcetylcholine%20chloride%2020mg%20powder%20and%20solvent%20for%20solution%20for%20intraocular%20irrigation%20vials

87. Panting JR, Gatehouse PD, Yang GZ, Grothues F, Firmin DN, Collins P, Pennell DJ. Abnormal subendocardial perfusion in cardiac syndrome X detected by cardiovascular magnetic resonance imaging. N Engl J Med. 2002 Jun 20;346(25):1948-53. PubMed PMID: 12075055.

88. Vermeltfoort IA, Bondarenko O, Raijmakers PG, Odekerken DA, Kuijper AF, Zwijnenburg A, van der Vis-Melsen MJ, Twisk JW, Beek AM, Teule GJ, van Rossum AC. Is subendocardial ischaemia present in patients with chest pain and normal coronary angiograms? A cardiovascular MR study. Eur Heart J. 2007 Jul;28(13):1554-8.

89. Hautvast GL, Chiribiri A, Lockie T, Breeuwer M, Nagel E, Plein S. Quantitative analysis of transmural gradients in myocardial perfusion magnetic resonance images. Magn Reson Med. 2011 Nov;66(5):1477-87. doi: 10.1002/mrm.22930. Epub 2011 May 31. PubMed PMID: 21630344.

90. Schuster A, Zarinabad N, Ishida M, Sinclair M, van den Wijngaard JP, Morton G, Hautvast GL, Bigalke B, van Horssen P, Smith N, Spaan JA, Siebes M, Chiribiri A, Nagel E. Quantitative assessment of magnetic resonance derived myocardial perfusion measurements using

advanced techniques: microsphere validation in an explanted pig heart system. J Cardiovasc Magn Reson. 2014 Oct 14;16:82.

91. Taylor AJ, Al-Saadi N, Abdel-Aty H, Schulz-Menger J, Messroghli DR, Gross M, Dietz R, Friedrich MG. Elective percutaneous coronary intervention immediately impairs resting microvascular perfusion assessed by cardiac magnetic resonance imaging. Am Heart J. 2006 Apr;151(4):891.e1-7.
92. Thomson LE, Wei J, Agarwal M, Haft-Baradaran A, Shufelt C, Mehta PK, Gill EB, Johnson BD, Kenkre T, Handberg EM, Li D, Sharif B, Berman DS, Petersen JW, Pepine CJ, Bairey Merz CN. Cardiac magnetic resonance myocardial perfusion reserve index is reduced in women with coronary microvascular dysfunction. A National Heart, Lung, and Blood Institute-sponsored study from the Women's Ischemia Syndrome Evaluation. Circ Cardiovasc Imaging. 2015 Apr;8(4). pii: e002481. doi: 10.1161/CIRCIMAGING.114.002481. PubMed PMID: 25801710; PubMed Central PMCID: PMC4375783.
93. Berry C, Radjenovic A, Corcoran D, et al. Study of the Vascular Effects of Serelaxin. https://clinicaltrials.gov/ct2/show/NCT01979614
94. Gulati M, Cooper-DeHoff RM, McClure C, Johnson BD, Shaw LJ, Handberg EM, Zineh I, Kelsey SF, Arnsdorf MF, Black HR, Pepine CJ, Merz CN. Adverse cardiovascular outcomes in women with non-obstructive coronary artery disease: a report from the Women's Ischemia Syndrome Evaluation Study and the St James Women Take Heart Project. Arch Intern Med 2009;169: 843–850.
95. Prescott E, Abildstrøm SZ, Aziz A, et al. Improving diagnosis and treatment of women with angina pectoris and microvascular disease: the iPOWER study design and rationale. Am Heart J. 2014;167(4):452-8.
96. Karamitsos TD, Arnold JR, Pegg TJ, Francis JM, Birks J, Jerosch-Herold M, Neubauer S, Selvanayagam JB. Patients with syndrome X have normal transmural myocardial perfusion

and oxygenation: a 3-T cardiovascular magnetic resonance imaging study. Circ Cardiovasc Imaging. 2012;5:194–200. doi: 10.1161/CIRCIMAGING.111.969667

97. Mygind ND, Michelsen MM, Pena AA, Faber R, Kastrup J, Prescott E. Prevalence of Coronary Microvascular Dysfunction in Women With Angina and No Obstructive Coronary Artery Disease: Preliminary Results From the iPower Study. Circulation. 2014; 130: A15094.

98. KittaY, Obata JE, NakamuraT, Hirano M, KodamaY, Fujioka D, Saito Y, Kawabata K, Sano K, Kobayashi T, Yano T, Nakamura K, Kugiyama K. Persistent impairment of endothelial vasomotor function has a negative impact on outcome in patients with coronary artery disease. J Am Coll Cardiol 2009;53:323–330.

99. Suwaidi JA, Hamasaki S, Higano ST, Nishimura RA, Holmes DR Jr, Lerman A. Long-term follow-up of patients with mild coronary artery disease and endothelial dysfunction. Circulation 2000;101: 948–954.

100. Johnson BD, Shaw LJ, Buchthal SD, Bairey Merz CN, Kim HW, Scott KN, Doyle M, Olson MB, Pepine CJ, den Hollander J, Sharaf B, Rogers WJ, Mankad S, Forder JR, Kelsey SF, Pohost GM; National Institutes of Health-National Heart, Lung, and Blood Institute. Prognosis in women with myocardial ischemia in the absence of obstructive coronary disease: results from the National Institutes of Health-National Heart, Lung, and Blood Institute-Sponsored Women's Ischemia Syndrome Evaluation (WISE). Circulation. 2004 Jun 22;109(24):2993-9.

101. STARD guidelines. http://www.equator-network.org/reporting-guidelines/stard/

102. Good Clinical Practice for Clinical Trials. https://www.gov.uk/good-clinical-practice-for-clinical-trials

103. Schulz KF, Altman DG, Moher D, CONSORT Group: CONSORT 2010 statement: updated guidelines for reporting parallel group randomised trials. PLoS Med 2010, 7:e1000251.

104. Calvert M, Blazeby J, Altman DG, Revicki DA, Moher D, Brundage MD; CONSORT PRO Group. Reporting of patient-reported outcomes in randomized trials: the CONSORT PRO extension. JAMA. 2013;309(8):814-822.

105. Clinical Trials Toolkit. National Institute for Health Research. http://www.ct-toolkit.ac.uk/

106. Carrick D, Behan M, Foo F, Christie J, Hillis WS, Norrie J, Oldroyd KG, Berry C. Usefulness of fractional flow reserve to improve diagnostic efficiency in patients with non-ST elevation myocardial infarction. Am J Cardiol. 2013 Jan 1;111(1):45-50. doi: 10.1016/j.amjcard.2012.08.046. Epub 2012 Oct 2. PubMed PMID: 23040601.

107. Layland J, Curzen N, Sood A, Balachandran K, Das R, Junejo S, Ahmed N, Lee M, Shaukat A, O'Donnell A, Nam J, Briggs A, Henderson R, McConnachie A, Berry C. Fractional Flow Reserve versus Angiography in Guiding Management to Optimise Outcomes in Non-ST-Segment Elevation Myocardial Infarction: The British Heart Foundation FAMOUS–NSTEMI randomised trial. Eur Heart J 2015; Jan 7;36(2):100-11; on-line FAST-TRACK PUBLICATION for HOTLINE SESSION at EUROPEAN SOCIETY OF CARDIOLOGY ANNUAL CONFERENCE, 1 September 2014.

108. Curzen N, Rana O, Nicholas Z, et al. Does Routine Pressure Wire Assessment Influence Management Strategy at Coronary Angiography for Diagnosis of Chest Pain?: The RIPCORD Study. Circ Cardiovasc Interv. 2014 Mar 18. [Epub ahead of print] PubMed PMID: 24642999.

109. Berry C, Corcoran D, Hennigan B, Watkins S, Layland J, Oldroyd KG. Fractional flow reserve-guided management in stable coronary disease and acute myocardial infarction: recent developments. Eur Heart J. 2015 Jun 2. pii: ehv206. [Epub ahead of print] Review. PubMed PMID: 26038588.

110. Vranckx P, Cutlip DE, McFadden EP, Kern MJ, Mehran R, Muller O. Coronary pressure-derived fractional flow reserve measurements: recommendations for

standardization, recording, and reporting as a core laboratory technique. Proposals for integration in clinical trials. Circ Cardiovasc Interv. 2012 Apr;5(2):312-7.

111. Spertus JA, Winder JA, Dewhurst TA, Deyo RA, Prodzinski J, McDonell M, Fihn SD. Development and evaluation of the Seattle Angina Questionnaire: a new functional status measure for coronary artery disease. J Am Coll Cardiol. 1995 Feb;25(2):333-41. PubMed PMID: 7829785.

112. EQ-5D™: a standardised instrument for use as a measure of health outcome. http://www.euroqol.org/about-eq-5d.html

113. Hicks KA, Tcheng JE, Bozkurt B, Chaitman BR, Cutlip DE, Farb A, Fonarow GC, Jacobs JP, Jaff MR, Lichtman JH, Limacher MC, Mahaffey KW, Mehran R, Nissen SE, Smith EE, Targum SL. 2014 ACC/AHA Key Data Elements and Definitions for Cardiovascular Endpoint Events in Clinical Trials: A Report of the American College of Cardiology/American Heart Association Task Force on Clinical Data Standards (Writing Committee to Develop Cardiovascular Endpoints Data Standards). J Am Coll Cardiol. 2014 Dec 29. pii: S0735-1097(14)07484-1.doi: 10.1016/j.jacc.2014.12.018.

114. Thygesen K, Alpert JS, Jaffe AS, Simoons ML, Chaitman BR, White HD; Writing Group on the Joint ESC/ACCF/AHA/WHF Task Force for the Universal Definition of Myocardial Infarction, Thygesen K, Alpert JS, White HD, Jaffe AS, Katus HA, Apple FS, Lindahl B, Morrow DA, Chaitman BA, Clemmensen PM, Johanson P, Hod H, Underwood R, Bax JJ, Bonow RO, Pinto F, Gibbons RJ, Fox KA, Atar D, Newby LK, Galvani M, Hamm CW, Uretsky BF, Steg PG, Wijns W, Bassand JP, Menasché P, Ravkilde J, Ohman EM, Antman EM, Wallentin LC, Armstrong PW, Simoons ML, Januzzi JL, Nieminen MS, Gheorghiade M, Filippatos G, Luepker RV, Fortmann SP, Rosamond WD, Levy D, Wood D, Smith SC, Hu D, Lopez-Sendon JL, Robertson RM, Weaver D, Tendera M, Bove AA, Parkhomenko AN, Vasilieva EJ, Mendis S; ESC Committee for Practice Guidelines (CPG). Third universal definition of myocardial infarction. Eur Heart J. 2012;33:2551-2567.

115. Wolk MJ, Scheidt S, Killip T. Heart failure complicating acute myocardial infarction. Circulation. 1972 May;45(5):1125-38.

116. Aho K, Harmsen P, Hatano S, Marquardsen J, Smirnov VE, Strasser T Cerebrovascular disease in the community: results of a WHO collaborative study. Bull World Health Organ. 1980;58(1):113-30.

117. Mehran R, Rao SV, Bhatt DL, Gibson CM, Caixeta A, Eikelboom J, Kaul S, Wiviott SD, Menon V, Nikolsky E, Serebruany V, Valgimigli M, Vranckx P, Taggart D, Sabik JF, Cutlip DE, Krucoff MW, Ohman EM, Steg PG, White H. Standardized bleeding definitions for cardiovascular clinical trials: a consensus report from the Bleeding Academic Research Consortium. Circulation. 2011 Jun 14;123(23):2736-47.

118. DAMOCLES Study Group, NHS Health Technology Assessment Programme. A proposed charter for clinical trial data monitoring committees: helping them to do their job well. Lancet. 2005 Feb 19-25;365(9460):711-22. PubMed PMID: 15721478.

119. Fearon WF, Bornschein B, Tonino PA, Gothe RM, Bruyne BD, Pijls NH, Siebert U; Fractional Flow Reserve Versus Angiography for Multivessel Evaluation (FAME) Study Investigators. Economic evaluation of fractional flow reserve-guided percutaneous coronary intervention in patients with multivessel disease. Circulation. 2010 Dec 14;122(24):2545-50. doi: 10.1161/CIRCULATIONAHA.109.925396. Epub 2010 Nov 29. PubMed PMID: 21126973.

120. Bedogni F, Indolfi C, Ribichini F, Verna E, Leone AM, Polimeni A, Bollati M, Biondi-Zoccai G, Testa L, Berti S; Italian Society of Invasive Cardiology (SICI-GISE). [Position paper on the theoretical basis, practical application and health economic evaluation of the functional assessment of coronary lesions endorsed by the Italian Society of Invasive Cardiology (SICI-GISE)]. G Ital Cardiol (Rome). 2015 Feb;16(2):116-28. doi: 10.1714/1798.19590. Italian. PubMed PMID: 25805097.

121. Nam J, Berry C, Henderson R, Briggs A. Economic modeling in randomized controlled trial (RCT)-based economic evaluations: empirical examples of its effect on the precision of economic and decision outcomes. Value in Health 2015;18(3):A7.

122. Nam J, Briggs A, Layland J, Curzen N, Sood A, Balachandran K, Das R, Junejo S, Ahmed N, Lee M, Shaukat A, O'Donnell A, Henderson R, McConnachie A, Berry C. Fractional Flow Reserve versus Angiography in Guiding Management to Optimise Outcomes in Non-ST-Segment Elevation Myocardial Infarction: A health economic analysis. Value in Health. 2015;18(3):A46.

123. Kent S, Briggs A Kent S, Briggs A, Berry C. Are value of information methods ready for prime time? An application to alternative treatment strategies for NSTEMI patients. International Journal of Technology Assessment in Health Care. 2013; 29:4 : 435–442.

124. McConnachie A, Walker A, Robertson M, Marchbank L, Peacock J, Packard CJ, Cobbe SM, Ford I (2014). Long-term impact on healthcare resource utilization of statin treatment, and its cost effectiveness in the primary prevention of cardiovascular disease: a record linkage study. Eur Heart J; 35: 290-298.

125. Goehler A, Geisler BP, Manne JM, Jahn B, Conrads-Frank A, Schnell-Inderst P, Gazelle GS, Siebert U. Decision-analytic models to simulate health outcomes and costs in heart failure: a systematic review. Pharmacoeconomics. 2011;29:753-69.

126. Campbell JD, McQueen RB, Libby AM, Spackman DE, Carlson JJ, Briggs A. Cost-Effectiveness Uncertainty Analysis Methods: A Comparison of One-Way Sensitivity, Analysis of Covariance, and Expected Value of Partial Perfect Information. Med Decis Making. 2015 Jul;35(5):596-607

127. Briggs A, Mihaylova B, Sculpher M, Hall A, Wolstenholme J, Simoons M, Deckers J, Ferrari R, Remme WJ, Bertrand M, Fox K; EUROPA Trial Investigators. Cost effectiveness of perindopril in reducing cardiovascular events in patients with stable coronary artery disease using data from the EUROPA study. Heart. 2007 Sep;93(9):1081-6.

128. Claxton KP, Sculpher MJ. Using value of information analysis to prioritise health research: some lessons from recent UK experience. Pharmacoeconomics. 2006;24(11):1055-68. Review. PubMed PMID: 17067191.

129. Sculpher MJ, Claxton K, Drummond M, McCabe C. Whither trial-based economic evaluation for health care decision making? Health Econ. 2006 Jul;15(7):677-87. PubMed PMID: 16491461.

130. Radjenovic A, Biglands JD, Larghat A, Ridgway JP, Ball SG, Greenwood JP, Jerosch-Herold M, Plein S. Estimates of systolic and diastolic myocardial blood flow by dynamic contrast-enhanced MRI. Magn Reson Med. 2010 Dec;64(6):1696-703. doi: 10.1002/mrm.22538. Epub 2010 Oct 6. PubMed PMID: 20928890.

131. Chen L, Adluru G, Schabel MC, McGann CJ, Dibella EV. Myocardial perfusion MRI with an undersampled 3D stack-of-stars sequence. Med Phys. 2012 Aug;39(8):5204-11.

132. Zeng GL, Li Y, DiBella EV. Non-Iterative Reconstruction with a Prior for Undersampled Radial MRI Data. Int J Imaging Syst Technol. 2013 Mar;23(1):53-58.

133. She H, Chen RR, Liang D, DiBella EV, Ying L. Sparse BLIP: Blind Iterative Parallel imaging reconstruction using compressed sensing. Magn Reson Med. 2014 Feb;71(2):645-60.

134. Lingala SG, DiBella E, Jacob M. Deformation corrected compressed sensing (DC-CS): a novel framework for accelerated dynamic MRI. IEEE Trans Med Imaging. 2015 Jan;34(1):72-85.

135. Huajun She, Rong-Rong Chen, DiBella EV, Schabel M, Ying L. Highly accelerated dynamic contrast-enhanced MRI with temporal constrained reconstruction. Conf Proc IEEE Eng Med Biol Soc. 2014;2014:2408-11.

136. Antman EM, Cooper HA, Gibson CM, de Lemos JA, McCabe CH, Giugliano RP, Coussement P, Murphy S, Scherer J, Anderson K, Van de Werf F, Braunwald E; Thrombolysis in Myocardial Infarction (TIMI) 14 Investigators. Determinants of improvement in epicardial

flow and myocardial perfusion for ST elevation myocardial infarction; insights from TIMI 14 and InTIME-II. Eur Heart J. 2002;23(12):928-33.

137. The Thrombolysis in Myocardial Infarction (TIMI) trial. Phase I findings. TIMI Study Group. N Engl J Med. 1985 Apr 4;312(14):932-6.

138. Gibson CM, Cannon CP, Murphy SA, Ryan KA, Mesley R, Marble SJ, McCabe CH, Van De Werf F, Braunwald E. Relationship of TIMI myocardial perfusion grade to mortality after administration of thrombolytic drugs. Circulation. 2000 Jan 18;101(2):125-30.

139. Antman EM, Cooper HA, Gibson CM, de Lemos JA, McCabe CH, Giugliano RP, Coussement P, Murphy S, Scherer J, Anderson K, Van de Werf F, Braunwald E; Thrombolysis in Myocardial Infarction (TIMI) 14 Investigators. Determinants of improvement in epicardial flow and myocardial perfusion for ST elevation myocardial infarction; insights from TIMI 14 and InTIME-II. Eur Heart J. 2002;23(12):928-33.

Gibson CM, Murphy SA, Rizzo MJ, Ryan KA, Marble SJ, McCabe CH, Cannon CP, Van de Werf F, Braunwald E. Relationship between TIMI frame count and clinical outcomes after thrombolytic administration. Thrombolysis In Myocardial Infarction (TIMI) Study Group. Circulation. 1999 Apr 20;99(15):1945-50.Gibson CM, de Lemos JA, Murphy SA, Marble SJ, McCabe CH, Cannon CP, Antman EM, Braunwald E; TIMI Study Group. Combination therapy with abciximab reduces angiographically evident thrombus in acute myocardial infarction: a TIMI 14 substudy. Circulation. 2001 May 29;103(21):2550-4.

1. Pijls NH, De Bruyne B, Peels K, et al. Measurement of fractional flow reserve to assess the functional severity of coronary-artery stenoses. N Engl J Med 1996;334:1703-8.
2. Ludmer PL, Selwyn AP, Shook TL, et al. Paradoxical vasoconstriction induced by acetylcholine in atherosclerotic coronary arteries. N Engl J Med 1986;315:1046-51.
3. Lee BK, Lim HS, Fearon WF, et al. Invasive evaluation of patients with angina in the absence of obstructive coronary artery disease. Circulation 2015;131:1054-60.

4. Hlatky MA, Boineau RE, Higginbotham MB, et al. A brief self-administered questionnaire to determine functional capacity (the Duke Activity Status Index). Am J Cardiol 1989;64:651-4.
5. Craig CL, Marshall AL, Sjostrom M, et al. International physical activity questionnaire: 12-country reliability and validity. Med Sci Sports Exerc 2003;35:1381-95.
6. Patel MR, Peterson ED, Dai D, et al. Low diagnostic yield of elective coronary angiography. N Engl J Med 2010;362:886-95.
7. Douglas PS, Patel MR, Bailey SR, et al. Hospital variability in the rate of finding obstructive coronary artery disease at elective, diagnostic coronary angiography. J Am Coll Cardiol 2011;58:801-9.
8. Marinescu MA, Loffler AI, Ouellette M, Smith L, Kramer CM, Bourque JM. Coronary microvascular dysfunction, microvascular angina, and treatment strategies. JACC Cardiovasc Imaging 2015;8:210-20.

Appendix 1. CorMicA Screening log

The screening log should be completed for all invasive angiograms.

Screening log number (eCRF)		
Age	…… years	-
Sex	Male	Female
Inclusion criterion		
Aged ≥18 years		
A clinically-indicated plan for invasive coronary angiography		
Symptoms of angina or angina-equivalent (according to the Rose- and Seattle Angina questionnaires).		
**Exclusion criteria**		
A non-coronary indication for invasive angiography e.g. valve disease		
During the angiogram: obstructive disease evident in a main coronary artery (diameter >2.5 mm), i.e. a coronary		

stenosis>50% or a fractional flow reserve (FFR) ≤0.80		
Sub-studies: Contra-indication to contrast-enhanced CMR e.g. severe renal dysfunction (GFR < 30 ml/min), non-CMR compatible pacemaker or defibrillator, asthma.		
Sub-studies: Skin Biopsy<br><br>Oral Anticoagulation/bleeding diathesis<br><br>Keloid scarring		

Appendix 2. Cardiologist questionnaire for use during the index admission

Pre-procedure

- **Catheter laboratory : pre-angiogram, pre- randomisation**

What is your assessment of the patient's symptoms?

Typical

Atypical

Non-anginal chest pain

Please indicate the likely diagnosis based on all of the available clinical information, including the results of any prior non-invasive tests ?

Diagnosis

1. What is the likelihood of coronary heart disease: *no, unlikely, probable, or yes*
2. What is the likelihood of angina due to obstructive coronary heart disease (i.e. >70% stenosis in a main branch or >50% in the left main stem): *no, unlikely, probable, or yes*.
3. What is the likelihood of angina due to a disorder of coronary function (i.e. microvascular angina or vasospastic angina): *no, unlikely, probable, or yes*.
4. What is the likelihood of a non-cardiac cause of chest pain: *no, unlikely, probable, or yes*.

- **Catheter laboratory : post-angiogram, before randomisation**

Diagnosis

1. What is the likelihood of coronary heart disease: *no, unlikely, probable, or yes*
2. What is the likelihood of angina due to obstructive coronary heart disease (i.e. >70% stenosis in a main branch or >50% in the left main stem): *no, unlikely, probable, or yes*.
3. What is the likelihood of angina due to a disorder of coronary function (i.e. microvascular angina or vasospastic angina): *no, unlikely, probable, or yes*.
4. What is the likelihood of a non-cardiac cause of chest pain: *no, unlikely, probable, or yes*.

Management

Onward treatment

1. Will the treatment plan change? *Yes or no*
2. If yes, in what way(s)? *medication, PCI, CABG*
3. Should preventive therapy i.e. anti-platelet & statin therapy, be included? *Yes or No*
4. 4.1 Should angina therapy be included? Yes or No

4.2 If yes to 4.1, will you now change the angina therapy? *Yes or No*

4.3 If yes to 4.1, is the angina treatment intended for a disorder of coronary function e.g. microvascular angina, vasospastic angina? *Yes or no.*

Onward investigations

1. Do you plan additional diagnostic tests? Yes or No
2. If yes, is it a cardiovascular test? Echocardiogram, CT scan, MRI, Ambulatory ECG
3. If yes, is it a non-cardiovascular test? Ultrasound, CT scan, MRI, endoscopy

Onward management

1. Cardiology follow-up: will you discharge the patient (i.e. no follow-up)? *Yes or NO*

2. Other specialty: will you refer the patient to a different speciality e.g. gastroenterology, or suggest to the GP to do so? *Yes or No*

- **Catheter laboratory/cardiology ward: post-angiogram, after coronary function test**

1. What is the likelihood of coronary heart disease: *no, unlikely, probable, or yes*
2. What is the likelihood of angina due to obstructive coronary heart disease (i.e. >70% stenosis in a main branch or >50% in the left main stem): *no, unlikely, probable, or yes*.
3. What is the likelihood of angina due to a disorder of coronary function (i.e. microvascular angina or vasospastic angina): *no, unlikely, probable, or yes*.
4. What is the likelihood of a non-cardiac cause of chest pain: *no, unlikely, probable, or yes*.

Management: post-angiogram, after coronary function test

Onward treatment

1. Will the treatment plan change? *Yes or no*
2. If yes, in what way(s)? *medication, PCI, CABG*
3. Should preventive therapy i.e. anti-platelet & statin therapy, be included? *Yes or No*
4. 4.1 Should angina therapy be included? Yes or No

4.2 If yes to 4.1, will you now change the angina therapy? *Yes or No*

4.3 If yes to 4.1, is the angina treatment intended for a disorder of coronary function e.g. microvascular angina, vasospastic angina? *Yes or no.*

Onward investigations

1. Do you plan additional diagnostic tests? Yes or No

2. If yes to 5.1, is it a cardiovascular test? Echocardiogram, CT scan, MRI, Ambulatory ECG

3. If yes to 5.1, is it a non-cardiovascular test? Ultrasound, CT scan, MRI, endoscopy

On ward management

1. Cardiology follow-up: will you discharge the patient (no follow-up)? *Yes or NO*

2. Other specialty: will you refer the patient to a different speciality e.g. gastroenterology, or suggest to the GP to do so? *Yes or No*

Appendix 3. Coronary function testing with acetylcholine (ACh)

Current local ACh use and logistical constraints: Acetylcholine (ACh) is a hygroscopic powder which is unstable in solution. ACh is rarely used at the Golden Jubilee National Hospital (GJNH) for diagnostic testing during standard care. To date, the local NHS 'Specials' manufacturer has prepared the three ACh solutions (10^{-6}M, 10^{-5}M & 10^{-4}M) required for the procedure as a 'special'. However, these pre-prepared solutions have a limited expiry of 30 hours and must be delivered using cold chain process in order to maintain the 2-8ºC storage conditions. Aseptic preparation of the solutions also has to be scheduled with a minimum turnaround time of 6 hours. Therefore in practice, ACh solutions required for coronary physiology examinations can only be used as part of a research study.

Use of licensed ACh: In the UK, licensed preparations of Ach are available such as Miochol-E. Miochol-E is presented as a vial containing 20mg acetylcholine chloride powder and solvent (10 ml) for intra-ocular instillation. In the research setting, Miochol-E or other similar preparations have been used as a starting point for preparation of ACh solutions for coronary physiology testing. From a product quality perspective, use of preparations such as Miochol-E ensures that Good Manufacturing Practice requirements are met and use of a terminally sterilised powder avoids the potential for contamination inherent with aseptic manipulation. However, there are potential risks associated with use of Miochol-E principally around the potential for dilution errors leading to ACh under and/or over dosing.

Proposed ACh kit for CorMicA study: A number of avenues were investigated by the research team in conjunction with colleagues at Pharmacy Production Unit, the local NHS manufacturing unit MIA (IMP) 24712. Preparation of solutions ready for use as has recently been done for the RIC-COR study (ClinicalTrials.gov Identifier: NCT02666235) were deemed impractical in the context of this pilot study which is intended to inform the design of a larger, multi-centre research study. Other options investigated included aseptic preparation

of solutions which could then be frozen extending the expiry date to 2 months. Again this presented multiple issues around transport and storage at study sites at -20°C and would require a thaw procedure prior to use.

Taking all potential risks into consideration, it was felt that the use of a structured method for dilution of licensed Miochol-E would meet the study requirements for a safe and sustainable presentation whilst ensuring product quality. In order to support dilution the proposal is that the site will be provided with a pre-prepared kit containing all the items required for dilution. The kit will contain the following:

- Miochol vial labelled specifically for use in the CorMicA study. The standard package insert and solvent will be removed.
- Worksheet including dilution protocol
- 1 ampoule of Sodium Chloride 0.9% 2ml
- 1 x 100ml, 250ml & 500ml Sodium Chloride 0.9% infusion bags
- 1ml, 2ml, 20ml, 30ml & 50ml(x3) syringes.
- 2 x Green Needles 21G & 5 White Needles 19G
- 3 infusion labels for 10^{-6}, 10^{-5} & 10^{-4} Molar ACh infusions
- 3 syringe labels for 10^{-6}, 10^{-5} & 10^{-4} Molar ACh bolus syringes ready for administration.
- 3 syringe caps
- 4 sterile alcohol swab

A pack containing licensed product ACh (eg. Miochol-E), all associated sundries (needles, syringes, labels, infusion bags) and a dilution worksheet with instructions will be manufactured and available in the cardiac catheter laboratory for immediate use during ad hoc diagnostic coronary angiography. The preparation steps will be informed by a Standard Operating Procedure (i.e. instructions) for preparation of the Ach test solutions. The study sponsors are of the opinion that using this standardised kit approach which incorporates detailed preparation instructions should minimise potential preparation errors associated with use of the licensed preparation and provide a product with sufficient shelf life (maximum of 24 months) for study purposes. If feasibility and our hypotheses are confirmed, then the study data may be used to support a licence change to enable parenteral ACh to be made more widely available for diagnostic testing during routine care. In this regard, the research has potential to be practice changing.

This text has been developed with the assistance of Dr. Elizabeth Douglas and Mr. Graham Conkie who are Pharmacists in NHS Greater Glasgow and Clyde Trials Pharmacy and Pharmacy Production Unit, respectively, and also with assistance from Mrs. Vicky McNulty, Trials Pharmacist in the Golden Jubilee National Hospital. The MHRA have classed the CorMicA study as a non-CTIMP.

Appendix 4. Definitions of coronary function test outcomes.

Outcome Measures (Angiography):

Coronary artery diameter change [Time Frame: Intra-procedure]

The net percentage change in mean coronary artery diameter (endothelial function) following intra-coronary administration of study drug (ACh or glyceryl trinitrate (GTN) compared to baseline.

Other Outcome Measures (Angiography):

•Mean percentage change in coronary lumen diameter (delta CAD) [Time Frame: Intra-procedure - After intracoronary infusion of intra-coronary acetylcholine]

The investigators aim to assess the percentage change from baseline in mean coronary lumen diameter, if any, in response to graded doses (10-6M, 10-5M, 10-4M) of intra-coronary acetylcholine infusion with each dose administered over a 2 minute period.

•Mean percentage coronary vasoconstriction [Time Frame: Intra-procedure - After intracoronary infusion of intra-coronary acetylcholine]

The investigators aim to assess the vasoconstrictor response, if any, to graded doses (10-6M, 10-5M, 10-4M) of intra-coronary acetylcholine infusion with each dose administered over a 2 minute period.

•Coronary endothelial dysfunction [Time Frame: Intra-procedure - After intracoronary infusion of intra-coronary acetylcholine]

A decrease in luminal diameter of >20% after intracoronary infusion of acetylcholine.

•Mean percentage change in coronary lumen diameter (delta CAD) [Time Frame: Intra-procedure - After intracoronary injection of glyceryl trinitrate]

The investigators aim to assess the percentage change from baseline in mean coronary lumen diameter, if any, following intracoronary injection of glyceryl trinitrate (200-400 micrograms)

•Mean percentage coronary vasodilatation [Time Frame: Intra-procedure - After intracoronary injection of GTN]

The investigators aim to assess the percentage change from baseline in mean coronary lumen diameter, if any, following intracoronary injection of glyceryl trinitrate (200-400 micrograms)

•Epicardial coronary artery spasm [Time Frame: Intra-procedure - During intra-coronary infusion of acetylcholine]

Epicardial coronary artery spasm is defined as a reduction in coronary diameter >90% following intracoronary acetylcholine in comparison with baseline resting condition following intracoronary glyceryl trinitrate (GTN) administration in any epicardial coronary artery segment together with symptoms and ST segment deviation on the ECG. Epicardial artery spasm may be focal or diffuse. Focal constriction was defined as a circumscribed transient vessel narrowing within the borders of 1 isolated or 2 neighbouring coronary segments. Diffuse constriction was diagnosed when the vessel narrowing was observed in ≥2 adjacent coronary segments. Proximal spasm is defined as vasoconstriction occurring in segments 1, 5, 6, or 11. Mid-vessel spasm will be recorded when occurring in segments 2, 3, or 7, whereas distal spasm is defined as that occurring in segments 4, 8, 9, 10, 12, 13, 14, or 15. This approach is in line with the guideline recommendations by COVADIS (Eur Heart J 2015; PMID: 26245334) and Sheikh et al[21].

•Microvascular spasm [Time Frame: Intra-procedure - During intra-coronary bolus of acetylcholine]

Microvascular spasm was diagnosed when angina occurred with either typical ischaemic ST-segment changes or reduction in TIMI flow grade in the absence of epicardial coronary constriction >90% diameter reduction.

•Functional microvascular angina to acetylcholine [Time Frame: Intra-procedure - During intra-coronary infusion of acetylcholine]

Functional microvascular angina was diagnosed when typical angina occurred during slow infusion of acetylcholine.

•ST-segment deviation [Time Frame: Intra-procedure - During intra-coronary infusion of acetylcholine]

ST-segment deviation from the iso-electric line due to ST-segment elevation or ST-segment depression, is a manifestation of myocardial ischaemia.

Specific endotypes of coronary artery function disorders according to these definitions include:

- Coronary artery vasospasm
- Impaired coronary artery vasorelaxation (CFR<2.0)
- Functional microvascular angina (Angina to Ach infusion or bolus)
- Impaired microvasodilatory capacity (RRR<2.0)
- Structural microvascular remodeling/increased coronary tone (IMR>25)

Appendix 5. Discharge guidance framework for GPs according to diagnosis.

<insert GJNH letterhead>

Diagnosis – Microvascular angina

Guideline recommended therapy

We have provided brief guidance to assist in managing microvascular angina based on the 2013 ESC guidelines & 2007 SIGN guidelines.[1,2]

Pharmacological management

- Calcium antagonists (e.g. **Verapamil** 40mg BD uptitrated weekly according to response)
    - Or Beta-blockers (e.g. 1.25mg **Bisoprolol** uptitrated or alternatively 3.125 mg of carvedilol twice daily with up-titration if feasible and appropriate)
- **Aspirin, Statin** or **ACEI** may be reasonable (depending on patient characteristics)
- Short-acting PRN nitrate (e.g. **Sublingual GTN**)
- **Nicorandil** if refractory symptoms (e.g. 5mg BD uptitrated weekly according to response)
- Xanthine inhibitors (aminophylline) – if refractory to all above

Non Pharmacological lifestyle & risk factor control

- **Smoking** "Smoking is a strong and independent risk factor for CVD and all smoking, including environmental smoking exposure, must be avoided in all patients with CVD"
- **Diet** "A healthy diet reduces CVD risk... Energy intake should be limited to the amount of energy needed to maintain (or obtain) a healthy weight—that is, a BMI <25 kg/m2."
- **Exercise** "moderate-to-vigorous intensity aerobic exercise training ≥3 times a week" (30 min)
- **Weight** "Weight reduction in overweight and obese people is recommended in order to achieve favourable effects on BP, dyslipidaemia and glucose metabolism"
- **Lipids** – "The goals of treatment are LDL-C below 1.8 mmol/L"
- **Hypertension** – "SBP/DBP to values within the range 130–139/80–85 mmHg"

- **Diabetes** “good control of glycated haemoglobin (HbA1c) to <7.0%...based on individual considerations.”
- **Psychosocial** “Patients should be assessed for psychosocial distress and appropriate care offered… Refer for psychotherapy, medication or collaborative care in the case of clinically significant symptoms of depression, anxiety and hostility.”
- **Cardiac rehabilitation** “A comprehensive risk-reduction regimen, integrated into comprehensive cardiac rehabilitation, is recommended to patients with CAD.”

1. Task Force M, Montalescot G, Sechtem U, Achenbach S, Andreotti F, Arden C, et al. 2013 ESC guidelines on the management of stable coronary artery disease: the Task Force on the management of stable coronary artery disease of the European Society of Cardiology. European heart journal. 2013;34(38):2949-3003.
2. SIGN. Guideline No. 96 - Management of stable angina. Edinburgh: Scottish Intercollegiate Guidelines Network (SIGN); 2007.

Appendix 5. Discharge guidance framework for GPs according to diagnosis.

<insert GJNH letterhead>

Guideline recommended therapy

Diagnosis – Vasospastic angina

We have provided brief guidance to assist in managing vasospastic angina based on the 2013 ESC guidelines & 2007 SIGN guidelines.[1,2]

Pharmacological management

- Non-dihydropyridine calcium channel blocker (e.g. **Verapamil** initially 40mg BD increasing at weekly intervals as tolerated upto 240-360mg daily)
- +/- **Long-acting nitrates** if symptoms ongoing (scheduled to cover the period of the day in which ischaemic episodes most frequently occur, in order to prevent nitrate tolerance.
- β-Blockers should be avoided.
- **Statin** therapy may be reasonable

Non Pharmacological lifestyle & risk factor control

- **Specific to vasospastic angina** – "exclude cocaine/amphetamine use"
- **Smoking** "Smoking is a strong and independent risk factor for CVD and all smoking, including environmental smoking exposure, must be avoided in all patients with CVD"
- **Diet** "A healthy diet reduces CVD risk... Energy intake should be limited to the amount of energy needed to maintain (or obtain) a healthy weight—that is, a BMI <25 kg/m2."
- **Exercise** "moderate-to-vigorous intensity aerobic exercise training ≥3 times a week" (30 min)
- **Weight** "Weight reduction in overweight and obese people is recommended in order to achieve favourable effects on BP, dyslipidaemia and glucose metabolism"
- **Lipids** – "The goals of treatment are LDL-C below 1.8 mmol/L"
- **Hypertension** – "SBP/DBP to values within the range 130–139/80–85 mmHg"

- **Diabetes** "good control of glycated haemoglobin (HbA1c) to <7.0%...based on individual considerations."
- **Psychosocial** "Patients should be assessed for psychosocial distress and appropriate care offered… Refer for psychotherapy, medication or collaborative care in the case of clinically significant symptoms of depression, anxiety and hostility."
- **Cardiac rehabilitation** "A comprehensive risk-reduction regimen, integrated into comprehensive cardiac rehabilitation, is recommended to patients with CAD."

1. Task Force M, Montalescot G, Sechtem U, Achenbach S, Andreotti F, Arden C, et al. 2013 ESC guidelines on the management of stable coronary artery disease: the Task Force on the management of stable coronary artery disease of the European Society of Cardiology. European heart journal. 2013;34(38):2949-3003.
2. SIGN. Guideline No. 96 - Management of stable angina. Edinburgh: Scottish Intercollegiate Guidelines Network (SIGN); 2007.

Appendix 6. Anxiety & Depression (Patient Health questionnaire 4) - PHQ4

PHQ-4				
**Over the <u>last 2 weeks</u>, how often have you been bothered by the following problems?**<br>*(Use "✔" to indicate your answer)*	Not at all	Several days	More than half the days	Nearly every day
1. Feeling nervous, anxious or on edge	0	1	2	3
2. Not being able to stop or control worrying	0	1	2	3
3. Little interest or pleasure in doing things	0	1	2	3
4. Feeling down, depressed, or hopeless	0	1	2	3

(*For office coding: Total Score T*____ = ____ + ____ + ____)

Appendix 7. Illness perception (Brief illness perception questionnaire) – B-IPQ

The Brief Illness Perception Questionnaire

For the following questions, please circle the number that best corresponds to your views:

How much does your illness affect your life?

0	1	2	3	4	5	6	7	8	9	10
no affect at all										severely affects my life

How long do you think your illness will continue?

0	1	2	3	4	5	6	7	8	9	10
a very short time										forever

How much control do you feel you have over your illness?

0	1	2	3	4	5	6	7	8	9	10
absolutely no control										extreme amount of control

How much do you think your treatment can help your illness?

0	1	2	3	4	5	6	7	8	9	10
not at all										extremely helpful

How much do you experience symptoms from your illness?

0	1	2	3	4	5	6	7	8	9	10
no symptoms at all										many severe symptoms

How concerned are you about your illness?

0	1	2	3	4	5	6	7	8	9	10
not at all concerned										extremely concerned

How well do you feel you understand your illness?

0	1	2	3	4	5	6	7	8	9	10
don't understand at all										understand very clearly

How much does your illness affect you emotionally? (e.g. does it make you angry, scared, upset or depressed?

0	1	2	3	4	5	6	7	8	9	10
not at all affected emotionally										extremely affected emotionally

Please list in rank-order the three most important factors that you believe caused <u>your illness</u>. *The most important causes for me:-*

1. ______________________________

2. ______________________________

3. ______________________________

Appendix 8. Treatment satisfaction (Treatment satisfaction questionnaire) – TSQM 9

1. How satisfied or dissatisfied are you with the ability of the medication to prevent or treat your condition?

□1 Extremely Dissatisfied

□2 Very Dissatisfied

□3 Dissatisfied

□4 Somewhat Satisfied

□5 Satisfied

□6 Very Satisfied

□7 Extremely Satisfied

2. How satisfied or dissatisfied are you with the way the medication relieves your symptoms?

□1 Extremely Dissatisfied

□2 Very Dissatisfied

□3 Dissatisfied

□4 Somewhat Satisfied

□5 Satisfied

□6 Very Satisfied

□7 Extremely Satisfied

3. How satisfied or dissatisfied are you with the amount of time it takes the medication to start working?

□1 Extremely Dissatisfied

□2 Very Dissatisfied

□3 Dissatisfied

□4 Somewhat Satisfied

□5 Satisfied

□6 Very Satisfied

□7 Extremely Satisfied

4. How easy or difficult is it to use the medication in its current form?

□1 Extremely Difficult

□2 Very Difficult

□3 Difficult

□4 Somewhat Easy

□5 Easy

□6 Very Easy

□7 Extremely Easy

5. How easy or difficult is it to plan when you will use the medication each time?

□1 Extremely Difficult

□2 Very Difficult

□3 Difficult

□4 Somewhat Easy

□5 Easy

□6 Very Easy

□7 Extremely Easy

6. How convenient or inconvenient is it to take the medication as instructed?

□1 Extremely Inconvenient

□2 Very Inconvenient

□3 Inconvenient

□4 Somewhat Convenient

□5 Convenient

□6 Very Convenient

□7 Extremely Convenient

7. Overall, how confident are you that taking this medication is a good thing for you?

□1 Not at All Confident

□2 A Little Confident

□3 Somewhat Confident

□4 Very Confident

□5 Extremely Confident

8. How certain are you that the good things about your medication outweigh the bad things?

□1 Not at All Certain

□2 A Little Certain

□3 Somewhat Certain

□4 Very Certain

□5 Extremely Certain

9. Taking all things into account, how satisfied or dissatisfied are you with this medication?

□1 Extremely Dissatisfied

□2 Very Dissatisfied

□3 Dissatisfied

□4 Somewhat Satisfied

□5 Satisfied

□6 Very Satisfied

□7 Extremely Satisfied

Appendix 9. Cover Letter for Follow up Questionnaire – CorMicA study

<insert GJNH letterhead>

"A study of whether tests & treatment of coronary function improve well-being of patients with angina."

"COROnary MICrovascular Angina (CorMicA): a randomised, controlled, pilot trial."

Dear <Insert Patient Name>,

We would be most grateful if you could kindly complete the attached questionnaire which is approximately six months after your coronary angiogram and enrolment in the CorMicA study. This questionnaire is a vital part of the research process and we take this opportunity to thank you once again for your assistance in completing the questions and returning this via the stamped addressed envelope. Additionally, we would like you to send a 'first voided' sample of urine for analysis. The instructions for how to take the urine sample are overleaf. Please list the medications that you take on a daily basis in the space below.

Please write today's date - ……………………………….

**Medicine**	**Dose**	**Medicine**	**Dose**

We are most grateful for your participation in this study. If you have any questions or concerns relating to the study please do get in touch with me on the details below.

Kind regards,

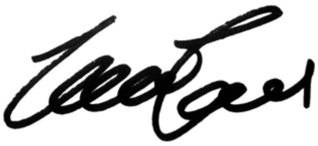

Dr Tom Ford (Clinical research fellow for Prof Colin Berry)

Department of Cardiology, Golden Jubilee National Hospital, Tel: 0141 951 5180.

INFORMATION ON URINE COLLECTION

You have been asked to collect a small sample of urine. Please read the following instructions carefully so that your sample is collected into the right container and tested in the near future after collection.

HOW DO I COLLECT THE URINE?

It is best to collect what is called a 'mid-stream urine'. Pass a small amount of urine into the toilet bowl, then pass a sample into the container. Do not allow the container to touch your body and keep fingers away from the rim and inner surface of the container. Fill the container three-quarters full.

WHAT TIME DO I COLLECT THE URINE?

You can collect your sample at any time, but the best sample is the first urine in the morning. This is because the first urine is concentrated and therefore is likely to give the best results. Some urine samples collected at other times may be too dilute and you will then have to collect another sample.

WHAT DO I COLLECT THE URINE INTO?

You should be given a container with a white top.

WHAT DO I THEN DO WITH THE URINE SAMPLE?

The urine sample pot should be firmly tightened and placed with the completed questionnaire into the prepaid return packaging.

DO I HAVE TO WRITE ANYTHING ON THE URINE CONTAINER LABEL?

YES! - You must write the date that you collected the sample on the label. Additionally please write your initials and date of birth to allow sample analysis.. The container comes prelabelled with your study ID

Appendix 10. DASI Questionnaire

DUKE ACTIVITY STATUS INDEX

Name: ______________________________ Hosp No: ______________________

Date: ___________________

Can You: (please circle yes or no)

1. Take care of yourself, that is, eat dress, bathe or use the toilet?	Yes/No
2. Walk indoors, such as around your house?	Yes/No
3. Walk a block or two on level ground?	Yes/No
4. Climb a flight of stairs or walk up a hill?	Yes/No
5. Run a short distance?	Yes/No
6. Do light work around the house like dusting or washing dishes?	Yes/No
7. Do moderate work around the house like vacuuming, sweeping floors or carrying groceries?	Yes/No
8. Do heavy work around the house like scrubbing floors or lifting or moving heavy furniture?	Yes/No
9. Do yard work like raking leaves, weeding or pushing a power mower?	Yes/No
10. Have sexual relations?	Yes/No
11. Participate in moderate recreational activities like golf, bowling, Dancing, doubles tennis or throwing a baseball or football?	Yes/No
12. Participate in strenuous sports like swimming, singles tennis Football, basketball or skiing?	Yes/No

Score=__________

Estimated VO_2peak=_______[METS]

INTERNATIONAL PHYSICAL ACTIVITY QUESTIONNAIRE (August 2002) – Short (7 days)

Think about all the **vigorous** activities that you did in the **last 7 days**. **Vigorous** physical activities refer to activities that take hard physical effort and make you breathe much harder than normal. Think *only* about those physical activities that you did for at least 10 minutes at a time.

1. During the **last 7 days**, on how many days did you do **vigorous** physical activities like heavy lifting, digging, aerobics, or fast bicycling?

   _____ **days per week**

   ☐ No vigorous physical activities → ***Skip to question 3***

2. How much time did you usually spend doing **vigorous** physical activities on one of those days?

   _____ **hours per day**

   _____ **minutes per day**

Think about all the **moderate** activities that you did in the **last 7 days**. **Moderate** activities refer to activities that take moderate physical effort and make you breathe somewhat harder than normal. Think only about those physical activities that you did for at least 10 minutes at a time.

3. During the **last 7 days**, on how many days did you do **moderate** physical activities like carrying light loads, bicycling at a regular pace, or doubles tennis? Do not include walking.

   _____ **days per week**

   ☐ No moderate physical activities → ***Skip to question 5***

4. How much time did you usually spend doing **moderate** physical activities on one of those days?

   _____ **hours per day**

   _____ **minutes per day**

SHORT LAST 7 DAYS SELF-ADMINISTERED version of the IPAQ. Revised August 2002.

Think about the time you spent **walking** in the **last 7 days**. This includes at work and at home, walking to travel from place to place, and any other walking that you have done solely for recreation, sport, exercise, or leisure.

5. During the **last 7 days**, on how many days did you **walk** for at least 10 minutes at a time?

   _____ **days per week**

   ☐ No walking → ***Skip to question 7***

6. How much time did you usually spend **walking** on one of those days?

   _____ **hours per day**

   _____ **minutes per day**

The last question is about the time you spent **sitting** on weekdays during the **last 7 days**. Include time spent at work, at home, while doing course work and during leisure time. This may include time spent sitting at a desk, visiting friends, reading, or sitting or lying down to watch television.

7. During the **last 7 days**, how much time did you spend **sitting** on a **week day**?

   _____ **hours per day**

   _____ **minutes per day**

This is the end of the questionnaire, thank you for participating.

SHORT LAST 7 DAYS SELF-ADMINISTERED version of the IPAQ. Revised August 2002.

Appendix 12. Cover Letter for 12 month questionnaire – CorMicA study

Golden Jubilee National Hospital

NHS National Waiting Times Centre

Cover Letter for Follow up Questionnaire – CorMicA study

"A study of whether tests & treatment of coronary function improve well-being of patients with angina."

"**COronary MICrovascular Angina (CorMicA): a randomised, controlled, pilot trial.**"

Dear Sir or Madam,

We would be most grateful if you could complete the attached questionnaire. Please bring this questionnaire with you to the 12-month follow up appointment at the Golden Jubilee National Hospital. This questionnaire is a vital part of our research and we take this opportunity to thank you once again for your assistance in completing the questions. If you are unable to attend please kindly return the completed questionnaire via the address below. Our research project is funded by the British Heart Foundation.

Additionally, please list the medications that you take on a daily basis in the space below, or alternatively, provide a copy of your current prescription.

Please write **today's date - ………………………………..**

**Medicine**	**Dose**	**Medicine**	**Dose**

We are most grateful for your participation in this study. If you have any questions or concerns relating to the study please do get in touch with me on the details below.

Kind regards,

Dr Tom Ford (Clinical research fellow for Prof Colin Berry)

CorMicA Returns, Department of Cardiology, Golden Jubilee National Hospital, Tel: 0141 951 5180.

CorMicA Cover letter 12 month follow up V1.0, 23/11/17 Randomisation ID ……………………

Appendix 13. Cover Letter for registry CorMicA Follow-up

Golden Jubilee National Hospital
NHS National Waiting Times Centre

Cover Letter for Follow up Questionnaire – CorMicA study

"A study of whether tests & treatment of coronary function improve well-being of patients with angina."

"**COROnary MICrovascular Angina (CorMicA): a randomised, controlled, pilot trial.**"

Dear Sir or Madam,

We would be most grateful if you could complete the attached questionnaire. This is a vital part of our BHF angina research study that you kindly agreed to participate in. We take this opportunity to thank you once again for your assistance in returning this via the stamped addressed envelope. Our research project is funded by the British Heart Foundation.

Additionally, please list the medications that you take on a daily basis in the space below.

Please write **today's date - ……………………………….**

Medicine	Dose	Medicine	Dose

We are most grateful for your participation in this study. If you have any questions or concerns relating to the study please do get in touch with me on the details below.

Kind regards,

Dr Tom Ford (Clinical research fellow for Prof Colin Berry)
Department of Cardiology, Golden Jubilee National Hospital, Tel: 0141 951 5180.

CorMicA Cover letter Registry follow up V1.0, 23/11/17 Study ID ……………………
